# Preclinical evaluation of the theranostic potential of ^89^Zr/^177^Lu-labeled anti-TROP-2 antibody in triple-negative breast cancer model

**DOI:** 10.1186/s41181-023-00235-x

**Published:** 2024-01-09

**Authors:** Yitian Wu, Tuo li, Xianzhong Zhang, Hongli Jing, Fang Li, Li Huo

**Affiliations:** 1 Medical Science Research Center, Peking Union Medical College Hospital, Chinese Academy of Medical Sciences, Beijing, 100730 China; 2 Department of Nuclear Medicine, State Key Laboratory of Complex Severe and Rare Diseases, Center for Rare Diseases Research Beijing Key Laboratory of Molecular Targeted Diagnosis and Therapy in Nuclear Medicine, Peking Union Medical College Hospital, Chinese Academy of Medical Science and Peking Union Medical College, Beijing, 100730 China

**Keywords:** Triple negative breast cancer (TNBC), TROP-2, ImmunoPET, Radioimmunotherapy (RIT), ^89^Zr, ^177^Lu, ADC

## Abstract

**Background:**

Triple-negative breast cancer (TNBC) is one of the most lethal malignant tumors among women, characterized by high invasiveness, high heterogeneity, and lack of specific therapeutic targets such as estrogen receptor, progesterone receptor, and human epidermal growth factor receptor 2. Trophoblast cell-surface antigen-2 (TROP-2) is a transmembrane glycoprotein over-expressed in 80% of TNBC patients and is associated with the occurrence, progress, and poor prognosis of TNBC. The TROP-2 targeted immunoPET imaging allows non-invasive quantification of the TROP-2 expression levels of tumors, which could help to screen beneficiaries most likely to respond to SG and predict the response. This study aimed to develop a ^89^Zr/^177^Lu-radiolabeled anti-TROP-2 antibody (NY003) for immunoPET and SPECT imaging, as well as radioimmunotherapy (RIT) in TROP-2 (+)TNBC tumor-bearing model. Based on the camelid antibody, we developed a TROP-2 targeted recombinant antibody NY003. NY003 was conjugated with DFO and DTPA for ^89^Zr and ^177^Lu radiolabelling, respectively. The theranostic potential of [^89^Zr]Zr-DFO-NY003/[^177^Lu]Lu-DTPA-NY003 was evaluated through immunoPET, SPECT imaging, and RIT studies in the subcutaneous TROP-2 positive TNBC xenograft mice model.

**Results:**

The high binding affinity of NY003 to TROP-2 was verified through ELISA. The radiochemical purity of [^89^Zr]Zr-DFO-NY003/[^177^Lu]Lu-DTPA-NY003 exceeded 95% and remained stable within 144h p.i. in vitro. ImmunoPET and SPECT imaging showed the specific accumulation of [^89^Zr]Zr-DFO-NY003/[^177^Lu]Lu-DTPA-NY003 in MDA-MB-231 tumors and gradually increased with the time tested, significantly higher than that in control groups (*P* < 0.05). The strongest anti-tumor efficacy was observed in the high-dose of [^177^Lu]Lu-DTPA-NY003 group, followed by the low-dose group, the tumor growth was significantly suppressed by [^177^Lu]Lu-DTPA-NY003, the tumor volumes of both high- and low-dose groups were smaller than the control groups (*P* < 0.05). Ex vivo biodistribution and histological staining verified the results of in vivo imaging and RIT studies.

**Conclusion:**

As a drug platform for radiotheranostics, ^89^Zr/^177^Lu-radiolabeled anti-TROP-2 antibody NY003 could not only non-invasively screen the potential beneficiaries for optimizing SG ADC treatment but also suppressed the growth of TROP-2 positive TNBC tumors, strongly supporting the theranostic potential of [^89^Zr]Zr-DFO-NY003/[^177^Lu]Lu-DTPA-NY003.

**Supplementary Information:**

The online version contains supplementary material available at 10.1186/s41181-023-00235-x.

## Introduction

Breast cancer has surpassed lung cancer as the first leading cause of cancer incidence and accounts for 685,000 deaths every year among women worldwide (Siegel et al. 2021a, b; Giaquinto et al. 2022). Various conventional and novel therapy options have been utilized in breast cancer, including surgery, chemotherapy, endocrine therapy, targeted therapy, radiation therapy, and immunotherapy (Baselga et al. 2012; Johnston 2010; Arteaga et al. 2011a, b). A lot of patients benefit from these treatments, the cure rate, disease-free survival, and overall survival have been improved significantly through standardized systemic treatment, but there are still some exceptions. Triple-negative breast cancer (TNBC) accounts for 15–20% of breast cancers, which is characterized by a lack of over-expression of estrogen receptor (ER), progesterone receptor (PR), and human epidermal growth factor receptor 2 (HER2) (Hudis and Gianni 2011). TNBC tends to be more aggressive and highly heterogeneous, which makes it the subtype with the worst prognosis among all breast cancers (Garrido-Castro et al. 2019; Gazinska et al. 2013). Given the absence of definite therapeutic targets for TNBC, systemic chemotherapy is still the cornerstone option (Santana-Davila and Perez 2010; Liu et al. 2014). However, less than 30% of advanced TNBC patients survive for 5 years after their diagnosis and systematic therapy (Jakabova et al. 2021). Immunotherapy is a novel approach for refractory malignant tumors that have emerged in recent years, unfortunately, a series of clinical research showed that TNBC has a relatively low response rate to the available immunotherapies (Schmid et al. 2020; Adams et al. 2020; Dong et al. 2023). Given the high lethality of TNBC and difficulties in therapy, new therapeutic strategies are critically demanded.

Antibody–drug conjugates (ADC) brought fresh air to those refractory patients by targeting cell surface antigens over-expressed in human cancer cells. ADC consists of an antibody, a chemical linker, and a cytotoxic drug, which can bind to the surface of the target through the antibody and then release a cytotoxic payload to achieve a targeted anticancer effect (Teicher and Chari 2011; Mahalingaiah et al. 2019; Shastry et al. 2022).

Trophoblast cell-surface antigen-2 (TROP-2) is a 46-kD transmembrane glycoprotein consisting of 323 amino acids that belongs to the TACSTD gene family and was first identified in human placental trophoblast, extra low or even absent expression was found in the membrane of adult somatic cells (Cubas et al. 2009). TROP-2 over-expression was found in various human epithelial cancers, such as breast, lung, oral, urothelial, prostate, pancreatic, cervical, ovarian, and gastrointestinal carcinomas, and is linked to an overall poor prognosis, leading to the development of TROP-2 targeted ADC treatment for malignant solid tumors (Ambrogi et al. 2014; Liu et al. 2013; Fang et al. 2009; Nakashima et al. 2004; Jiang et al. 2013; Mühlmann et al. 2009). Further, TROP-2 is over-expressed in the majority of TNBC patients (approximately 80%) (Son et al. 2018).

Sacituzumab govitecan (SG), consisting of a TROP-2 targeted humanized antibody (RS7), a carbonate linker, and a topoisomerase I inhibitor, was first approved by the FDA as a TROP-2 targeted ADC for advanced TNBC and urothelial cancer according to its definite therapeutic efficacy in the randomized phase III trial, although several toxicities were observed (Bardia et al. 2021; FDA 2021; TRODELVY® 2021; Tagawa et al. 2021). In addition to SG, there are still some other TROP-2 ADCs in clinical trials. Since the effectiveness of SG is primarily determined by the expression level of TROP-2, sensitive and precise ways to screen the patients with a high level of TROP-2 expression would optimize therapy. Conventional tumor biopsy and immunohistochemical (IHC) were commonly utilized in clinical procedures to identify the expression level of a certain protein. However, in addition to the invasiveness of biopsy, the accuracy of puncture biopsy is greatly affected by sampling due to the high internal heterogeneity of TNBC tumors.

ImmunoPET serves as an ideal non-invasive method for evaluating and monitoring the TROP-2 expression level of tumors before and during ADC treatment (Dongen et al. 2007) . A β+-emitter, Zirconium-89 (^89^Zr), with a relatively long half-life (78.4 h) and low β energy (395.5 keV) has been investigated and applied in antibody radiolabelling for PET imaging and could provide high-resolution images (Dietlein et al. 2022).

**Fig. 1:**
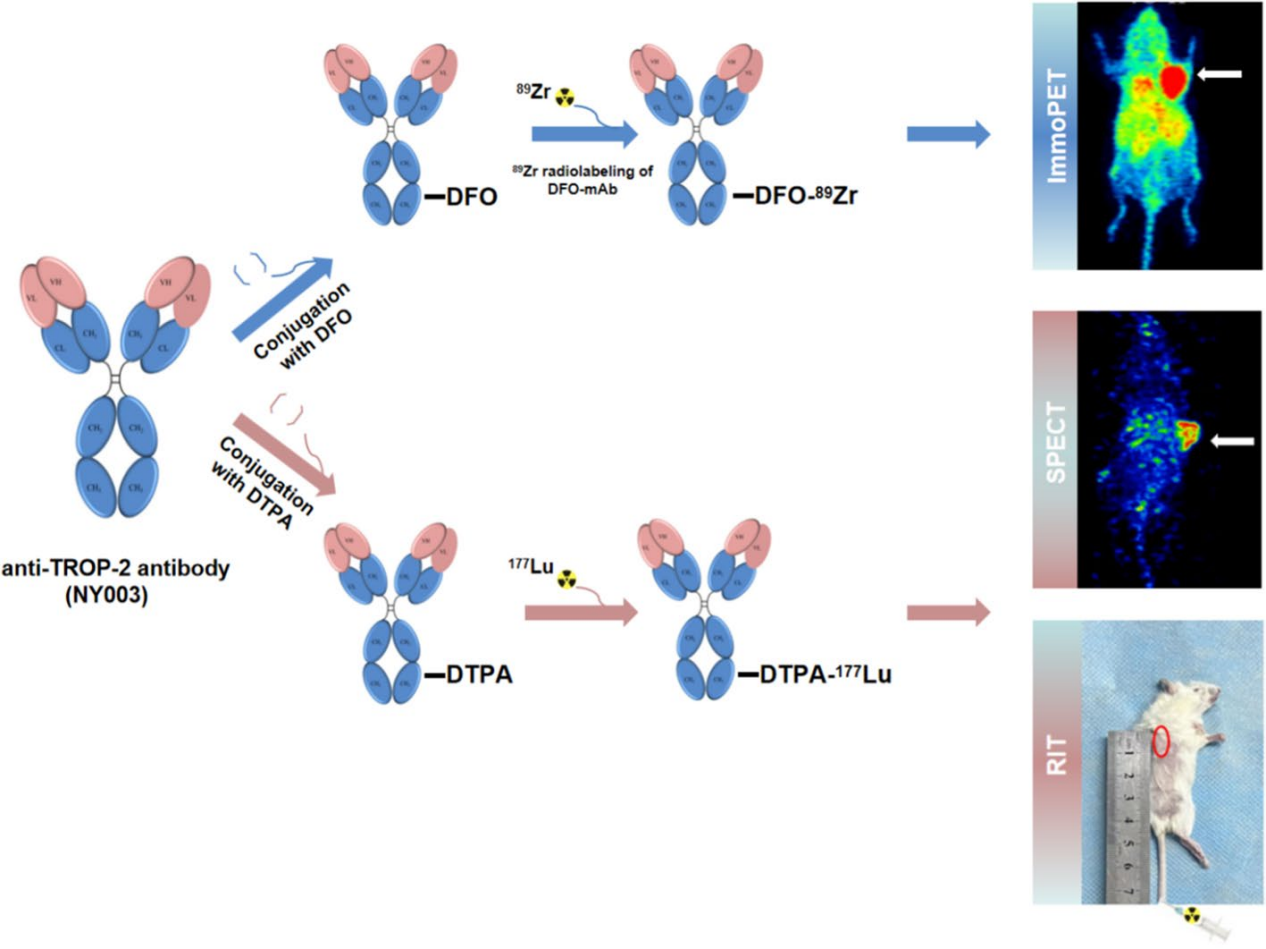
Scheme of the study. Anti-TROP-2 antibody NY003 was first conjugated with DFO and DTPA and then radiolabeled with ^89^Zr and ^177^Lu for immunoPET imaging and radioimmunotherapy (RIT) studies, respectively

In this study, we demonstrated the feasibility of immunoPET using ^89^Zr radiolabelling anti-TROP-2 antibody (NY003) on the TNBC xenograft mouse model. We also radiolabeled NY003 with ^177^Lu for SPECT imaging and further proved its therapeutic efficacy on TNBC tumors. All these study data identified that ^89^Zr/^177^Lu radiolabelling TROP-2 antibody provides a potential way for non-invasively imaging and screening the TROP-2 over-expressing tumors that would likely benefit from SG treatment and even radioimmunotherapy (RIT) (briefly summarized in Fig. 1).

## Materials and methods

All chemicals, reagents, and solvents for the synthesis and analysis were of analytical grade. All animal studies were performed according to a protocol approved by the Animal Care and Use Committee of Peking Union Medical College Hospital.

The goats were immunized with the extracellular region of human TROP-2 protein as antigen (500 μg), then the spleen cells were taken to prepare hybridoma cells until the serum antibody titer reached 100,0000. Positive hybridoma clones with stable and highly secreted anti-TROP-2 monoclonal antibody were screened by enzyme-linked immunosorbent assay (ELISA). The humanization of goat monoclonal antibodies was performed by genetic engineering.

### SDS-PAGE and Assessment of TROP-2 antibody binding affinity

SDS-PAGE was performed following the standard protocols. (Eaton et al. 2014) Protein concentration was calibrated to 1 μg/μL with a 5 × protein loading bufer (Beyotime Biotechnology). Ten microliters of samples were loaded into each well after boiling for 10 min, and 4–20% non-reducing sodium dodecyl sulfat-polyacrylamide gel electrophoresis (SDS-PAGE) was performed at 120 V for about 70 min.

The binding affinity of NY003 towards TROP-2 His protein was determined using ELISA assay. Briefly, TROP-2 His protein (antigen) was dissolved in 0.1 M carbonate buffer (pH 9.5) to 2 μg/mL, and 50 μL of the solution was added to each well to coat a 96-well polystyrene Stripwell microplate at 4 °C overnight and at room temperature for 1 h before the experiment. After washing, the rabbit anti-human monoclonal Trop2 (Abcam, 1:1000) was added into the wells and incubated at room temperature for 1 h. Then HRP goat anti-mouse IgG antibody (Thermo Scientific, 1:1500) was used for incubation for 1 h. After washing, TMB solution was added and OD values were detected at 450 nm. The EC50 value was calculated to evaluate the affinity of anti-TROP-2 antibodies to TROP-2.

### Cell lines and tumor-bearing model

Triple-negative breast cancer cell line MDA-MB-231 cell line (TROP-2 mild expression) was obtained from American Type Culture Collection (ATCC) and cultured in DMEM medium (Gibco) supplemented with 10% (v/v) fetal calf serum (Thermo Fisher), 2 mM L-Glutamine (Gibco), 200 μg/mL GENETICIN (Gibco), 100 U/mL Penicillin, and 100 μg/mL Streptomycin (Hyclone). Cells were incubated at 37 °C in a humidified atmosphere of 5% CO_2_. When the cell density was around 80 ~ 90%, cells were used for further experiments.

NSG mice (female, 4–6 weeks, 18–22 g) were obtained from Shanghai Model Organisms Co., Ltd (Shanghai, China) for subcutaneous TNBC cancer tumor model establishment. Tumor cells (approximately 1 × 10^7^) were suspended in a mixture (v/v, 1:1) of the medium and Matrigel (Corning Life Science) and then inoculated subcutaneously with cancer cells at the right flank of each mouse. The TROP-2 expression of tumors was identified by immunohistochemistry (IHC) assay. The tumor volume of each mouse were monitored everyday post-injection. When the xenograft tumor had grown up to 80–150 mm^3^, imaging and radiotherapy studies were conducted.

### Radiosynthesis, quality control, and in vitro stability

A self-designed radiolabelling module (SmartModule X) was employed for radiochemical synthesis.

The quality control of NY003, DFO-NY003, DTPA-NY003, [^89^Zr]Zr-DFO-NY003 and [^177^Lu]Lu-DTPA-NY003 was performed through HPLC. HPLC conditions: Tosoh TSKgel, G3000 SWXL, 300 X 7.8 mm molecular weight exclusion chromatography column; UV wavelength: 280nm; Equal elution flow rate: 0.8 ml/min; Mobile phase: 0.1M PBS + 0.5M NaCl solution.

**Scheme 1.**
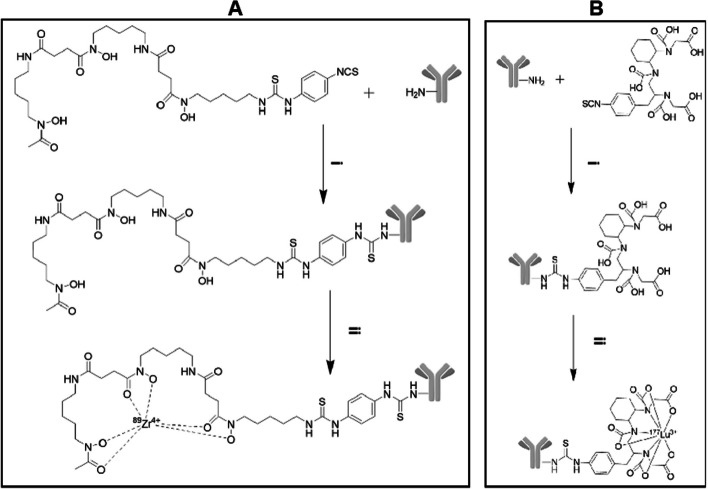
**A** Preparation of [^89^Zr]Zr-DFO-NY003. i: Na_2_CO_3_, DFO, NY003, room temperature, 60 min. ii: ^89^Zr, Na_2_CO_3_, acetic acid-sodium acetate buffer, Df-Bz-NCS-NY003, room temperature, 60 min; **B** Preparation of [^177^Lu]Lu-DTPA-NY003. i: Na_2_CO_3_, DTPA, NY003, room temperature, 180 min. ii: ^177^Lu, DTPA-NY003, room temperature, 30 min

The radiolabelling synthesis methods for [^89^Zr]Zr-DFO-NY003 and [^177^Lu]Lu-DTPA-NY003 were similar to the previous reports of NY004 and shown in Scheme 1 (Kong et al. 2023). Briefly, for [^89^Zr] radiolabelling, NY003 was dissolved in NaHCO3 solution (0.1 M, pH 8.4), and the pH value was adjusted to 9.0 with 0.1 M Na2CO3 solution, then, 2 mM DFO-Bz-NCS in dimethyl sulfoxide was added (molar ratio of DFO-Bz-NCS: NY003 = 5:1). After mixing and reacting at 37 °C for 60 min, the crude product DFO-Bz-NCS-NY003 was obtained, the PD10 column was used for purification. The prepared DFO-Bz-NCS-NY003 was added to the [^89^Zr] oxalic acid solution (with radioactivity of 3 mCi), which was neutralized to pH 7.0 with sodium carbonate solution. This solution was mixed evenly to form a reaction system and was left to react for 60 min at room temperature. The crude product was purified with the PD10 column, and the effluent was subsequently filtered using a 0.22 μm aseptic filter membrane. The radiochemical purity was performed using radio-instant thin-layer chromatography (Ecker & Ziegler, Mini-scan, citric acid/ sodium citrate buffer (0.5 M, pH 5) was used as the developer) and radio-HPLC (Angilent 1260 Infinity II, Tosoh TSKgel, G3000 SWXL, 300 × 7.8 mm molecular weight exclusion chromatography column; UV wavelength: 280 nm; Mobile phase: 0.1 M PBS + 0.5 M NaCl solution; Equal elution flow rate: 0.8 ml/min).

Equilibrate the PD10 column with acetic acid/sodium acetate buffer solution. NY003 was dissolved in NaHCO3 solution (0.1 M, pH 8.4), and the pH value was adjusted to 9.0 with 0.1 M Na2CO3 solution. 2 mM DTPA in dimethyl sulfoxide was added (molar ratio of DTPA: NY003 = 2–4:1). After mixing and reacting at 37 °C for 180 min, the crude product DFO-Bz-NCS-NY003 was obtained, the PD10 column was used for purification. For [^177^Lu] radiolabelling, the purified DTPA-NY003 was incubated with ^177^LuCl_3_ in a sodium acetate buffer (pH 4.5–5.5) at 37 °C for 30 min under constant shaking. The ratio of DTPA-NY003 and radionuclide was not below 300 MBq/mg. ^177^LuCl_3_ was obtained from Atomic High Tech (HTA) Co., Ltd. The radiochemical purity was performed using radio-instant thin-layer chromatography (BIOSCAN AR-2000, citric acid/ sodium citrate buffer (0.5 M, pH 5) was used as the developer) and radio-HPLC (Angilent 1200 Infinity, Tosoh TSKgel, G3000 SWXL, 300 × 7.8 mm molecular weight exclusion chromatography column; UV wavelength: 280 nm; Mobile phase: 0.1 M PBS + 0.5 M NaCl solution; Equal elution flow rate: 0.8 ml/min).

The in vitro stability of [^89^Zr]/[^177^Lu]-NY003 was determined by measuring radiochemical purity (RCP) in saline at 37 °C at different time intervals (0 h, 24 h, 48 h, 72 h, 96 h, 144 h for [^89^Zr]Zr-DFO-NY003 and 0 h, 48 h, 72 h and 144 h for [^177^Lu]Lu-DTPA-NY003) using radio-TLC.

### In vitro cell binding assay

MDA-MB-231 cells were seeded separately in 24 well plates (1 × 10^5^ cells in 1 mL medium/well) and incubated overnight, medium was changed 2 h before experiment. For binding experiment, cells were incubated with either [^89^Zr]Zr-DFO-NY003 or [^177^Lu]Lu-DTPA-NY003 (0.074 MBq/well in 100 μL) at 37 °C for 24, 48, 72, 96 h (6 wells/time point). After incubation, the medium was removed and the cells were washed twice with ice-cold phosphate-buffered saline (0.5 mL). Finally, the cells were lysed by NaOH (0.5 M, 0.5 mL). Co-incubation of excessive non-radiolabeled antibody and [^89^Zr]Zr-DFO-NY003 or [^177^Lu]Lu-DTPA-NY003 for 48 and 72 h was conducted as the blocking experiment. ACHN cells (TROP-2 negative) were used as the negative control. The radioactivity of cells was measured by a γ-counter. The studies were performed in triplicate and the results were reported as percentage injected activity (IA%/10^6^ cells).

### ImmunoPET, SPECT imaging, and biodistribution

The mice bearing MDA-MB-231 (n = 3/group) xenograft tumors were intravenously injected with [^89^Zr]Zr-DFO-NY003/[^177^Lu]Lu-DTPA-NY003 (5.55–7.4 MBq in 150 μL) via the tail vein, co-injection with non-labeled cold NY003 antibody (20 mg/kg) and [^89^Zr]Zr-DFO-NY003/[^177^Lu]Lu-DTPA-NY003 was administrated for blocking study. The mice were anesthetized with 3% (v/v) isoflurane and underwent Micro-PET (Pingseng Super Nova, China) or Micro-SPECT (MOLECUBES, Belgium) scans with continuous 1% (v/v) isoflurane. The images were acquired at different time points p.i. (7, 24, 48, 72, 96, and 144 h for PET and 1, 4, 12, 24, 48, 72, and 96 h for SPECT). As the negative control, [^89^Zr]-IgG (7.4 MBq in 150 μL) was injected into another group of mice (n = 3), and the Micro-PET schedule was performed as described before. Micro-PET image reconstruction was performed using the 3D OSEM PSF algorithm with five iterations. All the images were processed and analyzed using PMOD 4.2 software (PMOD Technologies Ltd., Switzerland). The co-registered CT scanning was used for photon attenuation correction in the SPECT data reconstruction. The decay-corrected images were reconstructed and analyzed using AMIDE software.

The normal NSG mice were injected with [^89^Zr]Zr-DFO-NY003/[^177^Lu]Lu-DTPA-NY003 (4 groups, n = 3/group, 0.55–0.74 MBq in 150 μL per mouse). Mice were sacrificed at different time points p.i. (consistent with the time points in PET and SPECT imaging), tumors and interested organs were harvested, weighed, and measured by a γ-counter, the results were expressed as the percentage of injected dose per gram (ID%/g).

### Therapy study

Mice with TROP-2 (+) MDA-MB-231 flank tumor models (5 groups, n = 8/group) were used to evaluate the anti-tumor efficacy of [^177^Lu]Lu-DTPA-NY003. The mice in the low-dose and high-dose groups were injected intravenously with 11.1 and 18.5 MBq (200 μL) of [^177^Lu]Lu-DTPA-NY003, respectively. The other three control groups were injected with ^177^LuCl_3_ solution, non-labeled NY003 antibody, or saline, respectively.

The body weights and tumor volumes of mice were measured every 2 days. The tumor volume was determined using V = width^2^ × length/2. Endpoint criteria were defined as weight loss ≥ 15%, tumor volume > 1800 mm^3^, active ulceration of the tumor, or abnormal behaviors. Besides, at 14 d post-injection, venous blood was drawn from the tail vein for the routine blood test.

### Histological staining

Hematoxylin–Eosin (H&E) staining of Normal organs and tissues (heart, liver, spleen, lung, kidney) and immunohistochemistry (IHC) assay of MDA-MB-231 xenograft tumor tissues were processed following standard procedures. IHC staining of tumor tissue was incubated with the primary antibody (NY003, 5 μg/mL) overnight at 4 °C. After washing, the secondary antibody (goat anti-rabbit IgG Fc-HRP antibody, Thermo) was incubated for 1 h at room temperature and washed with PBS for three times. All images were visualized and captured with an inverted microscopy. LeicaVERSA8 was used for panoramic imaging, and ImageJ software (National Institutes of Health, Bethesda, Maryland, USA) was used for image analysis.

At least 100 μL of venous blood was collected in an anticoagulant tube for whole blood cell analysis including white blood cell (WBC), red blood cell (RBC), mean corpuscular hemoglobin (MCH), platelet (PLT) and hemoglobin (HGB) at 14 d p.i..

### Statistical analysis

All quantitative data were expressed as mean ± SD. The unpaired Student’s *t* tests and repeated measures ANOVA were for for significance testing. A *P* value of < 0.05 was considered statistically significant. Statistical analyses were performed using SPSS software 22.0 (IBM Corp., Armonk, NY, USA) and Prism 8 software (GraphPad Software, San Diego, California).

## Results

### Construction, qualitative control, and binding affinity of antibody

**Fig. 2:**
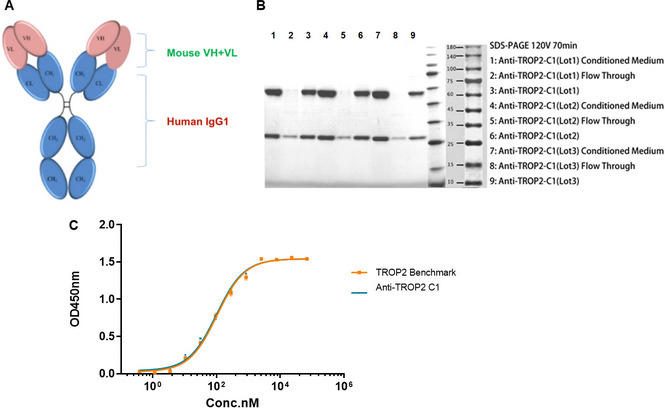
Molecular structure design and identification of NY003. **A** Structural sketch of the full-length antibody NY003. **B** SDS-PAGE analysis result of NY003. **C** Binding affinity via ELISA

High-affinity full-length anti-TROP-2 antibody NY003 was obtained by phage display technique. The molecular weight of NY003 was 146.06 kDa. R250 dyeing on the SDS-PAGE gel confirmed the purity (greater than 95%) and specificity of NY003. The EC50 value was calculated as 94.99 nM, indicating a high affinity of NY003 to TROP-2 (shown in Fig. 2).

### Radiosynthesis, quality control, and in vitro stability

We have successfully conducted the radiolabelling of NY003 with different radionuclides and different chelating linkers using a self-developed radiolabelling module (SmartModule-X), which was reported previously (Kong et al. 2023).

**Fig. 3:**
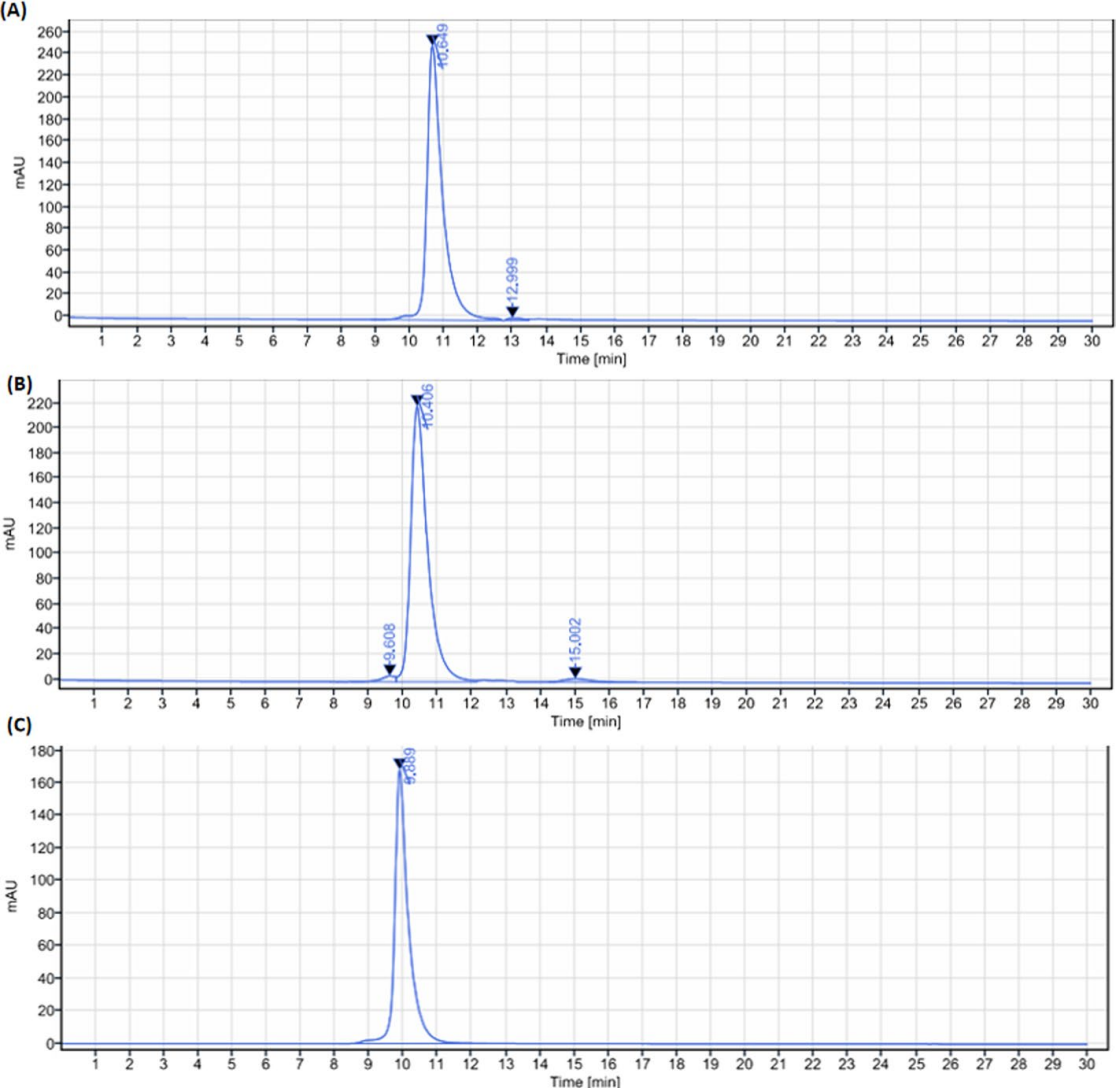
The HPLC chromatograms of NY003 (**A**), DFO-NY003 (**B**) and DTPA-NY003 (**C**). HPLC

The analytical HPLC chromatograms of NY003, DFO-NY003 and DTPA-NY003 were shown in Fig. 3. The chemical yield of DFO-NY003 and DTPA-NY003 was more than 85%, and the chemical purity was more than 95%.

**Fig. 4:**
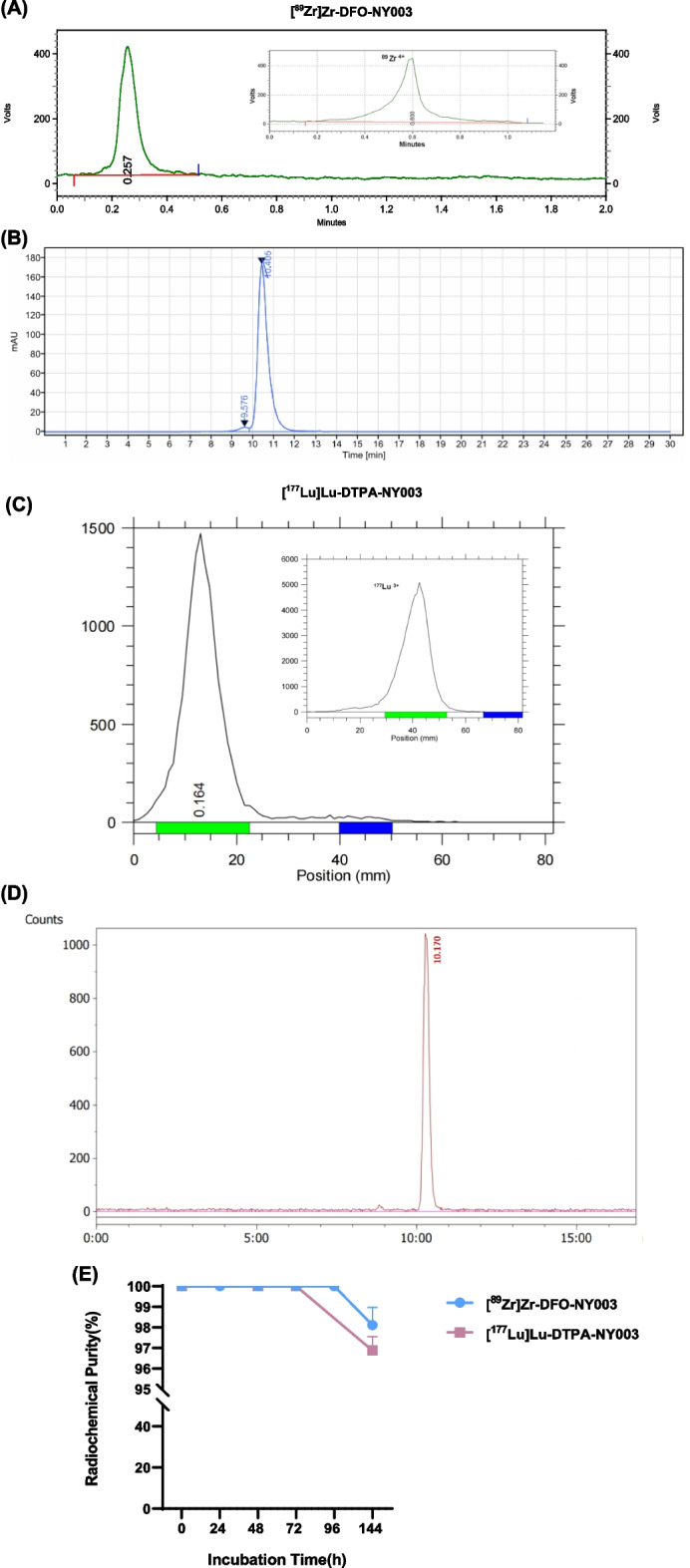
Integration diagram of analytical radio-TLC and radio-HPLC radioactive traces of [^89^Zr]Zr-DFO-NY003 (**A**, **B**) and [^177^Lu]Lu-DTPA-NY003 (**C**, **D**). The developer for TLC was citric acid/ sodium citrate buffer (0.5 M, pH 5). The mobile phase for HPLC was 0.1 M PBS + 0.5 M NaCl solution (**E**). In vitro stability of [^89^Zr]Zr-DFO-NY003 and [^177^Lu]Lu-DTPA-NY003 in saline

[^89^Zr]Zr-DFO-NY003 and [^177^Lu]Lu-DTPA-NY003 were prepared with a radiochemical purity (RCP) of more than 99% analyzed by radio-TLC and radio-HPLC. The retention time of [^89^Zr]Zr-DFO-NY003 and [^177^Lu]Lu-DTPA-NY003 were 0.257 min and 0.164 min in radio-TLC, 10.522 min and 10.177 min in radio-HPLC, respectively. While the retention time of ^89^Zr and ^177^Lu were 0.600 min and 0.511 min in radio-TLC, which could be distinguished easily. [^89^Zr]Zr-DFO-NY003/[^177^Lu]Lu-DTPA-NY003 were stable in vitro for at least 144 h (shown in Fig. 4), the chemicalpurity remained 100% within 72 h p.i. for [^177^Lu]Lu-DTPA-NY003 and 96 h p.i. for [^89^Zr]Zr-DFO-NY003, and decreased slightly afterwards, with the radiochemical purity of 98.11 ± 0.87 for [^89^Zr]Zr-DFO-NY003 and 96.88 ± 0.67 for [^177^Lu]Lu-DTPA-NY003 at 144 h p.i., respectively.

The non-decay-corrected radiochemical yields of [^89^Zr]Zr-DFO-NY003 and [^177^Lu]Lu-DTPA-NY003 were 56% and 96%, respectively.

conditions: Tosoh TSKgel, G3000 SWXL, 300 × 7.8 mm molecular weight exclusion chromatography column; UV wavelength: 280nm; Equal elution flow rate: 0.8 ml/min; Mobile phase: 0.1M PBS + 0.5M.

NaCl solution

### In vitro cellular studies

**Fig. 5:**
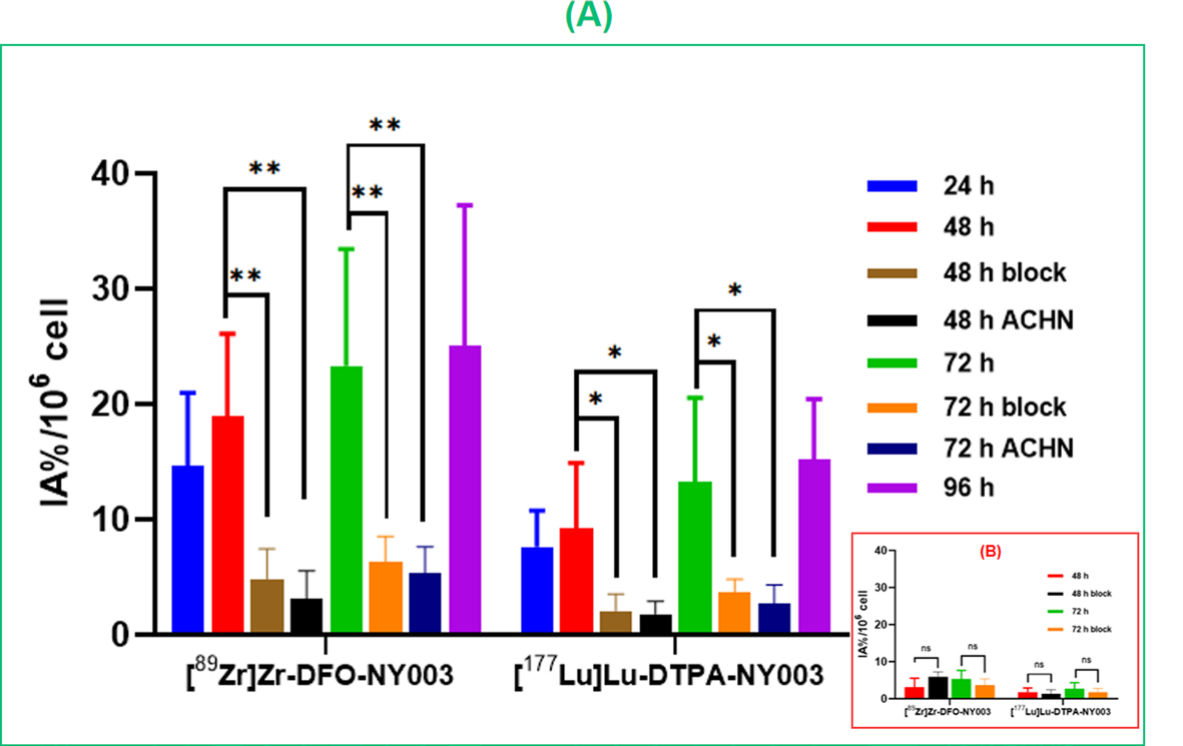
Cell uptake of [89Zr]Zr-DFO-NY003 and [177Lu]Lu-DTPA-NY003 in MDA-MB-231 (**A**) and ACHN (**B**) cells at different time points. (Block = co-incubation with non-radiolabeled NY003 and radiotracers for 48 and 72 h. Values were expressed as mean ± SD, n = 6. **Means *P* < 0.01, ns means not statistically significant)

MDA-MB-231 (TROP-2+) and ACHN (TROP-2−) cell lines were used to evaluate the specific cell binding affinity of radiotracers. As shown in Fig. 5A, [^89^Zr]Zr-DFO-NY003 and [^177^Lu]Lu-DTPA-NY003 revealed substantially higher uptake in MDA-MB-231 cells than ACHN cells at all incubation time points (*P* < 0.05). The uptake in MDA-MB-231 cells was increased within the time tested: from 14.67 ± 6.03 IA%/10^6^ at 24 h to 25.12 ± 12.12 IA%/10^6^ at 96 h for [^89^Zr]Zr-DFO-NY003, from 7.65 ± 3.12 IA%/10^6^ at 24 h to 15.21 ± 5.23 IA%/10^6^ at 96 h for [^177^Lu]Lu-DTPA-NY003. The uptake was significantly blocked by non-radiolabeled antibody. While, the uptake in ACHN cells was very low and could not be blocked by NY003 (Fig. 5B).

### *[*^*89*^*Zr]Zr-DFO-NY003 micro-PET imaging in TNBC models*

**Fig. 6:**
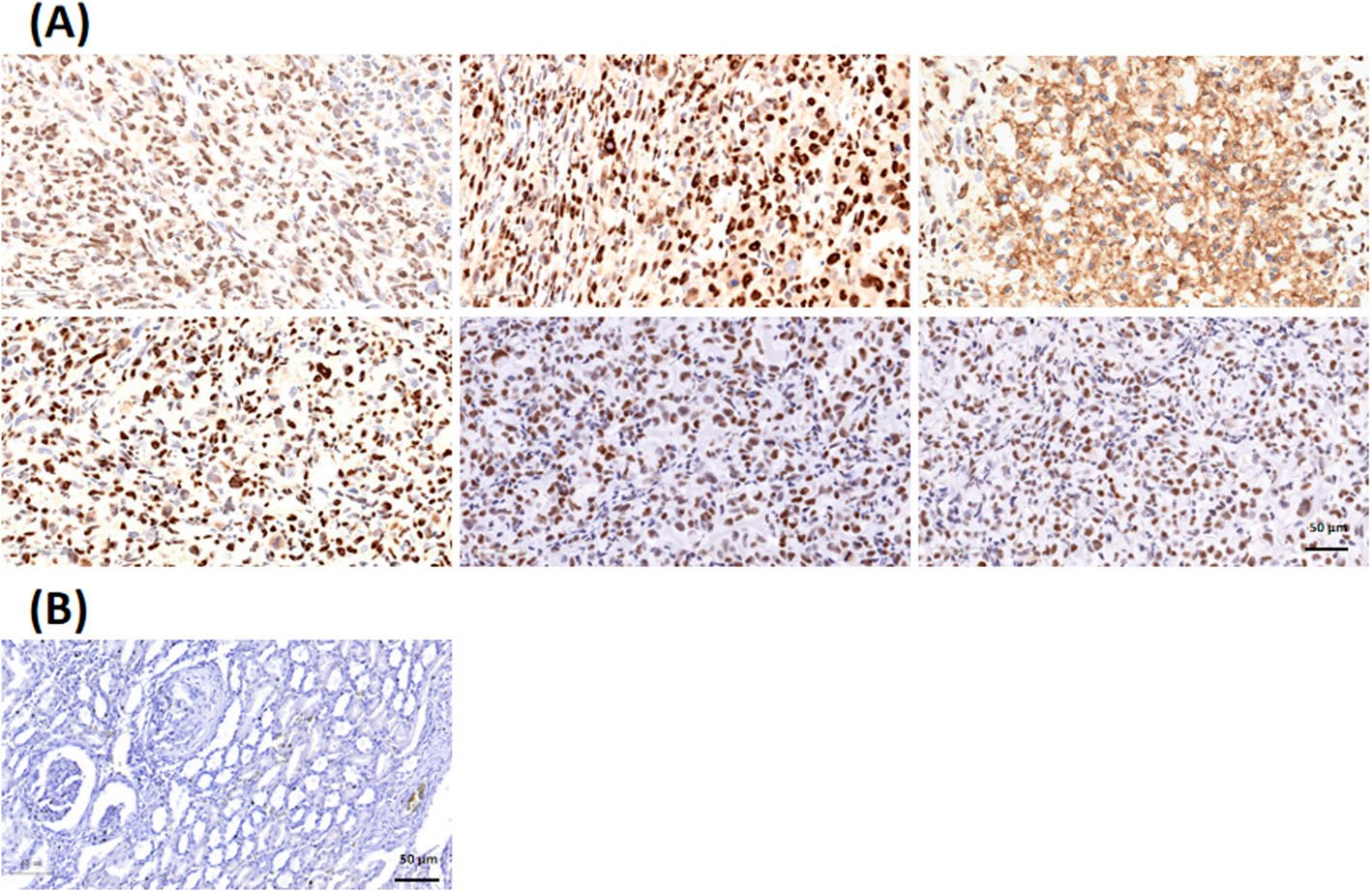
The representative images of TROP-2 IHC staining of TROP-2 positive MDA-MB-231 (**A**) and TROP-2 negative ACHN (**B**) xenograft tumors, illustrating the weak to moderate specific TROP-2 over-expression of MDA-MB-231 tumor samples

MDA-MB-231 xenograft tumor-bearing models were established for imaging and therapeutic efficacy investigation. The expression level of xenograft tumors was determined by IHC, all the samples demonstrated weak to moderate positive TROP-2 expression, with an IHC score of 1+ to 2+ (the representative images were shown in Fig. 6).

**Fig. 7:**
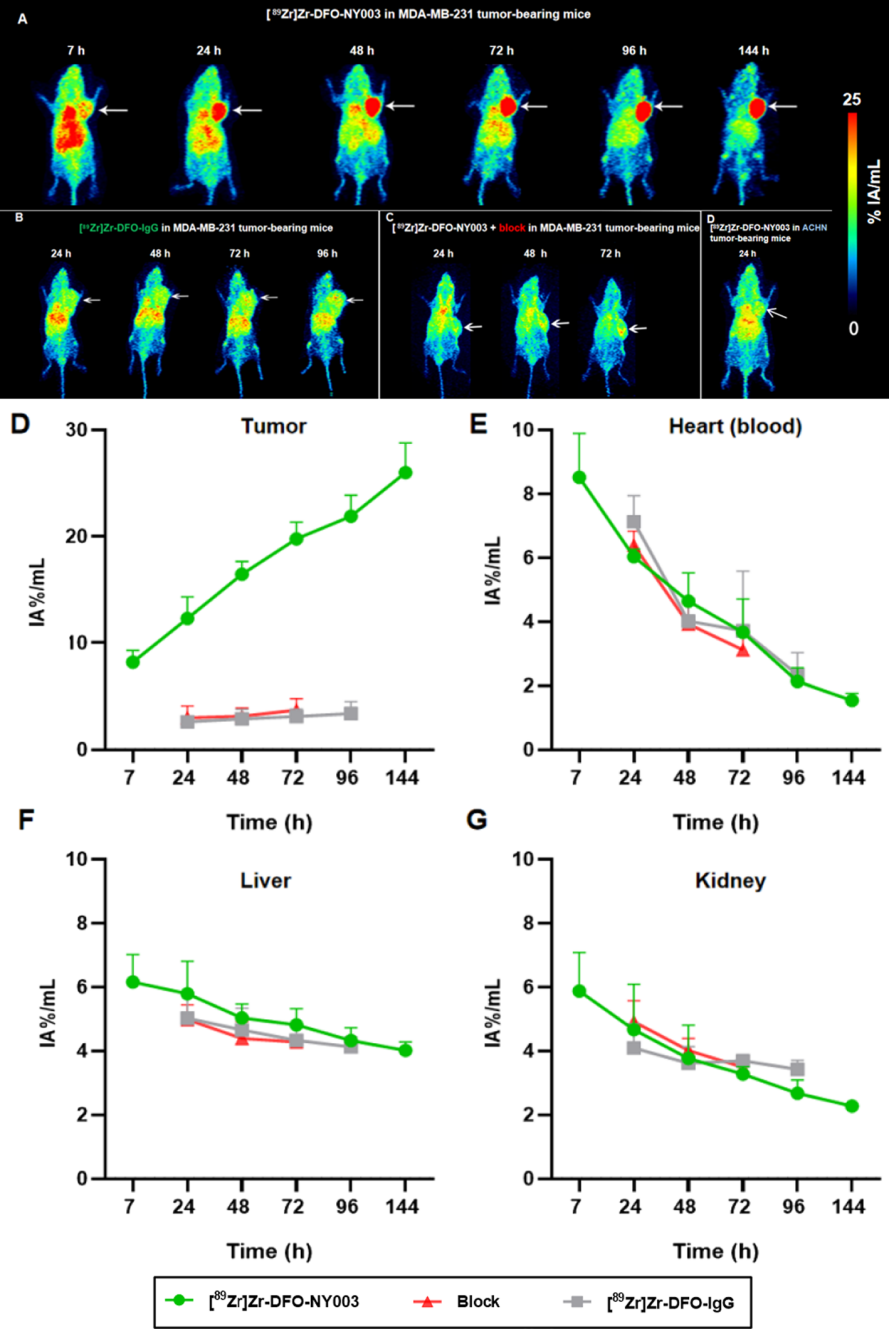
Micro-PET images in MDA-MB-231 tumor-bearing mice at different time points. **A** Representative MIP images of [^89^Zr]Zr-DFO-NY003 (5.55–7.4 MBq) in MDA-MB-231 model at different time points p.i.. **B** Representative MIP images of [^89^Zr]Zr-DFO-IgG (5.55 ~ 7.4 MBq) in the MDA-MB-231 model at different time points p.i.. **C** Representative MIP images of co-injection of [^89^Zr]Zr-DFO-NY003 (5.55 ~ 7.4 MBq) and cold NY003 in MDA-MB-231 model at different time points p.i.. **D–G** Quantitative ROI analysis showed that the tumor uptake of [^89^Zr]Zr-DFO-NY003 in MDA-MB-231 (*n* = 3) was significantly higher than that of blocking and [^89^Zr]Zr-DFO-IgG groups (*P* < 0.001). The radioactive uptake of the heart (blood), liver, and kidney gradually decreased within the time tested

The mice were intravenously injected with 5.55–7.4 MBq of [^89^Zr]Zr-DFO-NY003 via the tail vein, and the Micro-PET scans were performed at 7, 24, 48, 72, 96, and 144 h p.i.. The representative Micro-PET images of tumor-bearing mice were summarized in Fig. 7.

ImmunoPET images of MDA-MB-231 tumor-bearing mice exhibited substantial high radioactivity accumulation of [^89^Zr]Zr-DFO-NY003 in the tumor, and the uptake in the tumor gradually accumulated with time and could retain for a relatively long period (the maximum tumor uptake value %IA/mL increased from 12.28 ± 2.03 at 24 h p.i. to 25.98 ± 3.77 at 144 h p.i.). Significant uptake could also be observed in some normal organs, such as the heart, liver, spleen, and kidney. Along with the excretion of [^89^Zr]Zr-DFO-NY003 from normal organs mainly through the hepatobiliary pathway, the uptake in normal organs decreased over time, and the tumor-background ratio gradually increased. After the injection of non-targeted antibody [^89^Zr]Zr-DFO-IgG into MDA-MB-231 tumor-bearing mice, the tumor could hardly be delineated in the PET images, and the uptake value was basically consistent with or slightly higher than the blood pool, significantly lower than that of [^89^Zr]Zr-DFO-NY003 (2.88 ± 0.91 at 48 h p.i., *P* < 0.01). Besides, the uptake of [^89^Zr]Zr-DFO-NY003 in the tumor could be significantly blocked by co-injection with cold NY003 (2.23 ± 0.59 at 48 h p.i., *P* < 0.01). Results of non-targeted antibody imaging and blocking study together confirm the uptake of [^89^Zr]Zr-DFO-NY003 in MDA-MB-231 tumors was TROP-2 specific.

### *[*^*177*^*Lu]Lu-DTPA-NY003 SPECT imaging in TNBC models*

**Fig. 8:**
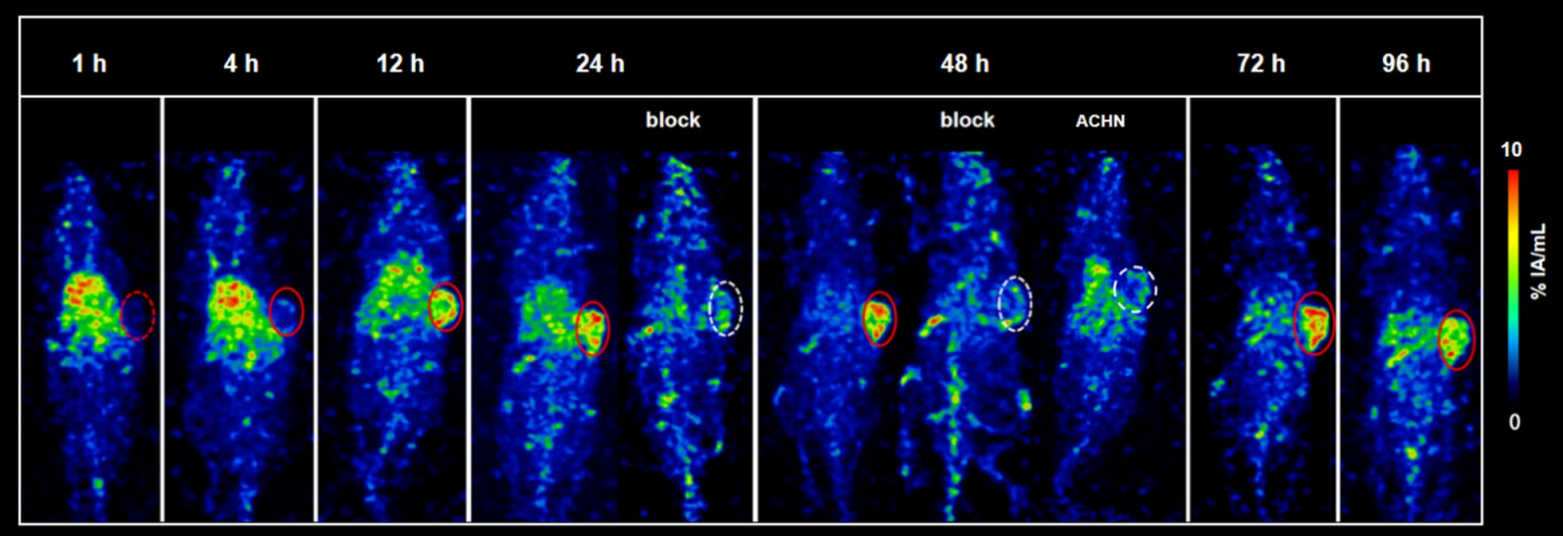
SPECT images in MDA-MB-231 and ACHN tumor-bearing mice at different time points p.i.. SPECT images showed specific high [^177^Lu]Lu-DTPA-NY003 accumulation in tumors which can be blocked by cold NY003

SPECT images of MDA-MB-231 tumor-bearing mice at different time points p.i. were shown in Fig. 8, substantial high radioactivity accumulation of [^177^Lu]Lu-DTPA-NY003 in the tumor, and the uptake in the tumor gradually accumulated with time and could retain for a relatively long period (the maximum tumor uptake value %IA/mL was 3.72 ± 2.19 at 12 h p.i. to 9.31 ± 3.46 at 72 h p.i.). Together with the images of blocking and TROP-2 negative control groups, confirming the high specific uptake in tumors. The radioactivity accumulation of normal organs such as the heart, liver, and spleen was relatively high and gradually decreased over time.

### Ex vivo biodistribution of [^89^Zr]Zr-DFO-NY003/[^177^Lu]Lu-DTPA-NY003 in NSG mice

**Fig. 9:**
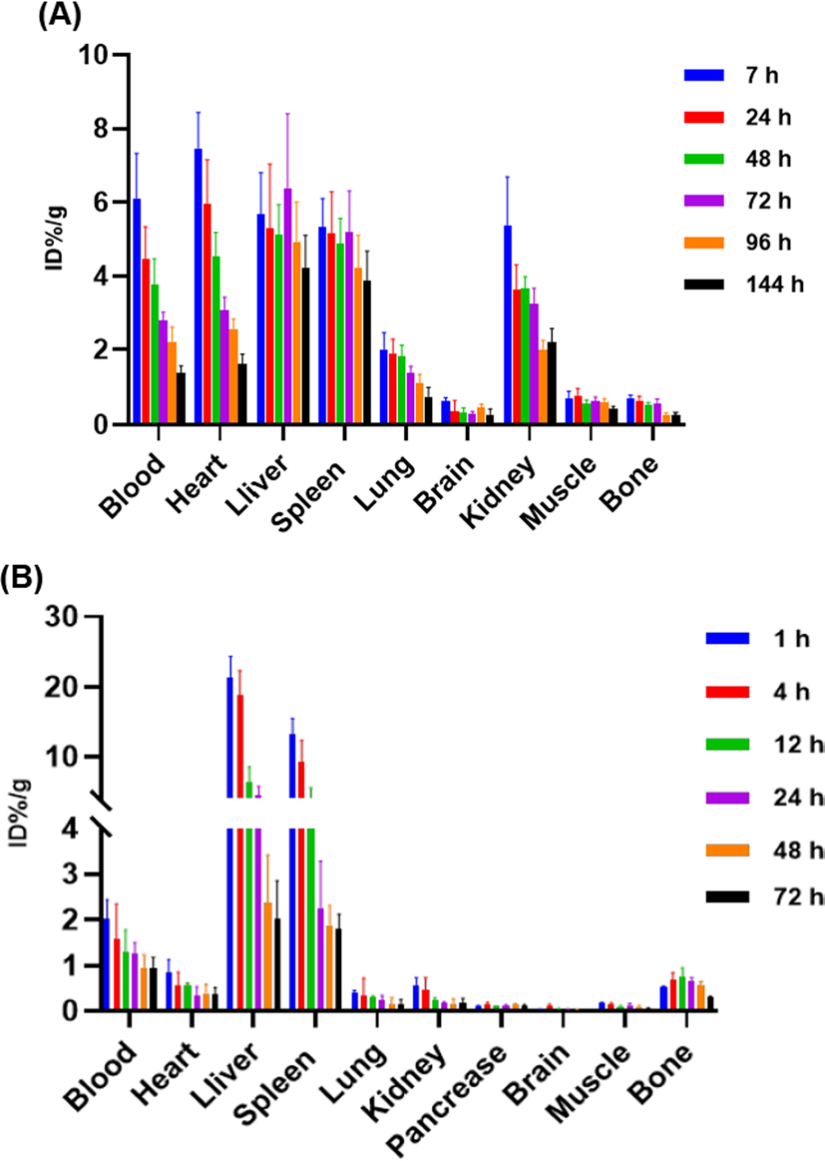
Uptake values for ex vivo tissues of [^89^Zr]Zr-DFO-NY003/[^177^Lu]Lu-DTPA-NY003 in normal NSG mice

We further analyzed the ex vivo biodistribution of [^89^Zr]Zr-DFO-NY003 and [^177^Lu]Lu-DTPA-NY003 in normal NSG mice, respectively. The mice were euthanized and sacrificed for ex vivo biodistribution study at different time points p.i.. As shown in Fig. 9 and Additional file 1: Table S1-2, the ex vivo biodistribution trends were consistent with in vivo imaging results acquired from immunoPET and SPECT imaging, with the heart, blood, liver, and spleen being the high uptake organs. Both [^89^Zr]Zr-DFO-NY003 and [^177^Lu]Lu-DTPA-NY003 were mainly excreted from the hepatobiliary pathway, the highest uptake was observed in the liver, with uptake values of 5.68 ± 1.12 ID%/g at 7 h and 4.21 ± 0.89 ID%/g at 144 h for [^89^Zr]Zr-DFO-NY003 and 21.33 ± 3.04 ID%/g at 1 h and 2.01 ± 0.85 ID%/g at 72 h for [^177^Lu]Lu-DTPA-NY003. Note the uptake in spleen, which was lower than liver, but also higher than other normal organs (5.32 ± 0.78 ID%/g at 7 h and 3.87 ± 0.81 ID%/g at 144 h for [^89^Zr]Zr-DFO-NY003 and 13.29 ± 2.21 ID%/g at 1 h and 1.79 ± 0.32 ID%/g at 72 h for [^177^Lu]Lu-DTPA-NY003). While the radioactivity accumulation in other normal organs was low and decreased with time p.i..

### Efficacy evaluation of [^177^Lu]Lu-DTPA-NY003 in TNBC models

**Fig. 10:**
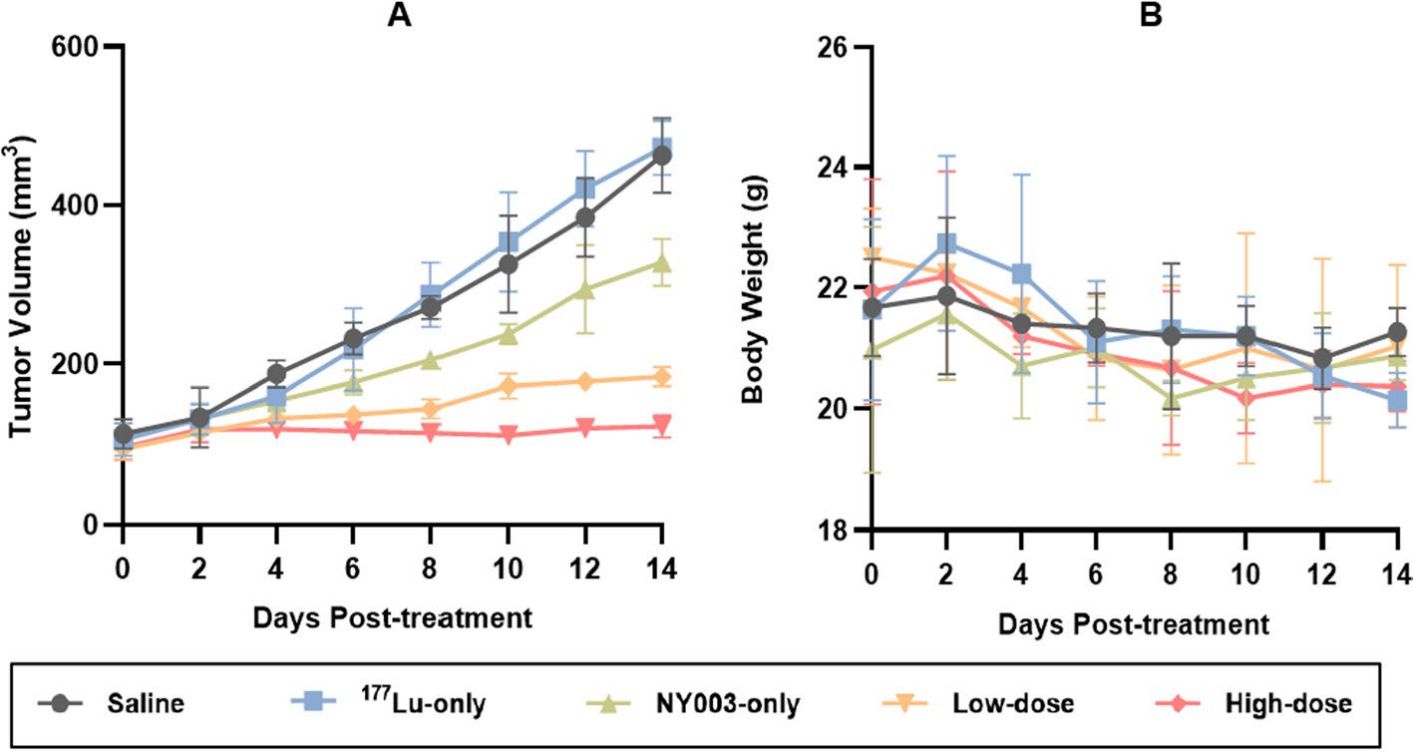
Tumor size and body weight monitoring of RIT studies. **A** The strongest anti-tumor efficacy was observed in the high-dose group, followed by the low-dose group. **B** The body weight of each group showed a downward trend

To evaluate the anti-tumor efficacy of [^177^Lu]Lu-DTPA-NY003, a pilot experiment was conducted using five groups of the TROP-2-overexpressing MDA-MB-231 tumor-bearing mice. The mice in the low-dose and high-dose groups were injected with [^177^Lu]Lu-DTPA-NY003 of 11.1 or 18.5 MBq (200 μL), and the three control groups were injected with ^177^LuCl_3_ solution, non-labeled NY003 antibody, or saline, respectively. Tumor volume and body weight for each group were measured and the results were shown in Fig. 10.

A downward trend was observed in the mice weight of experimental groups after the administration of radiopharmaceuticals within a few days, and then remained relatively stable or even increased (low-dose group). At 14 days p.i., there was no significant difference between the experimental groups and the control groups (*P* > 0.05).

The high-dose group exhibited the strongest anti-tumor efficacy with a tumor volume of 122.30 ± 13.99 mm^3^ at 14 d p.i., which was significantly lower than all three control groups (*P* < 0.05), and also the low-dose group (184.27 ± 12.00 mm^3^, *P* < 0.05). The tumor suppression effect was also observed in the NY003-only group, with a tumor volume of 328.30 ± 29.45 mm^3^, but the efficacy was significantly weaker than the experimental groups described before (*P* < 0.01). There was no statistically significant difference in tumor volume between the ^177^Lu-only and saline groups at 14 d p.i. (saline = 462.70 ± 47.10 mm^3^, ^177^Lu-only = 472.20 ± 33.82 mm^3^, *P* > 0.05).

### Safety evaluation and histological staining

**Fig. 11:**
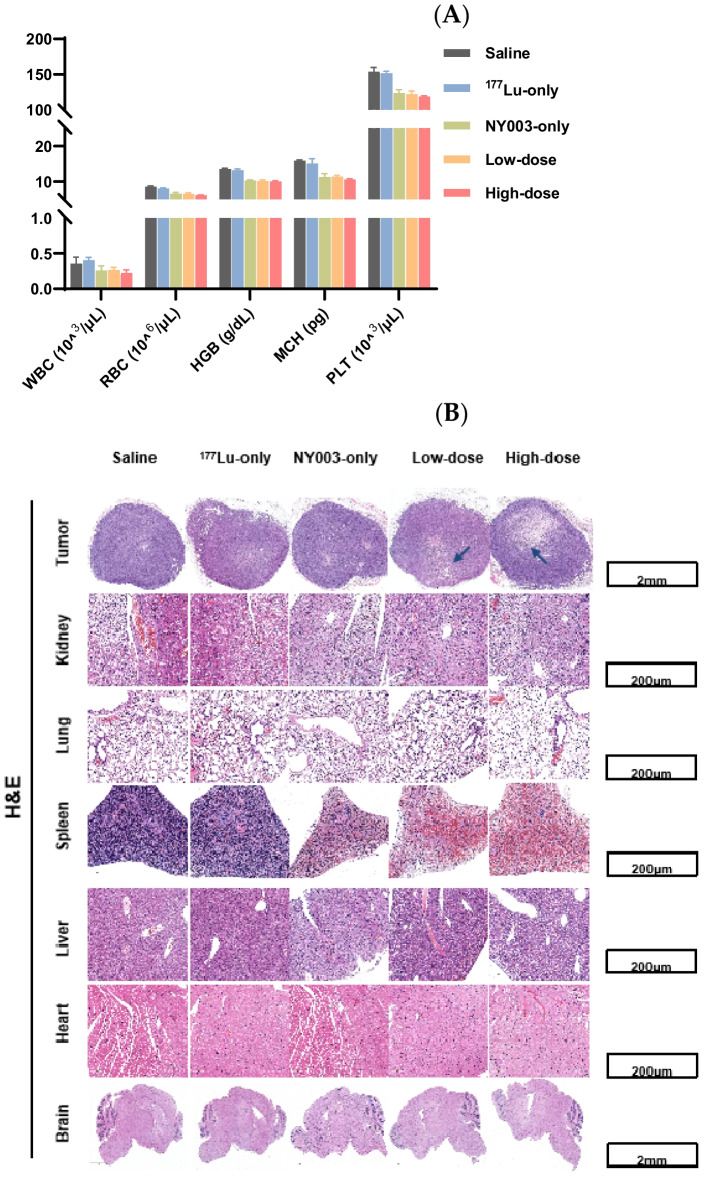
Hematological analysis and H&E staining. **A** Hematological analysis showed that the WBC, RBC, HGB, MCH, and PLT counts of low- and high-dose and NY003-only groups decreased within the observation period (14 d p.i.). **B** The H&E staining results showed regional or focal necrosis within the tumors in low- and high-dose groups, especially the high-dose group, illustrating the therapeutic effect of [^177^Lu]Lu-DTPA-NY003. Hepatic congestion and splenic white pulp atrophy were observed in low- and high-dose and NY003-only groups

There was no death within the observation period in either the experimental or control group, and there was no significant difference in diet, excretion, activity, mental state, and skin condition. No significant difference was observed in the size, color, shape, and texture of the organs among the six groups. The blood routine indexes of mice in experimental (low- and high-dose) and NY003-only groups decreased and were lower than those of saline and ^177^Lu-only groups at the 14 days p.i. (Fig. 11A). The RBC count in ^177^Lu-only, NY003-only, low-dose and high-dose groups was significantly lower than that of saline group (*P* < 0.05). The levels of HGB, MCH and PLT in NY003-only, low-dose and high-dose groups were significantly lower than that of saline group (*P* < 0.05).

The H&E staining was performed in tumors and major organs (the original images were provided in the supplementary material). The H&E staining results showed regional or focal necrosis within the tumors in low- and high-dose groups, especially the high-dose group, illustrating the therapeutic effect of [^177^Lu]Lu-DTPA-NY003 (Fig. 11B). Hepatic congestion and splenic white pulp atrophy were observed in the low-dose, high-dose and NY003-only groups, illustrating some toxic effects after RIT treatment which may mainly be caused by the anti-TROP-2 antibody.

## Discussion

Triple-negative breast cancer is one of the most lethal human cancers characterized by the high aggressiveness and heterogeneity and absence of the expression of estrogen receptor (ER), progesterone receptor (PR), and human epidermal growth factor receptor 2 (HER2). The standard therapeutic regimens currently followed could not kill the tumor effectively and prolong patient survival significantly. Therefore, searching for novel treatment options is urgent and has become a hot topic in research.

Human trophoblast cell surface glycoprotein antigen (TROP-2), also known as tumor-associated calcium signal transducer 2 (TACSTD2), is the protein product of the TACSTD2 gene, a type I cell surface glycoprotein highly expressed in human cancers (Cubas et al. 2009). The tumor-specific over-expression of TROP-2 in various cancer cells plays a crucial role in regulating the self-renewal, proliferation, and transformation of tumors (Stoyanova et al. 2012). In addition, the degree of tumor progression correlates with the expression level of TROP-2. Based on the aforementioned, TROP-2 could serve as an ideal target which opens up possibilities for targeted diagnosis and treatment. In 2020, the first TROP-2-targeted ADC Trodelvy (sacituzumab govitecan, IMMU-132) was granted accelerated approval by the FDA for the treatment of advanced triple-negative breast cancer. SG consists of a humanized κ monoclonal anti-TROP-2 antibody (hRS7), a maleimide-polyethylene glycol-acid-sensitive cleavable carbonate linker, and an irinotecan metabolite (SN-38). The hRS7 antibody could bind directly to TROP-2-expressing tumor cells and trigger the internalization of the SGs, leading to hydrolysis of the CL2A linker and release of the topoisomerase I inhibitor, SN-38, thereby inducing DNA damage and ultimately apoptosis. In addition, in an acidic environment, pH-dependent linkers are hydrolyzed around the tumor and the release of SN-38 kills surrounding tumor cells through a bystander effect (Bardia et al. 2021; Liu et al. 2022; Okajima et al. 2021). Besides SG, more than twenty TROP-2-targeted novel ADCs have been developed by different companies and are undergoing preclinical and clinical trials. The precise quantification of the expression level of TROP-2 is critical for screening certain patients who may benefit from the TROP-2 ADC treatment. ImmunoPET imaging allows for the screening and quantification of TROP-2 expression level in vivo non-invasively and sensitively, making it a worthwhile step prior to SG administration.

Among various radioisotopes used for antibody radiolabelling, ^89^Zr is one of the most ideal choices because of its relatively long physical half-life (t_1/2_ = 78.4 h), which is a good match for antibody pharmacokinetics. Moreover, the high γ-energy and good spatial resolution of ^89^Zr result in high-resolution and excellent-quality PET images. In recent years, ^89^Zr has been widely used in antibody radiolabelling and PET imaging studies (Li et al. 2018).

In the present study, we investigated, the development, quality control, and ex/in vivo animal studies of the ^89^Zr radiolabelling anti-TROP-2 antibody NY003 to validate its feasibility for non-invasive immunoPET imaging of TROP-2 over-expression tumors. The results demonstrate that [^89^Zr]Zr-DFO-NY003 could clearly depict the morphology of TROP-2 positive MDA-MB-231 xenograft at 7 h p.i. with the uptake value of 8.19 ± 1.11%ID/g (*n* = 3), and the accumulation of radiotracer in tumor increased significantly over time, reaching 12.28 ± 2.03% ID/g at 24 h p.i., which was significantly higher than that of [^89^Zr]Zr-DFO-IgG and NY003-blocked groups (*P* < 0.01). At the last time point of the Micro-PET imaging study (144 h p.i.), the uptake value of [^89^Zr]Zr-DFO-NY003 was 25.98 ± 2.77%IA/mL, approximately 17 times the uptake of the heart (blood pool), indicating the specific tumor uptake and providing the excellent image contrast. Although the tumor-to-background ratio continued to increase over time, the tumor could already be separated clearly from the background at 24 h p.i.. The biodistribution data and IHC results were consistent with the Micro-PET images, robustly supporting that the TROP-2 targeted immunoPET could be a useful tool for the TROP-2 (+) tumor detection and the TROP-2 expression level e evaluation. Other investigators have also used different radiolabeled TROP-2 antibodies to visualize various tumors. The anti-human TROP-2 antibody AF650 and hIMB1636 were radiolabeled with ^89^Zr and ^64^Cu respectively for the detection of pancreatic cancer in tumor-bearing mice, and the tumors could be clearly visualized in PET images as expected (Chen et al. 2022; Li et al. 2022). Besides, a ^64^Cu radiolabelling TROP-2 antibody was used in a study of prostate cancer diagnosis, and a specific high accumulation was observed in TROP-2 (+) PCa tumors (Sperger et al. 2023). These studies demonstrate that anti-TROP-2 specific immunoPET imaging can facilitate the diagnosis of various TROP-2 (+) tumors and screening of the potent beneficiaries of anti-TROP-2 ADC therapy. However, our study investigated, for what is to our knowledge the first time, the preclinical validation of anti-TROP-2 antibody for non-invasive PET imaging of TNBC diagnosis and organ biodistribution in animal models.

Radionuclide Drug Conjugate (RDC) is designed and developed by coupling a precision-targeted molecule (monoclonal antibody or peptide/small molecule, ligand) with a potent killing factor (radioisotope) using a linker. The anti-tumor mechanism of RDC is similar to that of ADC, the antibodies or targeted small molecules were used to mediate specific targeting, delivering imaging or therapeutic radionuclides to the certain target, thereby concentrating the radiation produced by the radioisotope only on the targeted lesion, providing highly precise and effective treatment. ^177^Lu is currently the most commonly used radionuclide for RDC, emits β-particles with three energies of 497 keV (78.6%), 384 keV (9.1%), and 176 keV (12.2%), and has a half-life of nearly one week, making it particularly suitable for the therapy of tumors and metastases. Meanwhile, the γ-rays emitted from ^177^Lu could also be used for SPECT imaging to achieve the purpose of theranostics.

In this study, we used [^177^Lu]Lu-DTPA-NY003 for RIT of the subcutaneous TROP-2 (+) TNBC tumor model (MDA-MB-231). The tumor growth in the high-dose-[^177^Lu]Lu-DTPA-NY003 group (18.5 MBq) has been suppressed significantly and almost reached a stable state 4–6 days post-injection (122.30 ± 13.99 mm^3^ at 14 d p.i.). A single administration of the low-dose [^177^Lu]Lu-DTPA-NY003 (11.1 MBq) also exhibited substantial anti-tumor efficacy, the tumor volume of the low-dose group increased within the experimental period, it was also significantly smaller than other control groups (184.27 ± 12.00 mm^3^ at 14 d p.i.). The liver and spleen showed a relatively high accumulation of [^177^Lu]Lu-DTPA-NY003 in SPECT images and biodistribution data, indicating the hepatobiliary excretion of [^177^Lu]Lu-DTPA-NY003 and maybe some non-specific binding with the liver and spleen. The hematological analysis and histological staining results proved that [^177^Lu]Lu-DTPA-NY003 could kill tumor cells while causing minimal toxicity to other healthy tissues and organs. The toxic effects were limited in the liver and spleen, which may mainly be caused by the anti-TROP-2 antibody but not the radionuclide ^177^Lu, since the toxic effects were observed in the NY003-only group but not in the ^177^Lu-only group. A combination of the relatively large molecular weight and excretion pathway of the full-length antibody may contribute to this result.

Hepatic congestion and splenic white pulp atrophy were observed in low- and high-dose and NY003-only groups, illustrating some toxic effects after RIT treatment which may mainly be caused by the anti-TROP-2 antibody.

The different chelators used for ^89^Zr and ^177^Lu radiolabeling may have an impact on the distribution of radiopharmaceuticals. However, all the in vivo results in our study showed that the distribution patterns of [^89^Zr]Zr-DFO-NY003 and [^177^Lu]Lu-DTPA-NY003 were very similar, substantially high radioactivity was found in tumors. The main excretory organ was the liver, and a small portion was excreted through the kidneys. So, despite the application of different chelators, the effect of theranostic will not be hampered.

Further efforts are also needed to optimize the structure of NY003 and improve its pharmacokinetic properties, such as reducing the molecular weight of the antibody while retaining its high affinity, so as to accelerate the circulation of the antibody in vivo, and further improve the target-to-background ratio to achieve a higher image contrast, better therapeutic efficacy and lower toxicity to healthy organs (Sheridan 2017). Moreover, a series of TNBC cell lines with different expression levels of TROP-2 should be used in further studies to analyze whether the uptake values and anti-tumor efficacy were related to the TROP-2 expression levels. Considering the heterogeneity of tumors in TNBC patients, the PDX model may be better than the xenograft tumor model in preclinical and clinical translation research. The observation period should be extended as long as possible in future research to assess the long-term anti-tumor effects and toxicity of [^177^Lu]Lu-DTPA-NY003 as well as the survival of the experimental mice.

## Conclusion

Our study demonstrated that [^89^Zr]Zr-DFO-NY003/[^177^Lu]Lu-DTPA-NY003 could non-invasively detect the expression of TROP-2 in tumors through specific immunoPET and SPECT imaging, which may support beneficiaries' screening and therapeutic response prediction. [^177^Lu]Lu-DTPA-NY003 could suppress tumor growth through RIT in TROP-2 over-expression TNBC models, providing alternative treatment option for those refractory TNBC patients.

### Supplementary Information

**Additional file 1.** Supplementary figures and tables.

## Data Availability

The datasets generated during and/or analysed during the current study are available from the corresponding author on reasonable request.

## Abbreviations

TNBC: Triple negative breast cancer
ADC: Antibody–drug conjugates
TROP-2: Trophoblast cell-surface antigen-2
TLC: Thin-layer chromatography
HPLC: High-performance liquid chromatography
PET: Positron emission tomography
SPECT: Single-photon emission computed tomography
