# Supplementary material for: Preclinical evaluation of the theranostic potential of 89Zr/177Lu-labeled anti-TROP-2 antibody in triple-negative breast cancer model
Source: EJNMMI Radiopharm Chem. 2024 Jan 9;9:5. doi: 10.1186/s41181-023-00235-x (PMC10776551; doi:10.1186/s41181-023-00235-x)
Supplement: Supplementary file 1 — Additional file 1. Supplementary figures and tables. [file 41181_2023_235_MOESM1_ESM.docx]

**Supplementary**

Preclinical Evaluation of the Theranostic Potential of ^89^Zr/^177^Lu-Labeled Anti-TROP-2 Antibody in Triple-Negative Breast Cancer Model

Yitian Wu^1,2^, Tuo li^2^, Xianzhong Zhang^1^, Hongli Jing^2^, Fang Li^2, *^ and Li Huo^2, *^

^1^ Medical Science Research Center, Peking Union Medical College Hospital, Chinese Academy of Medical Sciences, Beijing, 100730, China

2 Department of Nuclear Medicine, State Key Laboratory of Complex Severe and Rare Diseases, Center for Rare Diseases Research Beijing Key Laboratory of Molecular Targeted Diagnosis and Therapy in Nuclear Medicine, Peking Union Medical College Hospital, Chinese Academy of Medical Science and Peking Union Medical College, Beijing, China

* Correspondence: Fang Li: lifang@pumch.cn and Li Huo: huoli@pumch.cn


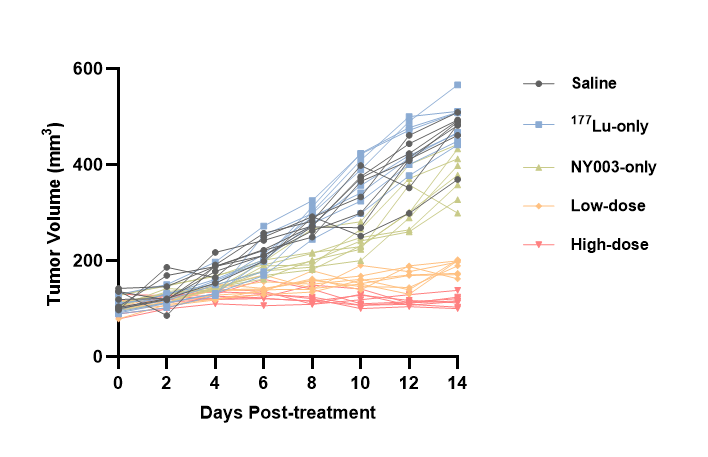


**Figure S1.** Tumor growth curves of individual animals in RIT studies.

**Table S1.** Uptake values (ID%/g) for ex vivo tissues of [^89^Zr]Zr-DFO-NY003 in normal NSG mice.

| **Time post injection (h)** | **7** | **24** | **48** | **72** | **96** | **144** |
| --- | --- | --- | --- | --- | --- | --- |
| **Blood** | 6.11±1.21 | 4.46±0.88 | 3.76±0.71 | 2.81±0.21 | 2.22±0.40 | 1.37±0.19 |
| **Heart** | 7.46±0.98 | 5.97±1.18 | 4.53±0.65 | 3.06±0.37 | 2.55±0.29 | 1.62±0.26 |
| **Liver** | 5.68±1.12 | 5.29±1.75 | 5.12±0.82 | 6.37±2.03 | 4.91±1.09 | 4.21±0.^89^ |
| **Spleen** | 5.32±0.78 | 5.17±1.12 | 4.87±0.69 | 5.20±1.11 | 4.24±0.86 | 3.87±0.81 |
| **Lung** | 2.01±0.45 | 1.91±0.38 | 1.82±0.31 | 1.36±0.19 | 1.11±0.23 | 0.76±0.25 |
| **Brain** | 0.63±0.11 | 0.37±0.29 | 0.34±0.12 | 0.31±0.05 | 0.47±0.09 | 0.28±0.15 |
| **Kidney** | 5.35±1.34 | 3.63±0.67 | 3.65±0.33 | 3.26±0.41 | 2.00±0.25 | 2.21±0.37 |
| **Muscle** | 0.73±0.18 | 0.79±0.20 | 0.59±0.09 | 0.65±0.10 | 0.61±0.10 | 0.43±0.07 |
| **Bone** | 0.70±0.10 | 0.66±0.12 | 0.53±0.07 | 0.59±0.11 | 0.26±0.07 | 0.27±0.07 |

**Table S2.** Uptake values (ID%/g) for ex vivo tissues of [^177^Lu]Lu-DTPA-NY003 in normal NSG mice.

| **Time post injection (h)** | **1** | **4** | **12** | **24** | **48** | **72** |
| --- | --- | --- | --- | --- | --- | --- |
| **Blood** | 2.01±0.43 | 1.56±0.78 | 1.28±0.50 | 1.25±0.25 | 0.96±0.28 | 0.93±0.25 |
| **Heart** | 0.84±0.28 | 0.55±0.30 | 0.55±0.05 | 0.34±0.19 | 0.36±0.22 | 0.37±0.15 |
| **Liver** | 21.33±3.04 | 18.78±3.57 | 6.41±2.14 | 4.54±1.26 | 2.36±1.05 | 2.01±0.85 |
| **Spleen** | 13.29±2.21 | 9.32±3.09 | 4.20±1.43 | 2.26±1.03 | 1.86±0.45 | 1.79±0.33 |
| **Lung** | 0.38±0.06 | 0.32±0.40 | 0.30±0.03 | 0.24±0.09 | 0.15±0.13 | 0.14±0.11 |
| **Kidney** | 0.57±016 | 0.46±0.27 | 0.24±0.04 | 0.18±0.02 | 0.15±0.11 | 0.17±0.10 |
| **Pancreas** | 0.11±0.01 | 0.13±0.55 | 0.09±0.00 | 0.11±0.03 | 0.15±0.01 | 0.11±0.03 |
| **Brain** | 0.04±0.01 | 0.12±0.03 | 0.05±0.01 | 0.03±0.01 | 0.02±0.01 | 0.02±0.01 |
| **Muscle** | 0.16±0.02 | 0.14±0.03 | 0.09±0.02 | 0.12±0.05 | 0.09±0.03 | 0.06±0.01 |
| **Bone** | 0.51±0.03 | 0.70±0.14 | 0.74±0.2 | 0.67±0.07 | 0.57±0.07 | 0.29±0.03 |


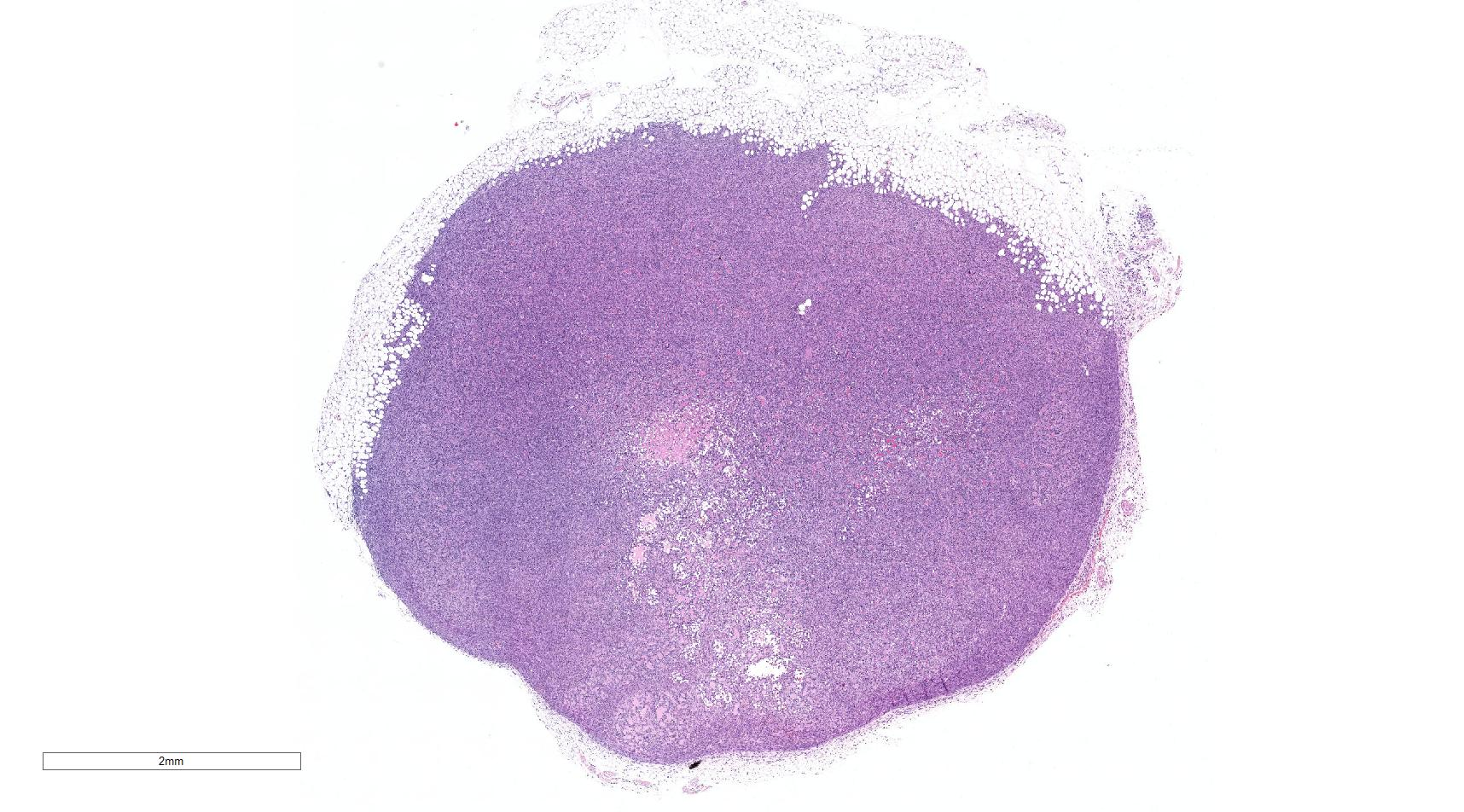


**Figure S2.** The H&E staining result of tumor in low-dose group.


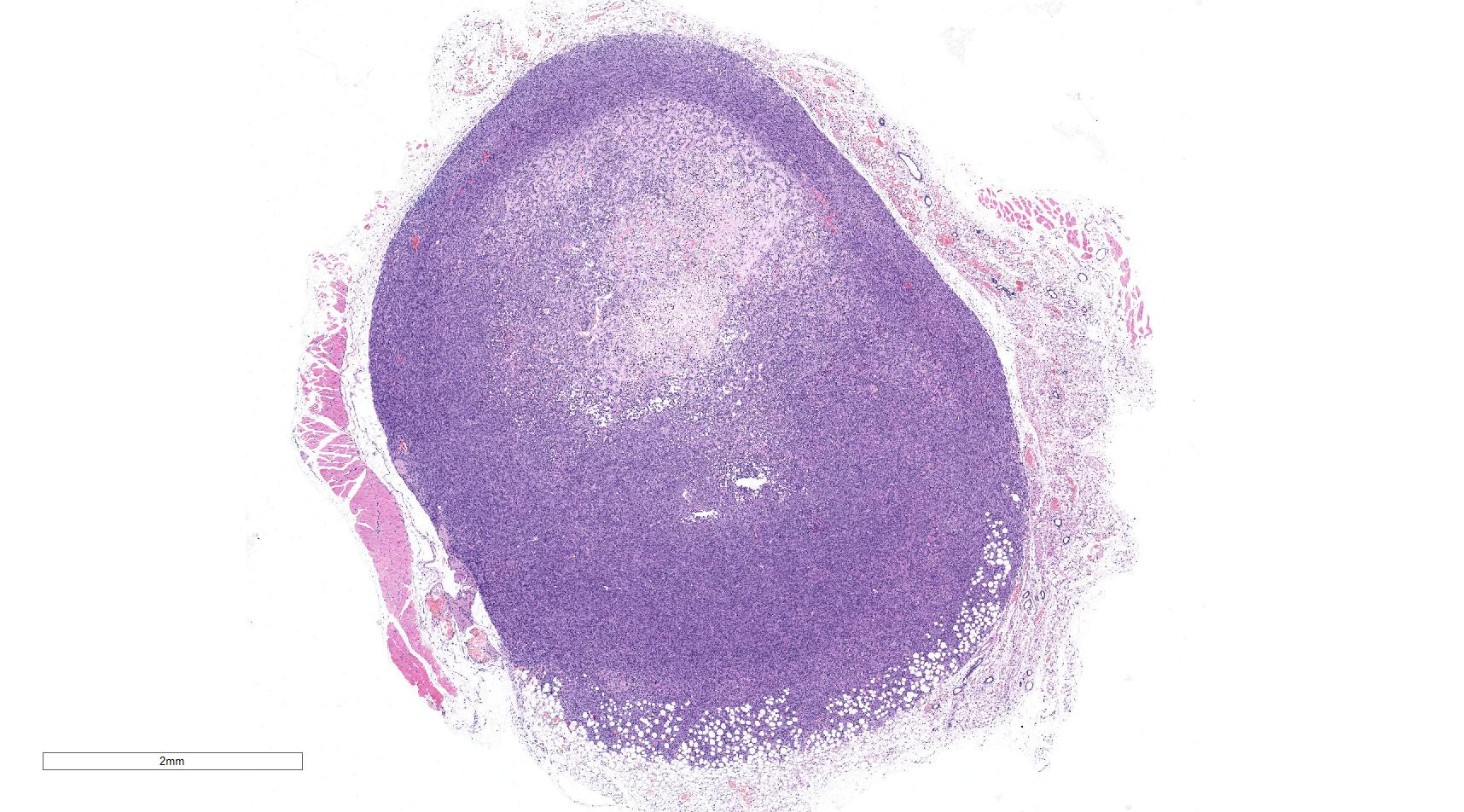


**Figure S3.** The H&E staining result of tumor in high-dose group.


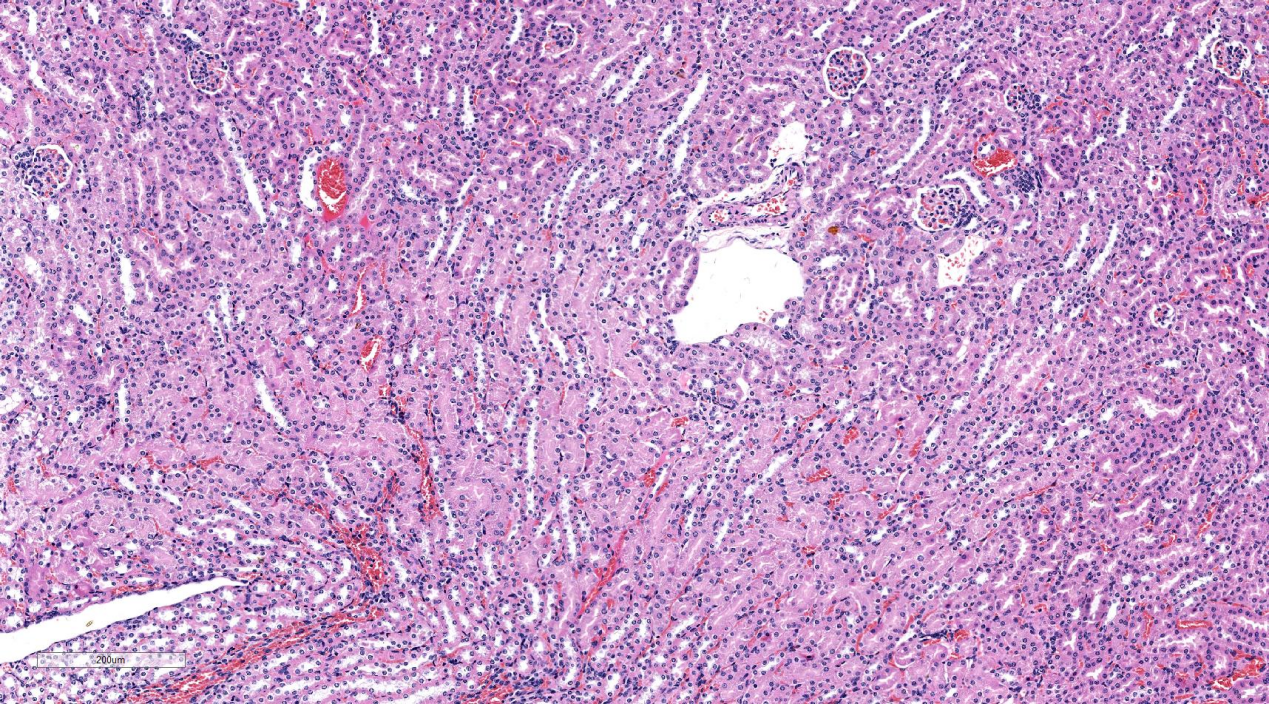


**Figure S4.** The H&E staining result of kidney in low-dose group.


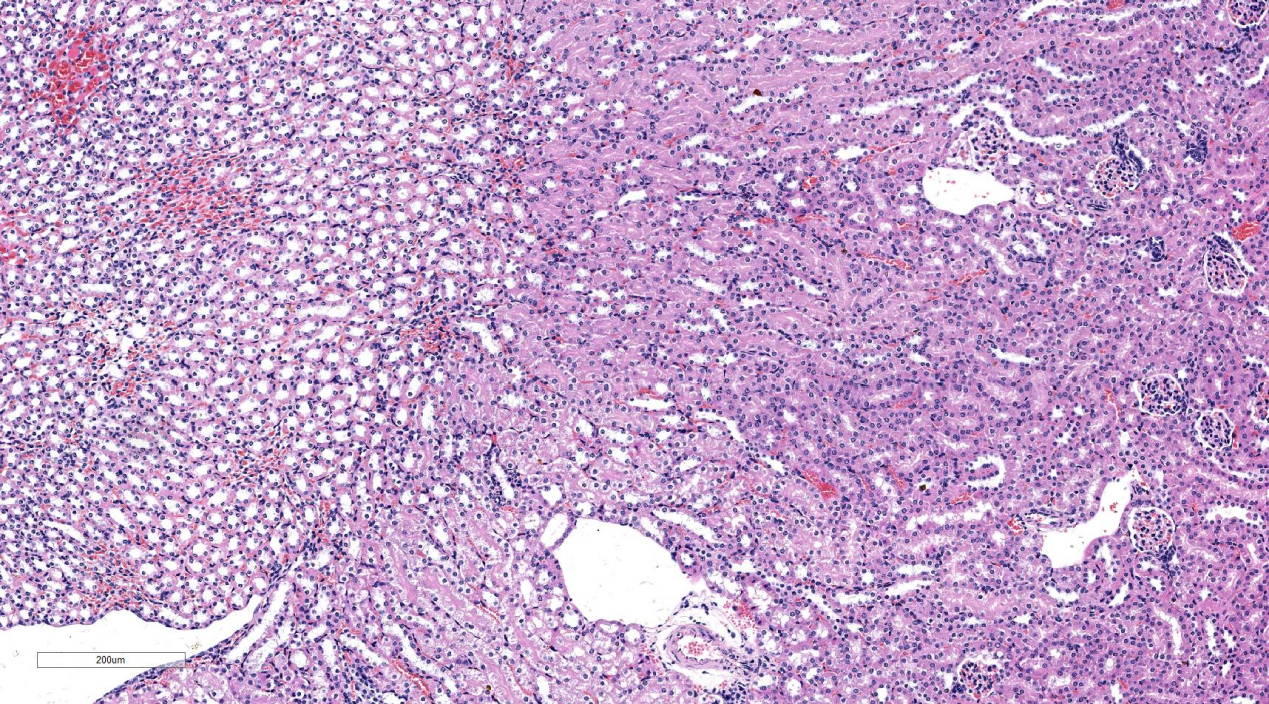


**Figure S5.** The H&E staining result of kidney in high-dose group.


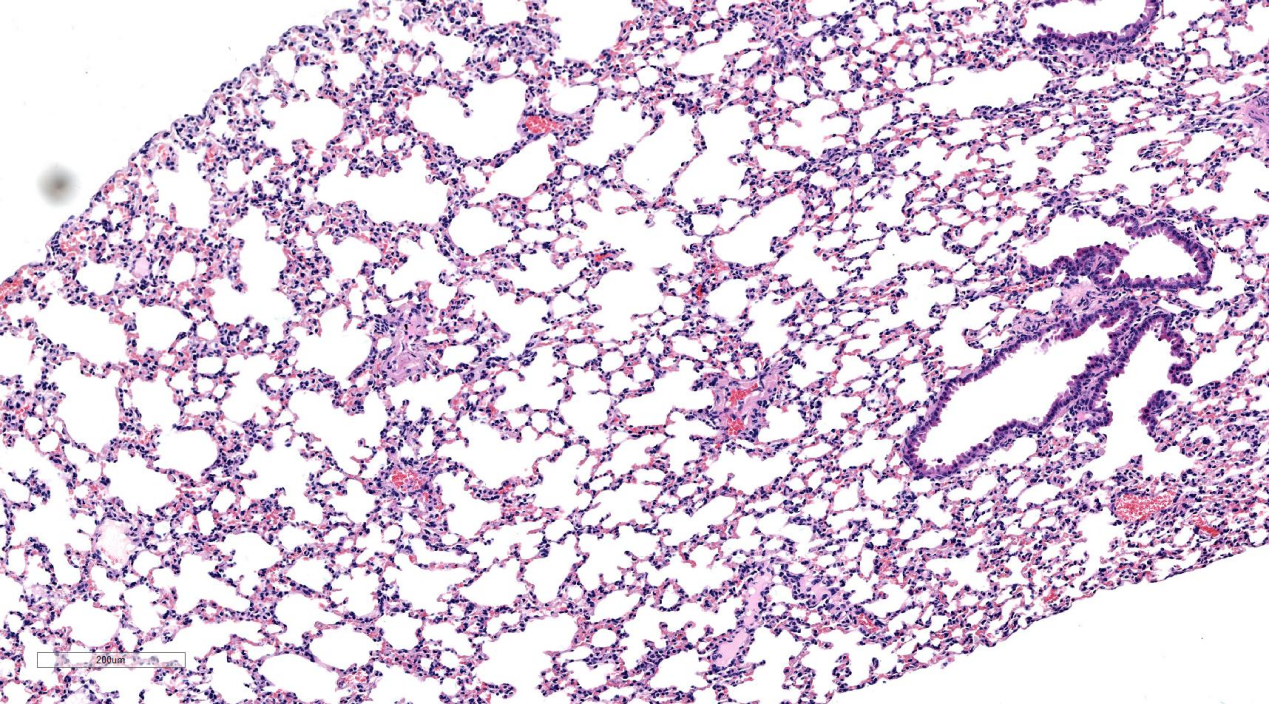


**Figure S6.** The H&E staining result of lung in low-dose group.


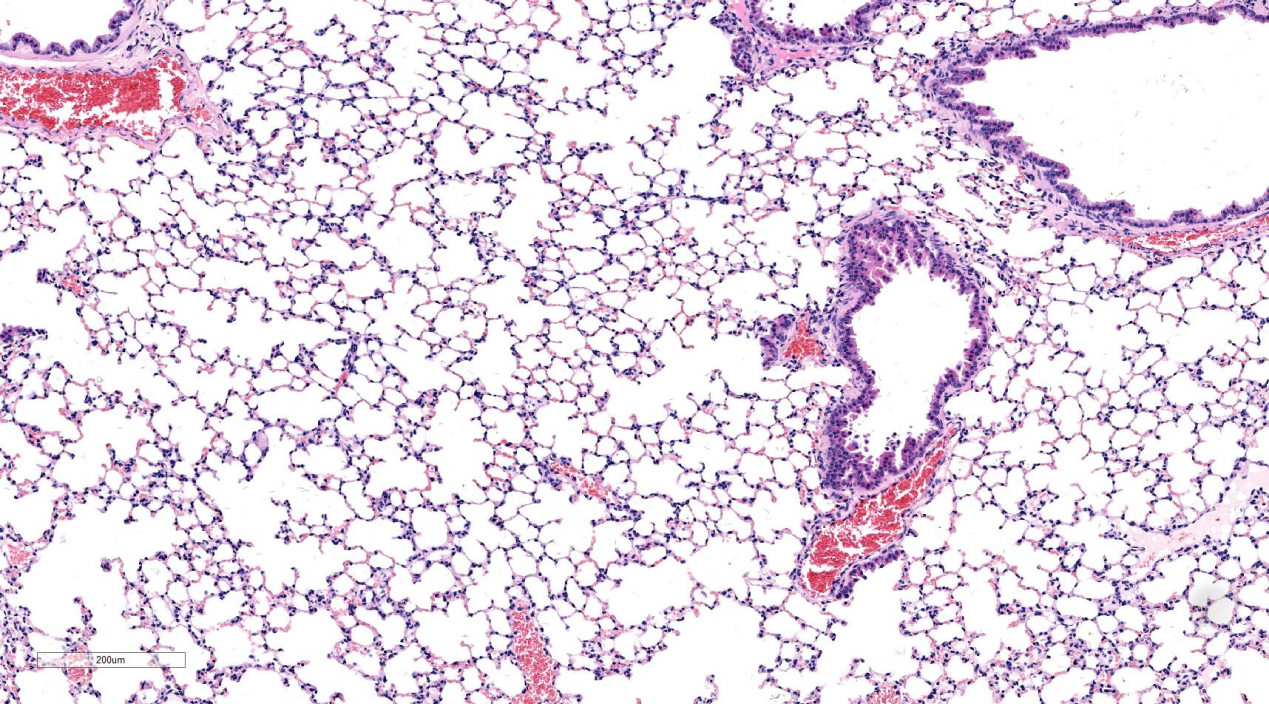


**Figure S7.** The H&E staining result of lung in high-dose group


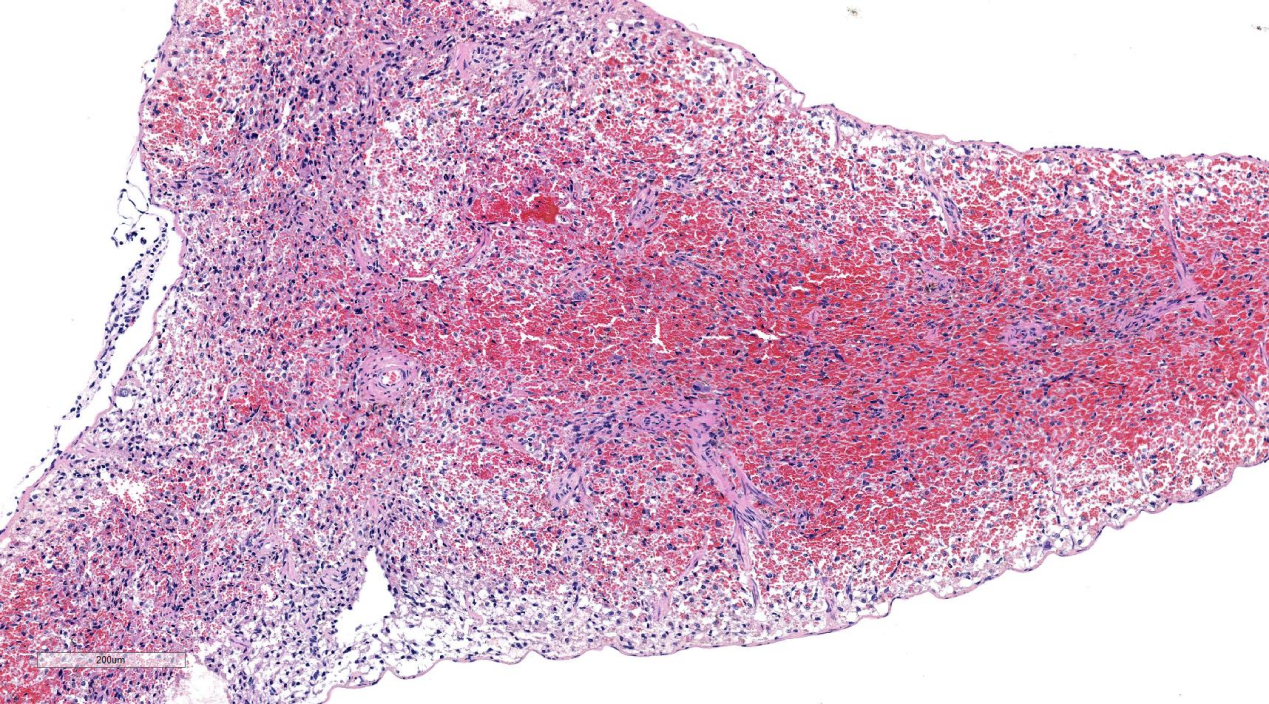


**Figure S8.** The H&E staining result of spleen in low-dose group.


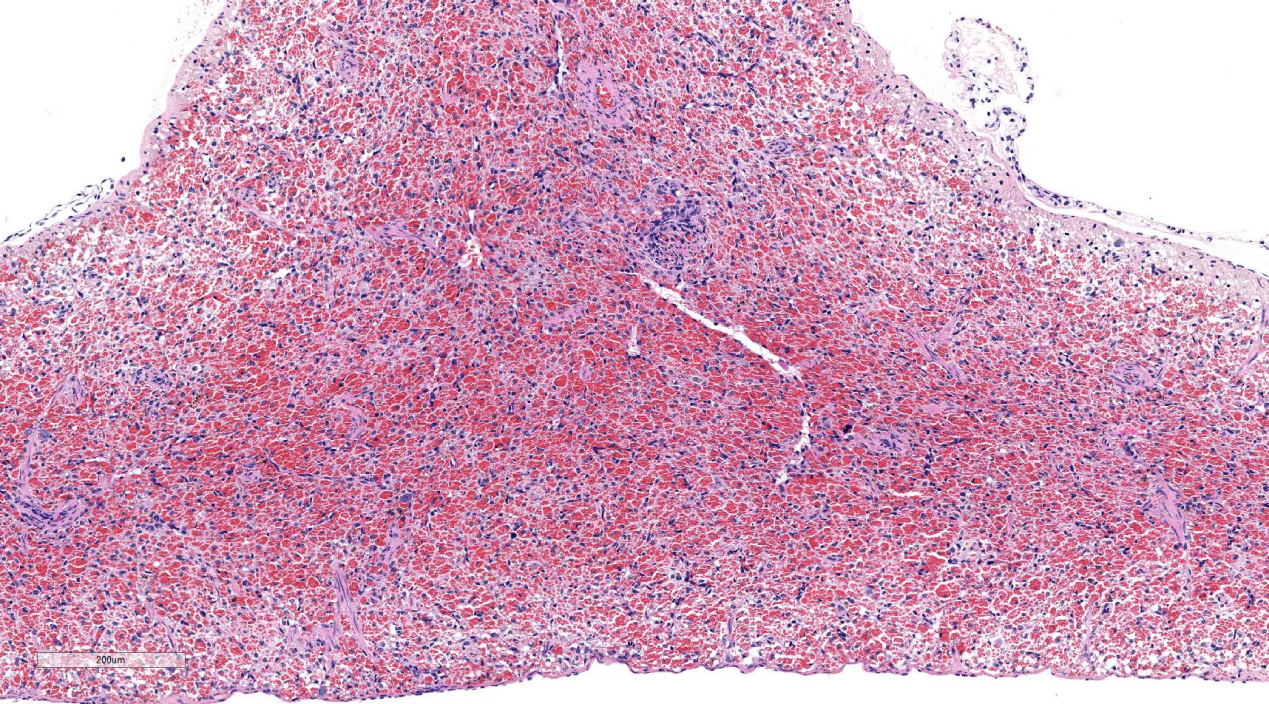


**Figure S9.** The H&E staining result of spleen in high-dose group.


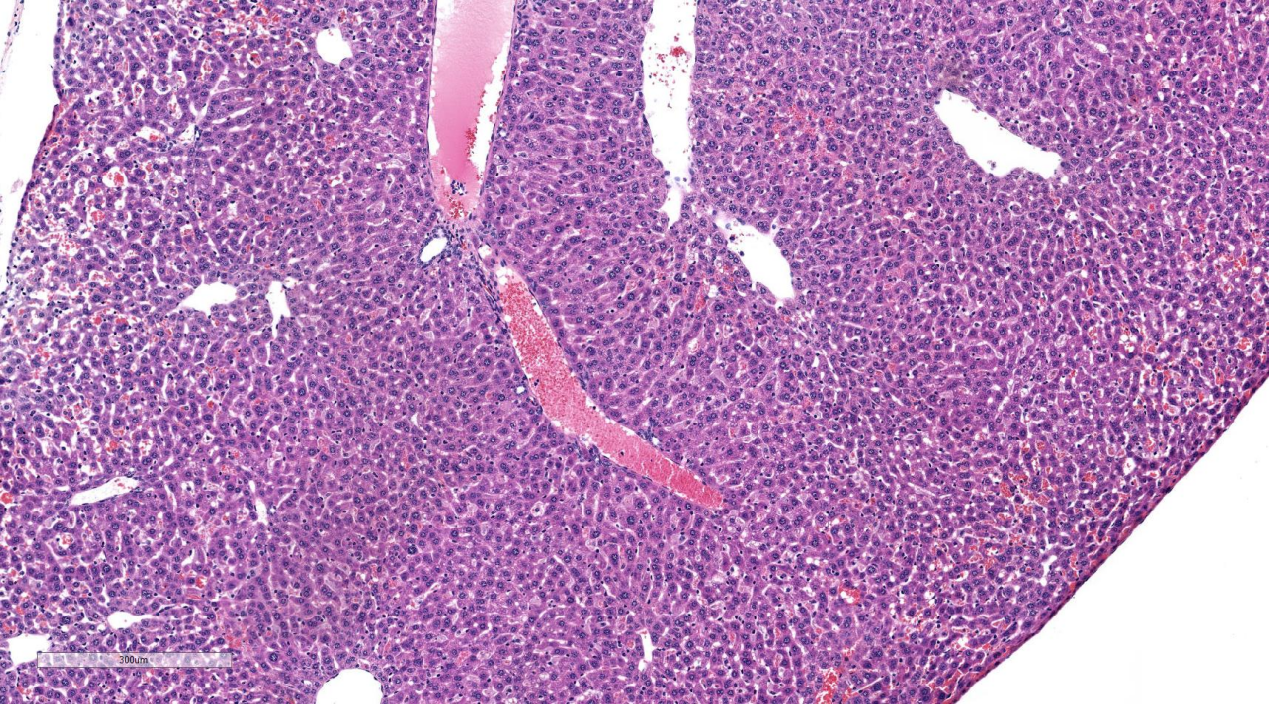


**Figure S10.** The H&E staining result of liver in low-dose group.


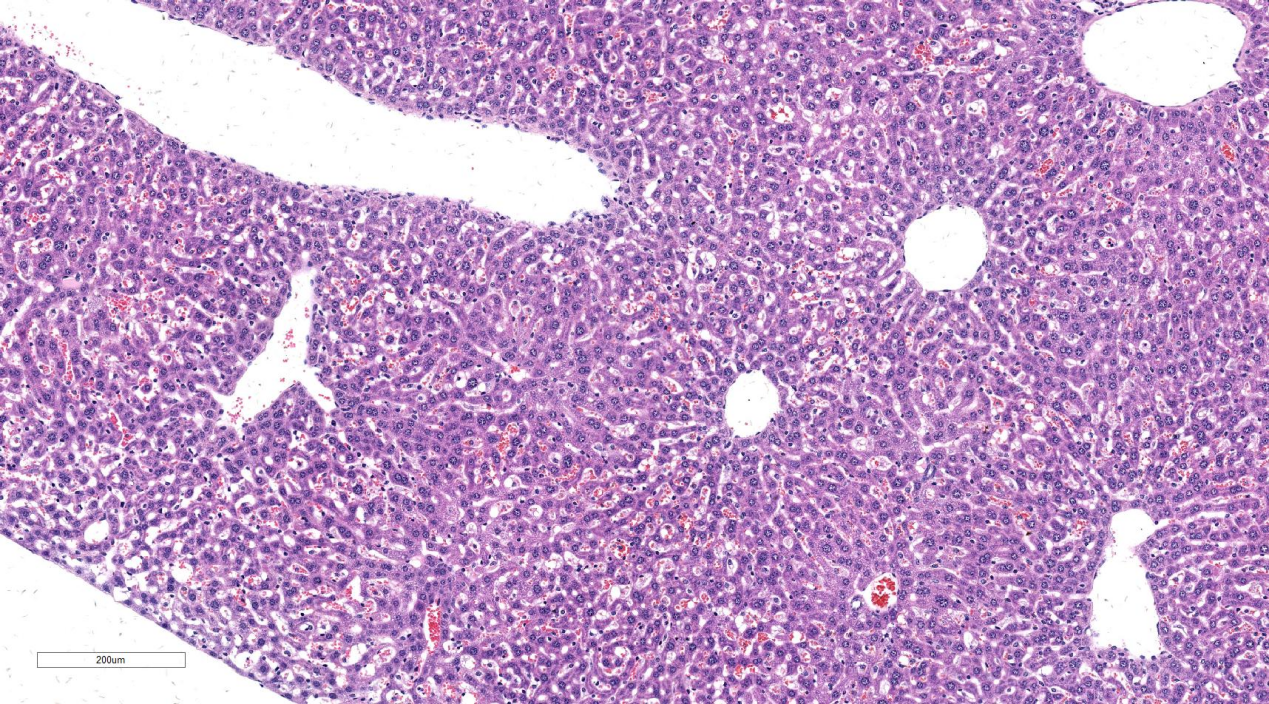


**Figure S11.** The H&E staining result of liver in high-dose group.


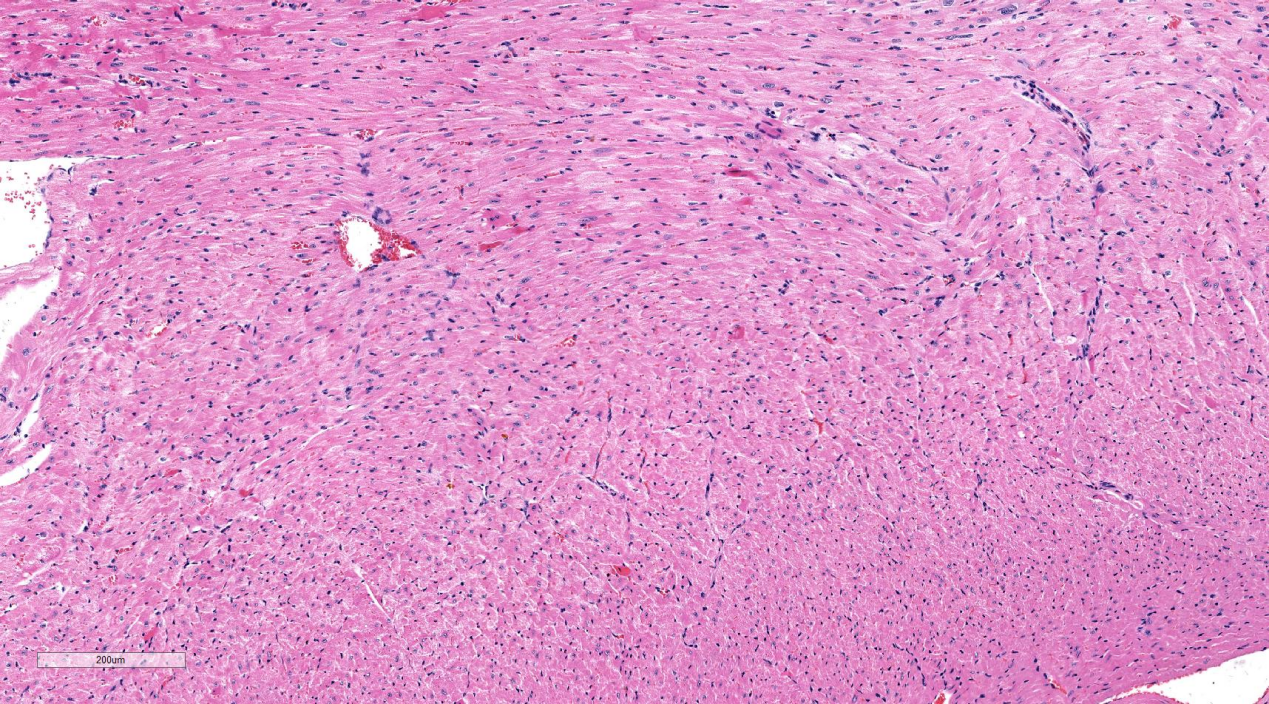


**Figure S12.** The H&E staining result of heart in low-dose group.


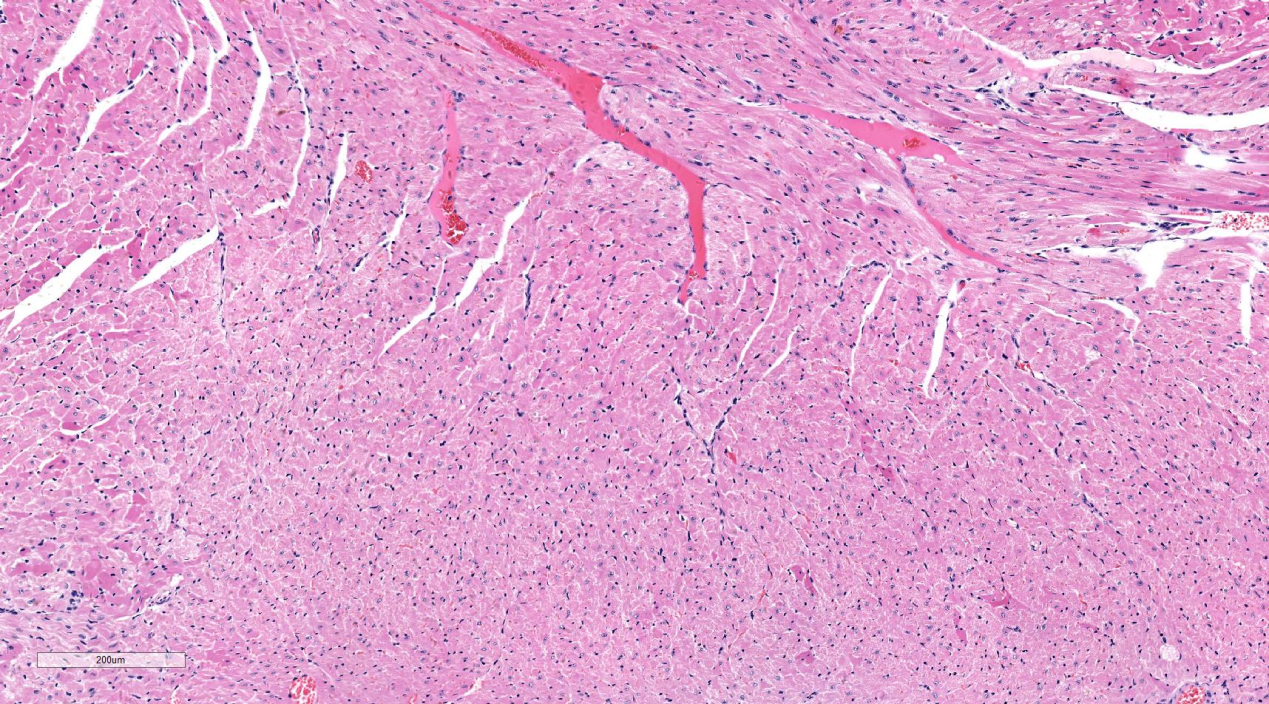


**Figure S13.** The H&E staining result of heart in high-dose group.


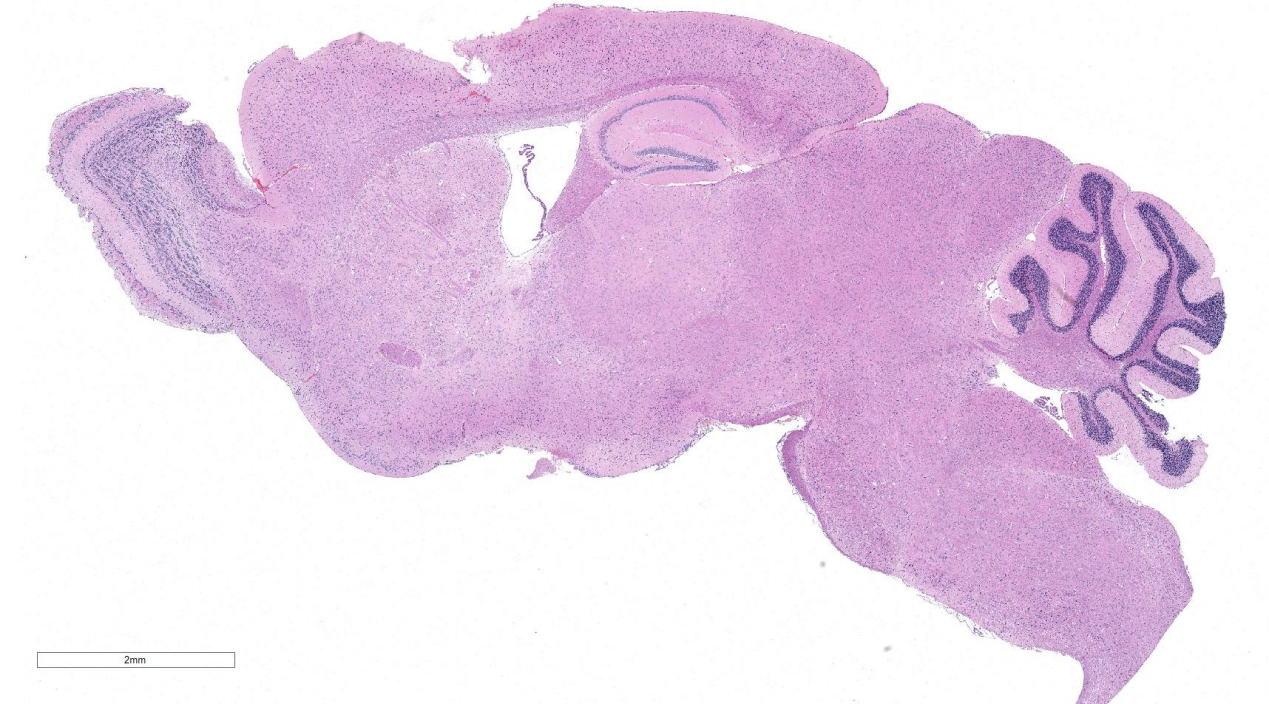
**Figure S14.** The H&E staining result of brain in low-dose group.


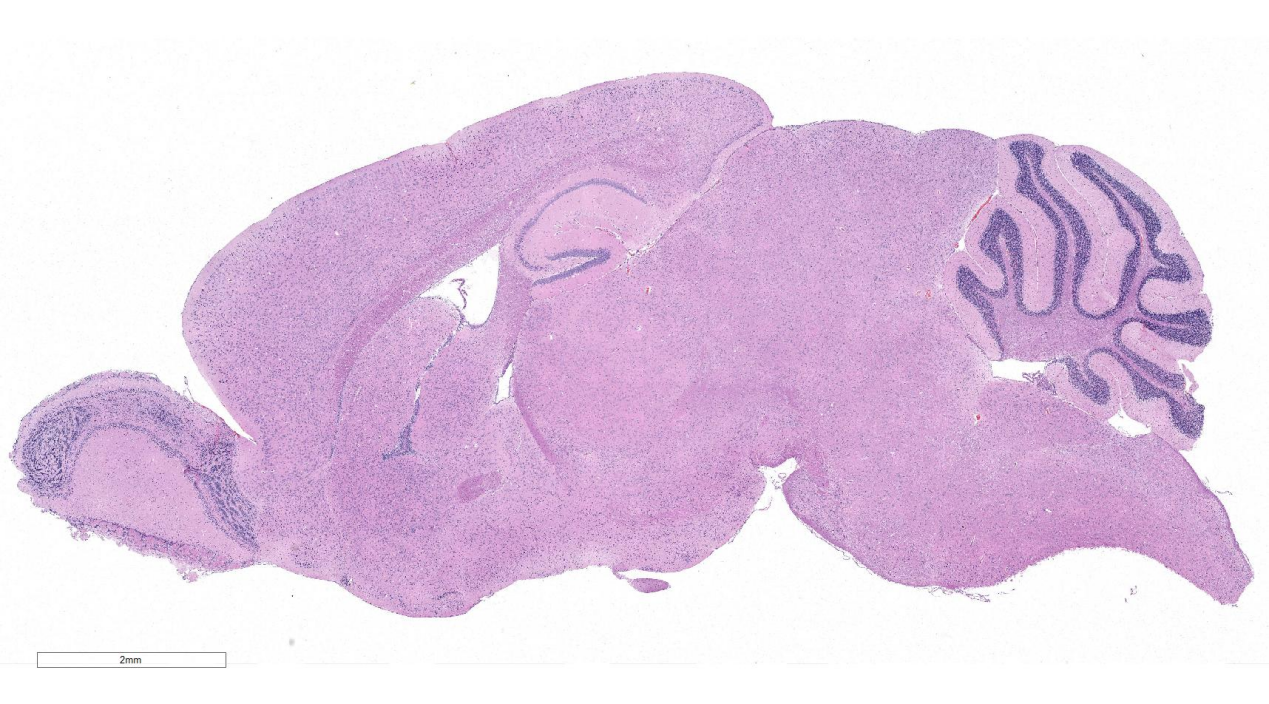


**Figure S15.** The H&E staining result of brain in high-dose group.


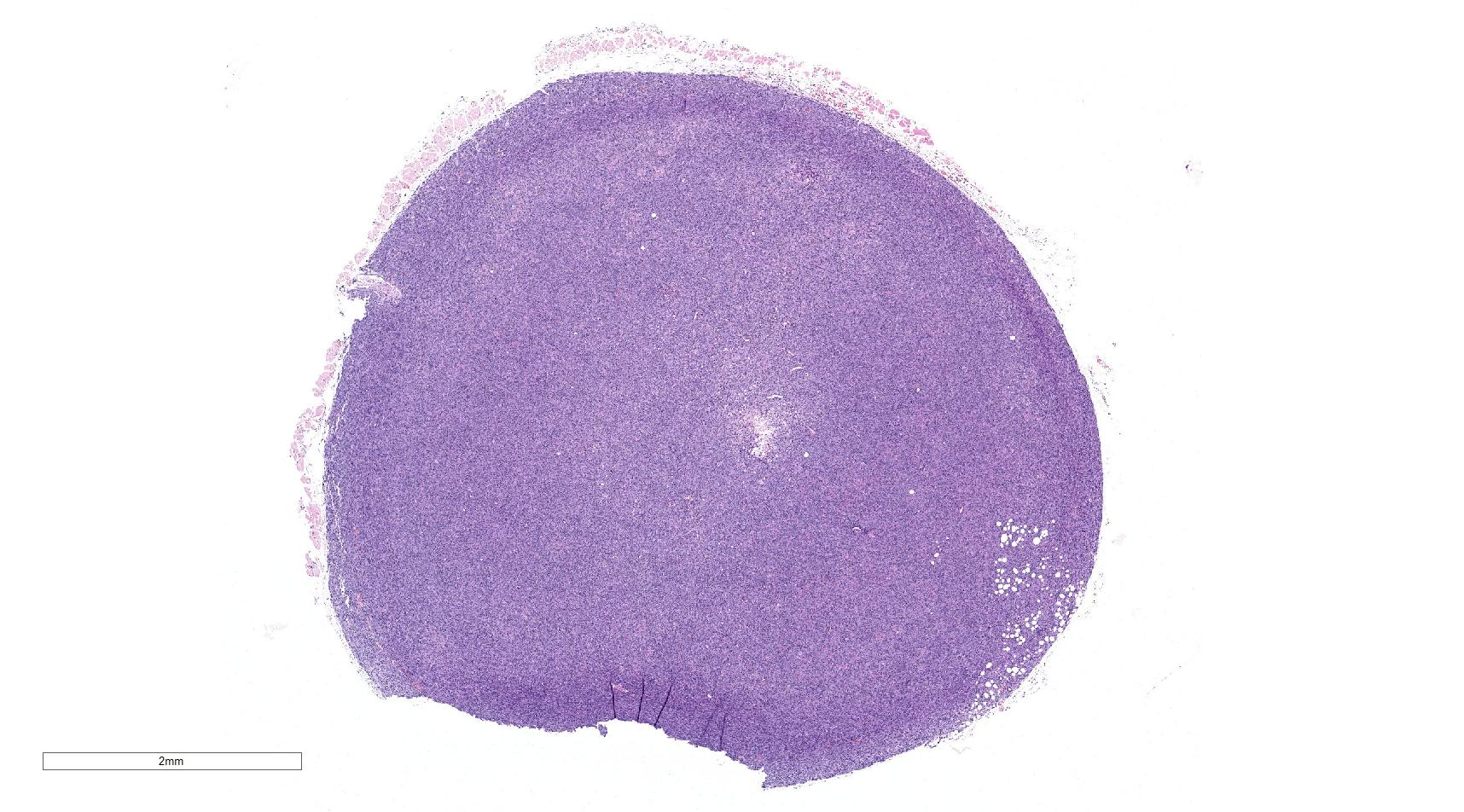


**Figure S16.** The H&E staining result of tumor in saline group.


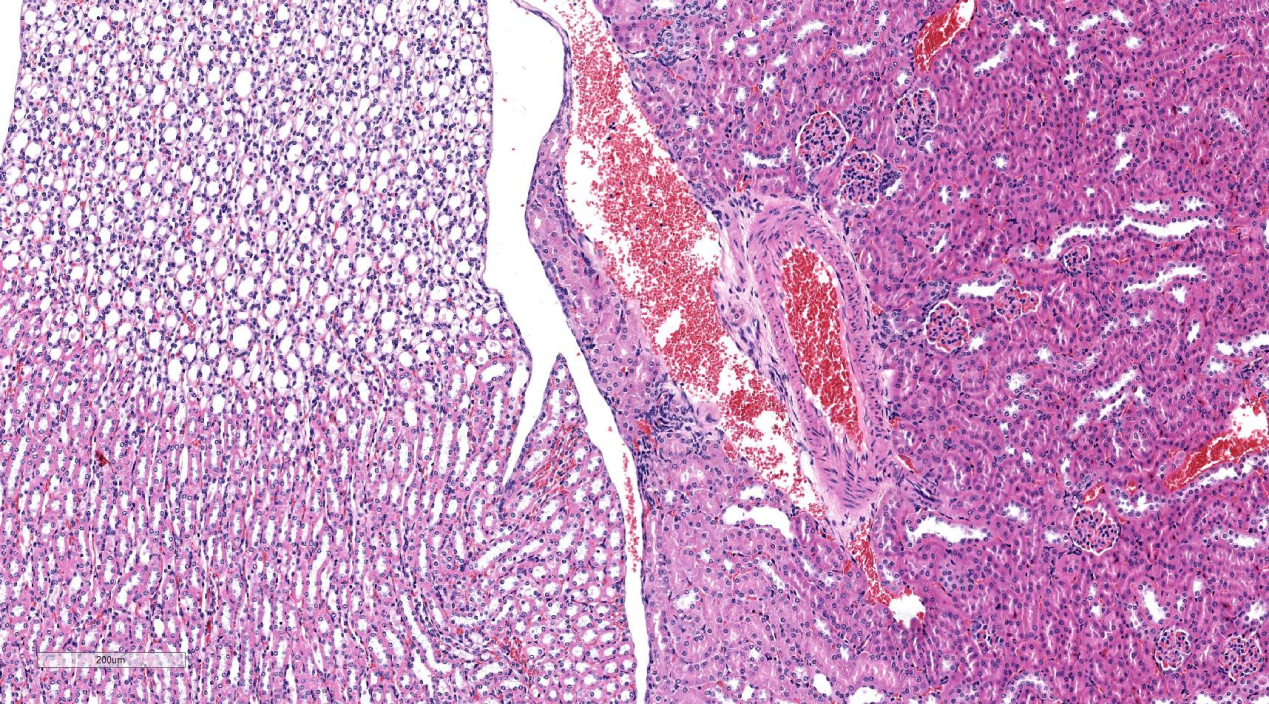


**Figure S17.** The H&E staining result of kidney in saline group.


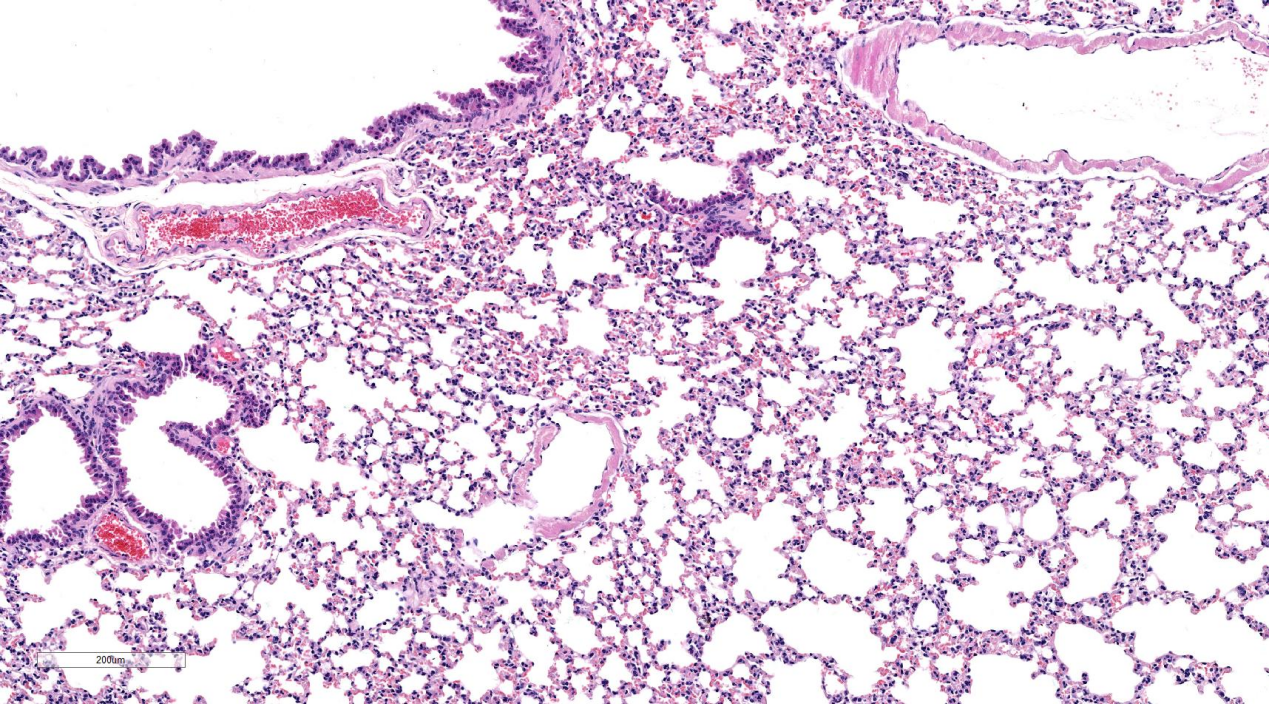


**Figure S18.** The H&E staining result of lung in saline group.


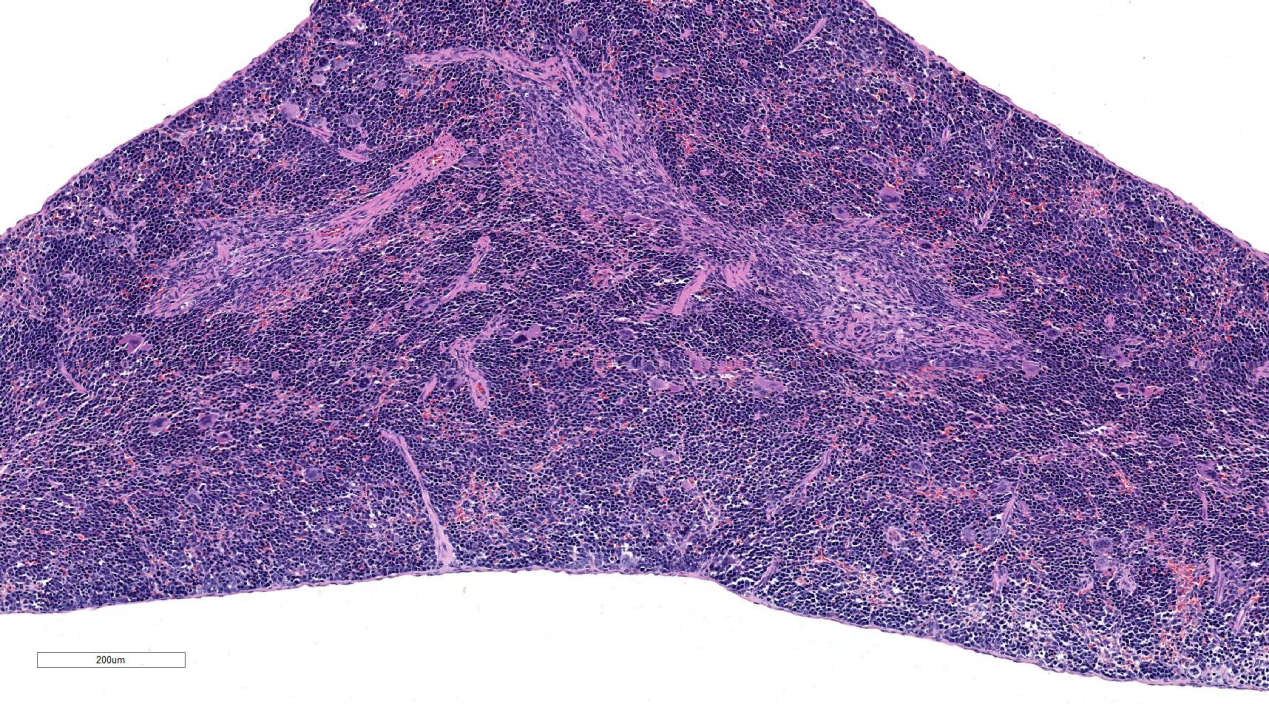


**Figure S17.** The H&E staining result of spleen in saline group.

**Figure S19.** The H&E staining result of spleen in saline group.


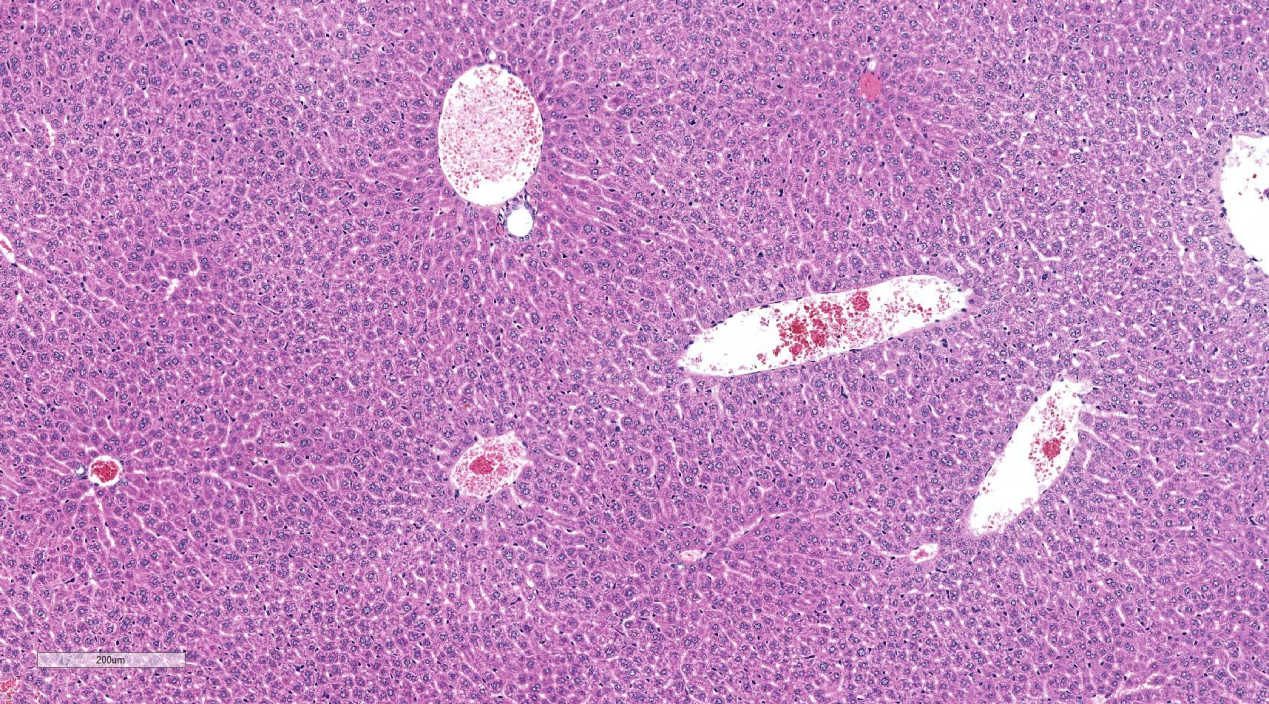


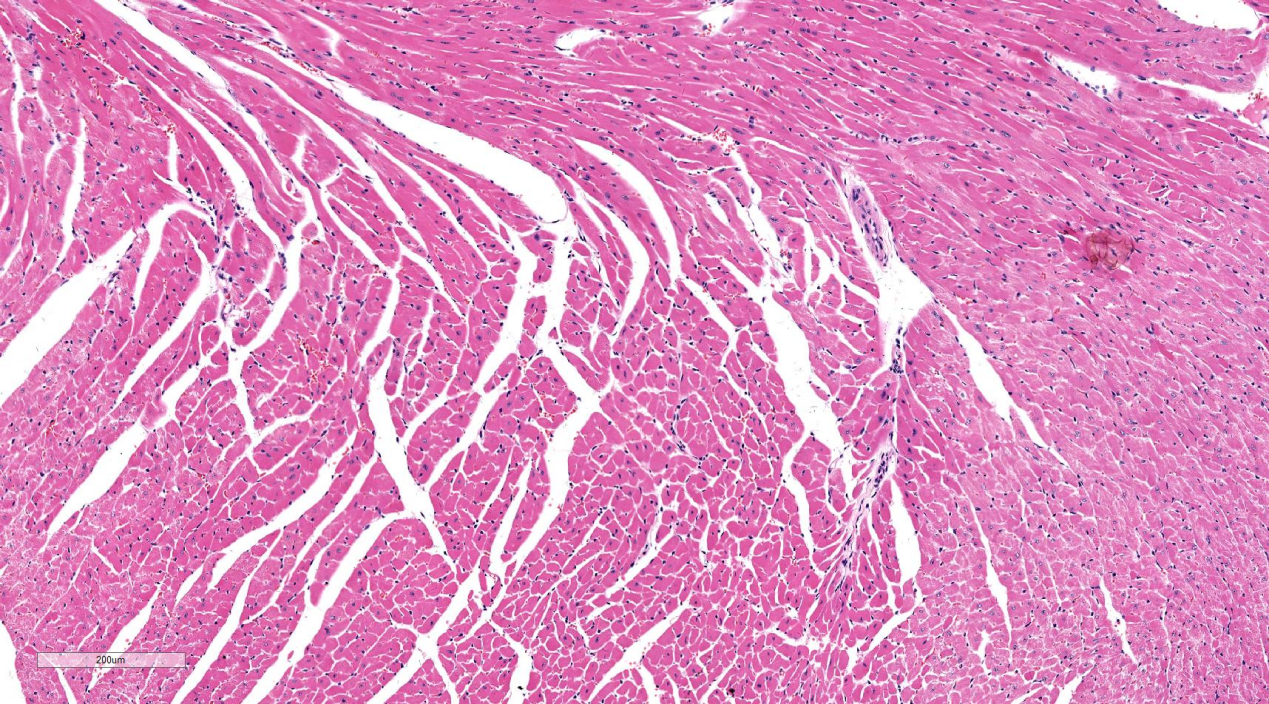
**Figure S20.** The H&E staining result of liver in saline group.

**Figure S21.** The H&E staining result of heart in saline group.


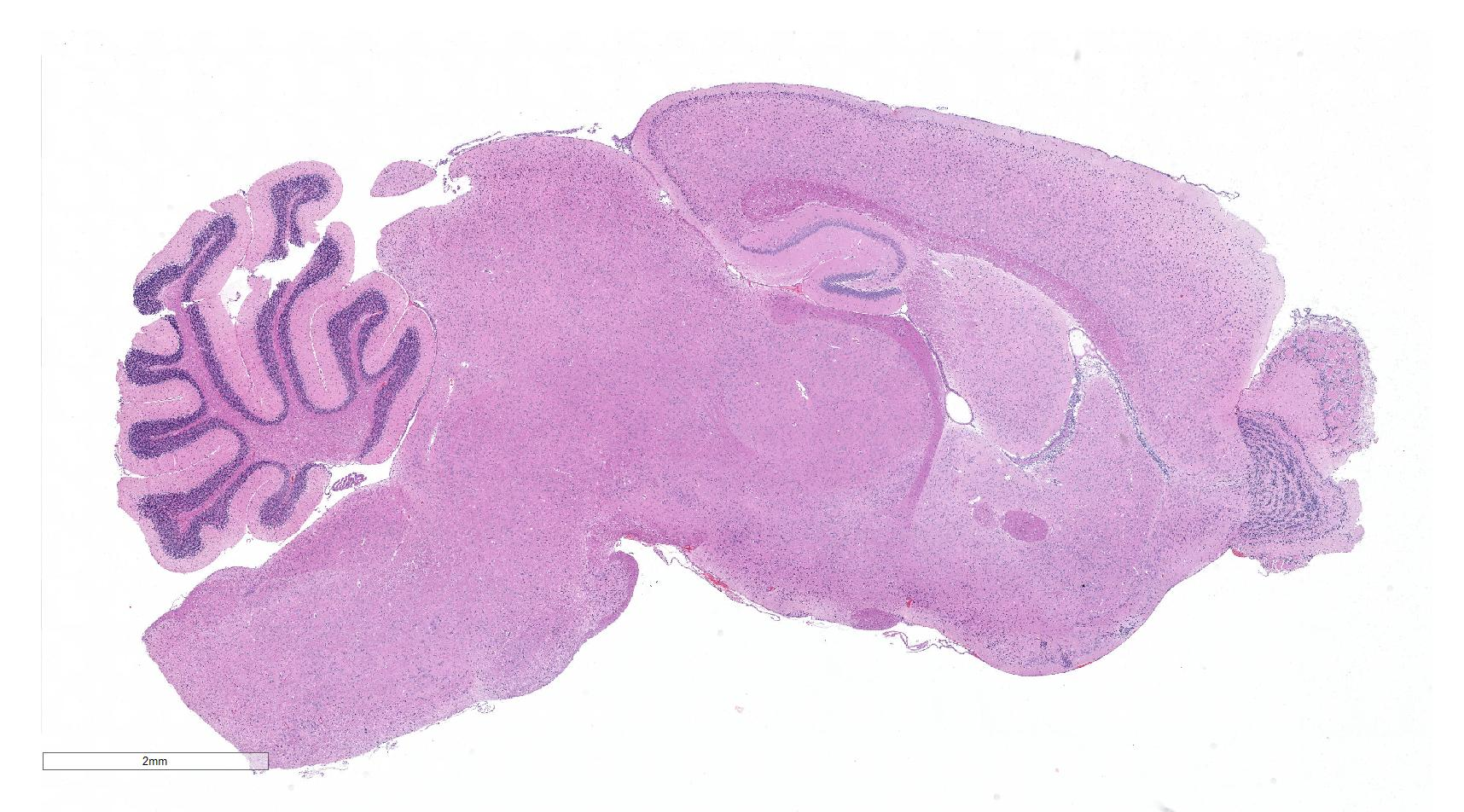


**Figure S22.** The H&E staining result of brain in saline group.


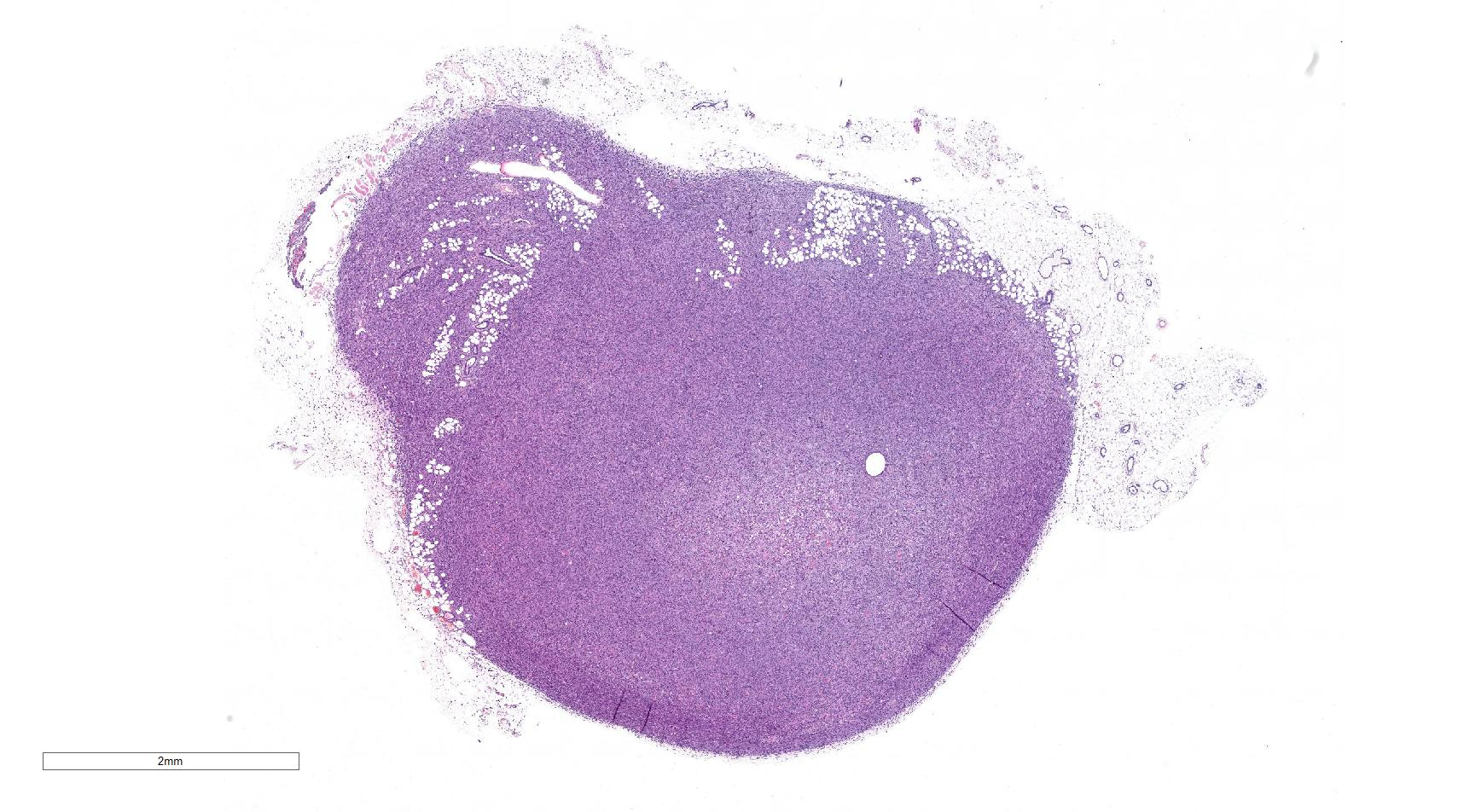


**Figure S23.** The H&E staining result of tumor in ^177^Lu-only group.


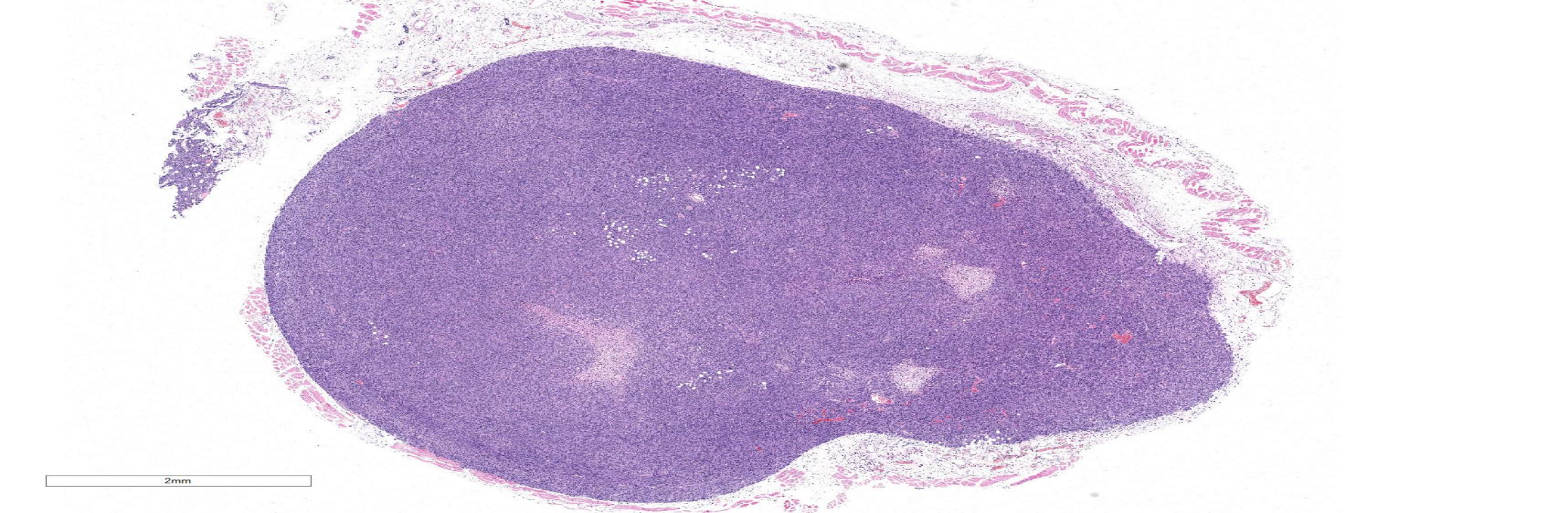


**Figure S24.** The H&E staining result of tumor in NY003-only group.


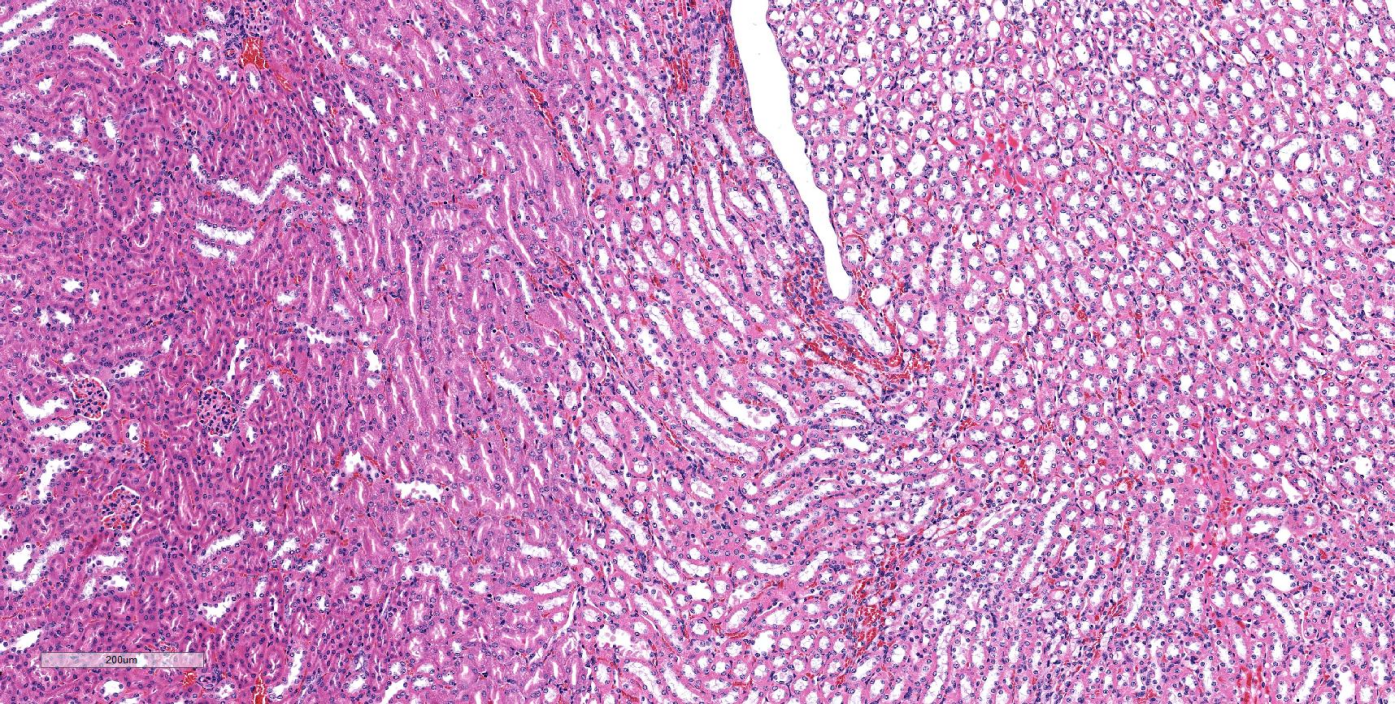


**Figure S25.** The H&E staining result of kidney in ^177^Lu-only group.


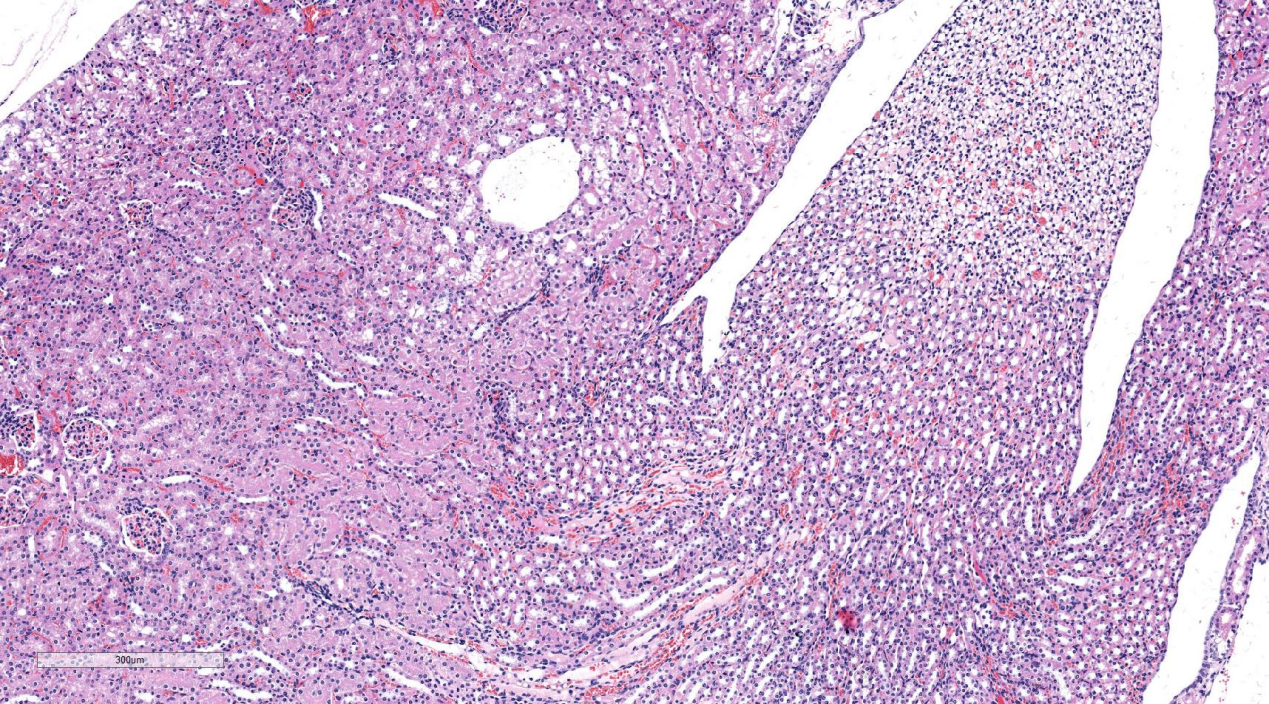


**Figure S26.** The H&E staining result of kidney in NY003-only group.


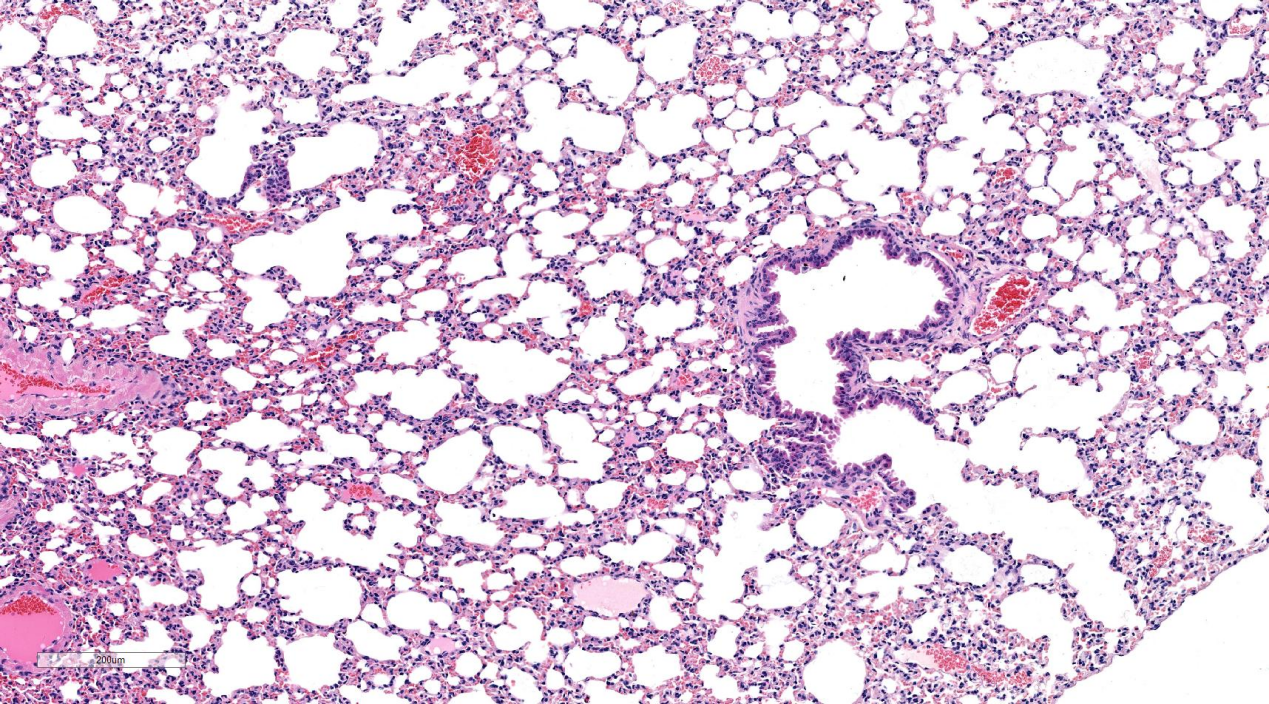


**Figure S10** The H&E staining result of lung in ^177^Lu-only group.

**Figure S27.** The H&E staining result of lung in ^177^Lu-only group.


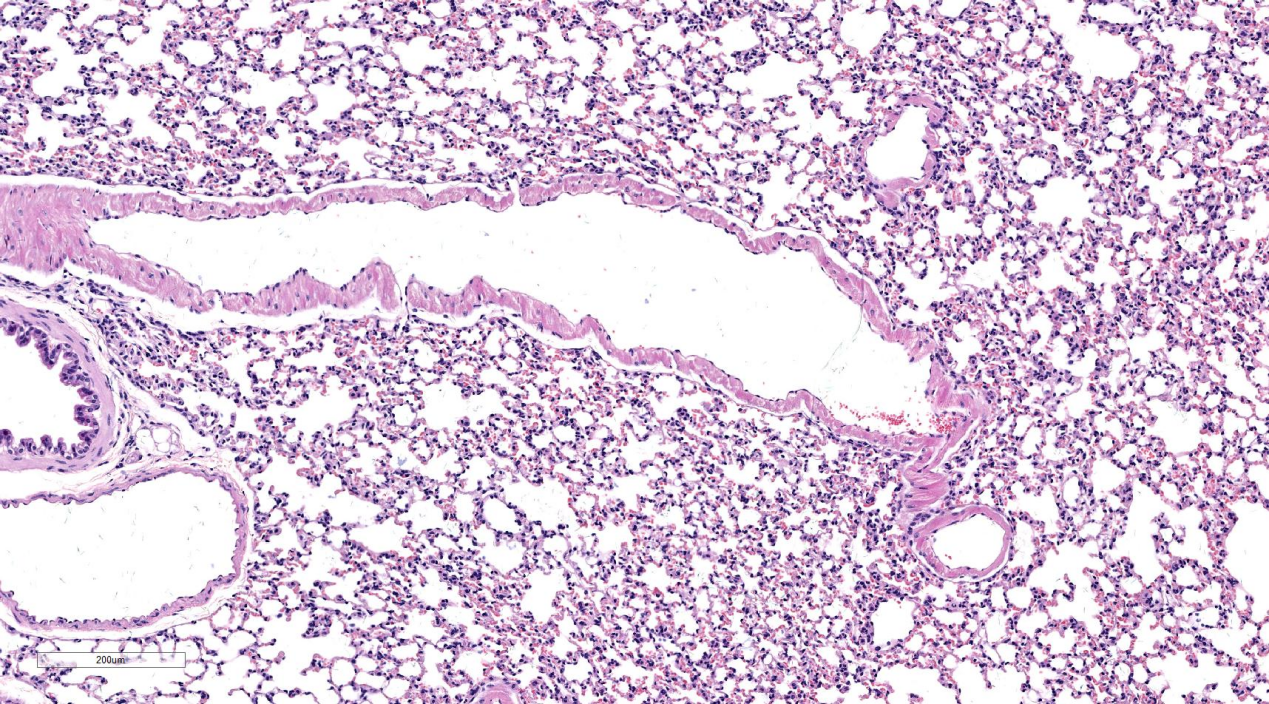


**Figure S11.** The H&E staining result of lung in NY003-only group.

**Figure S11.** The H&E staining result of lung in NY003-only group.

**Figure S28.** The H&E staining result of lung in NY003-only group.


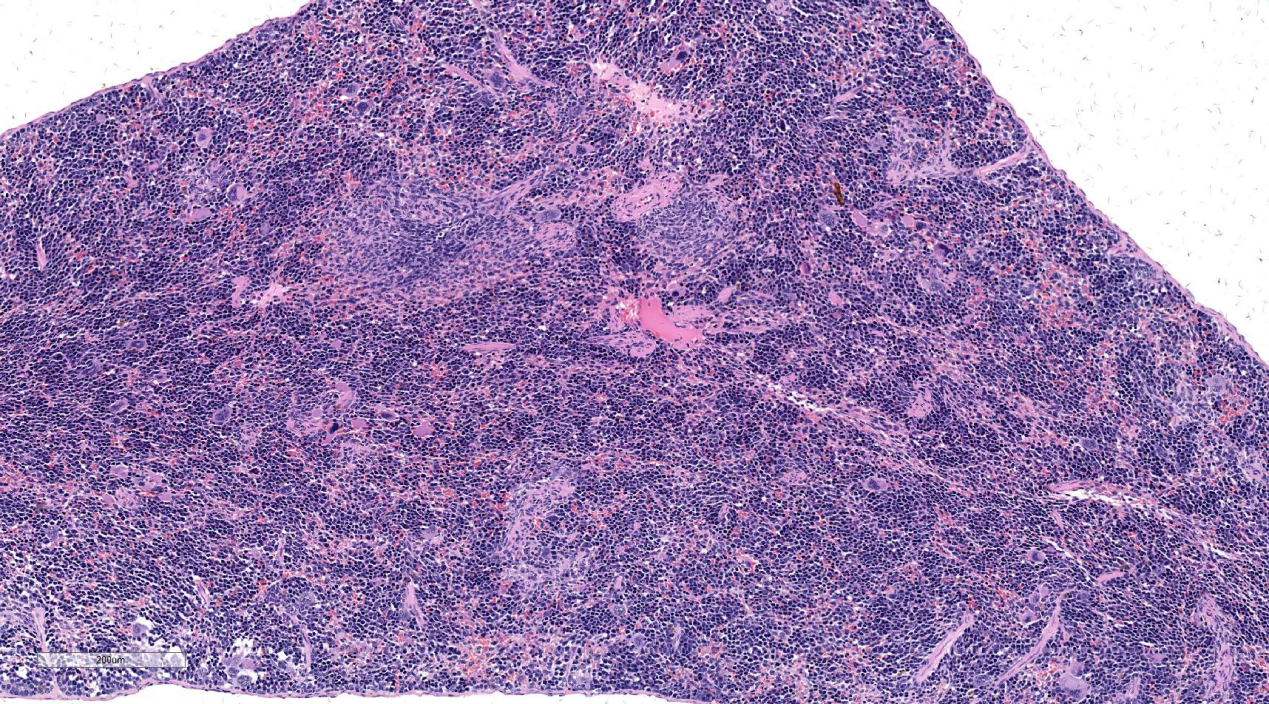


**Figure S29.** The H&E staining result of spleen in ^177^Lu-only group.


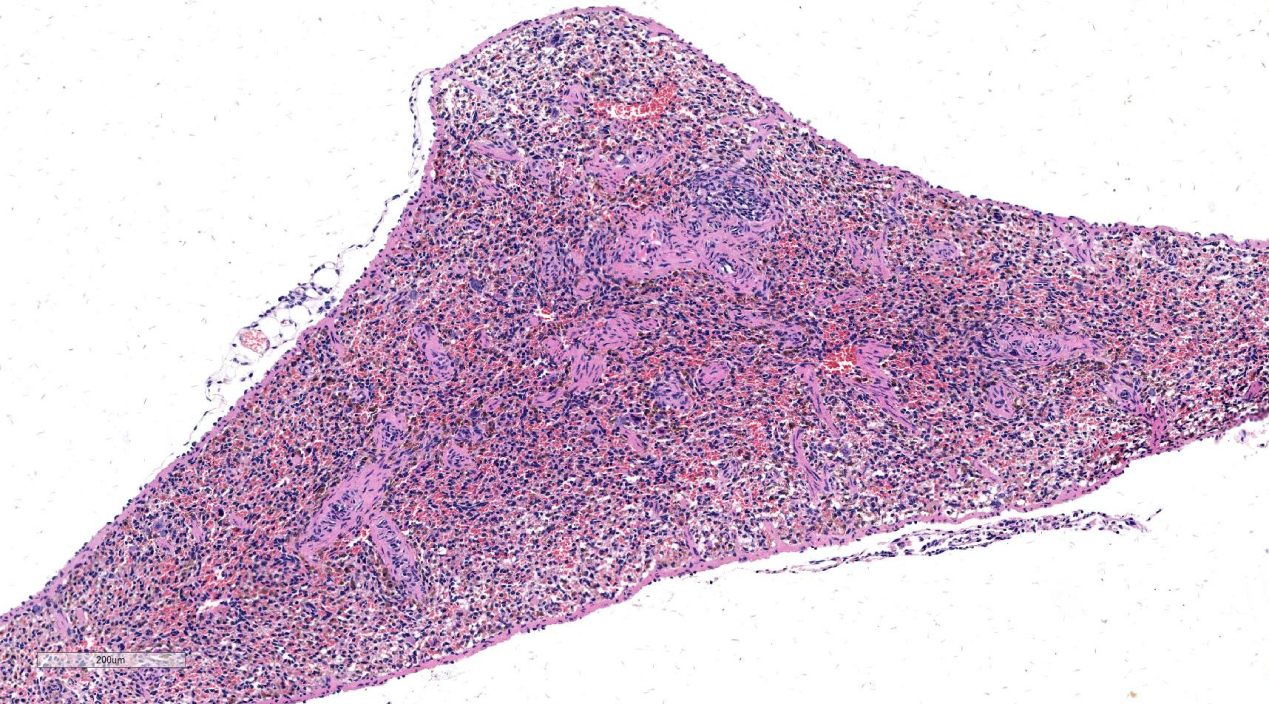


**Figure S30.** The H&E staining result of spleen in NY003-only group.


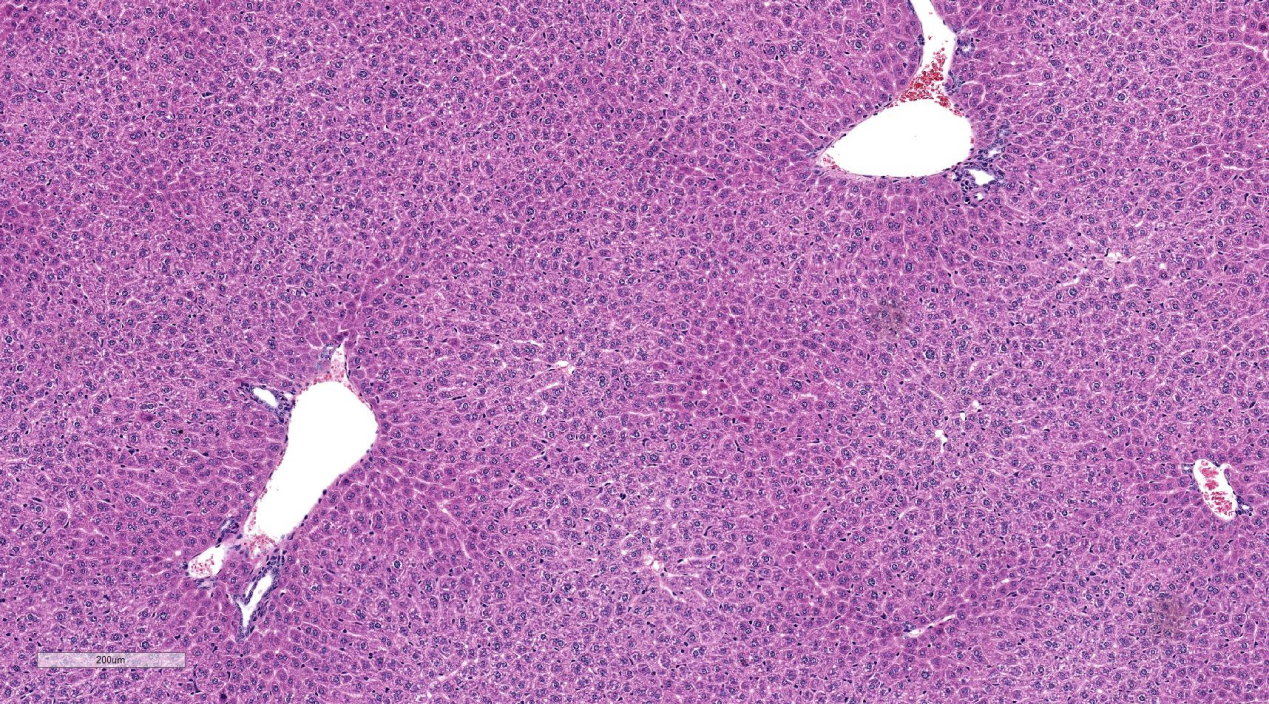


**Figure S31.** The H&E staining result of liver in ^177^Lu-only group.


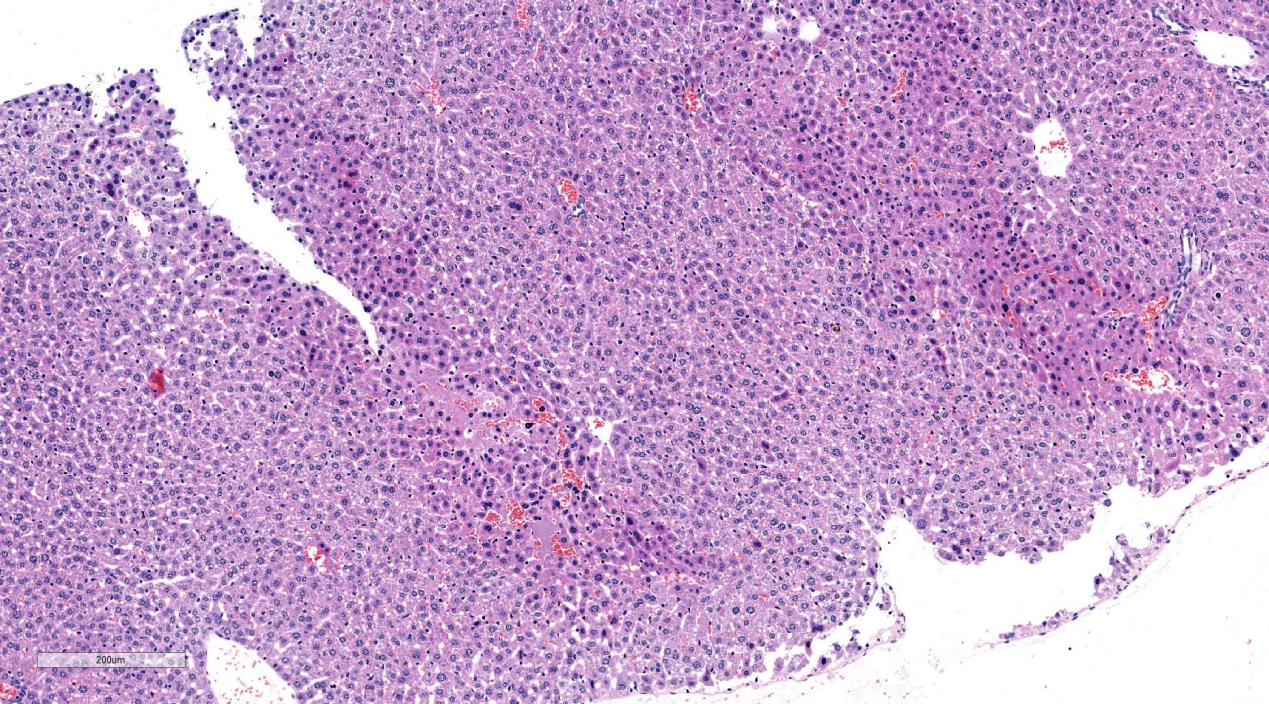


**Figure S32.** The H&E staining result of liver in NY003-only group.


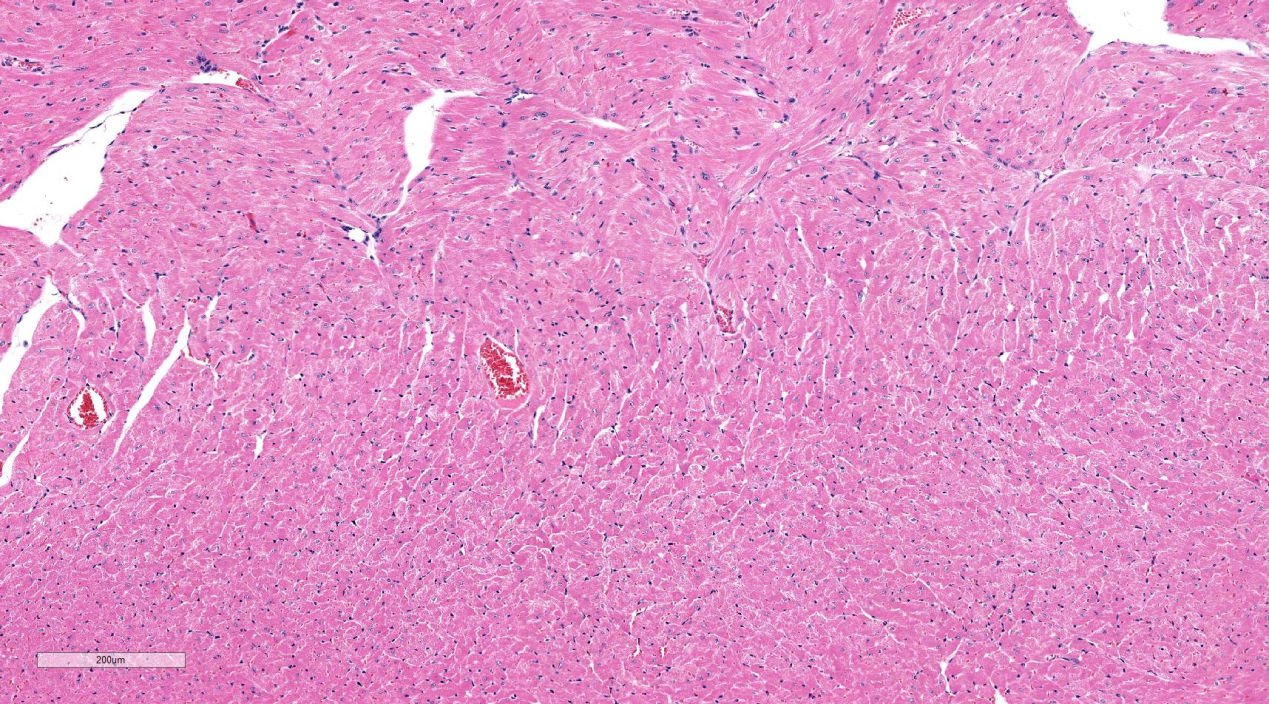


**Figure S33.** The H&E staining result of heart in ^177^Lu-only group.


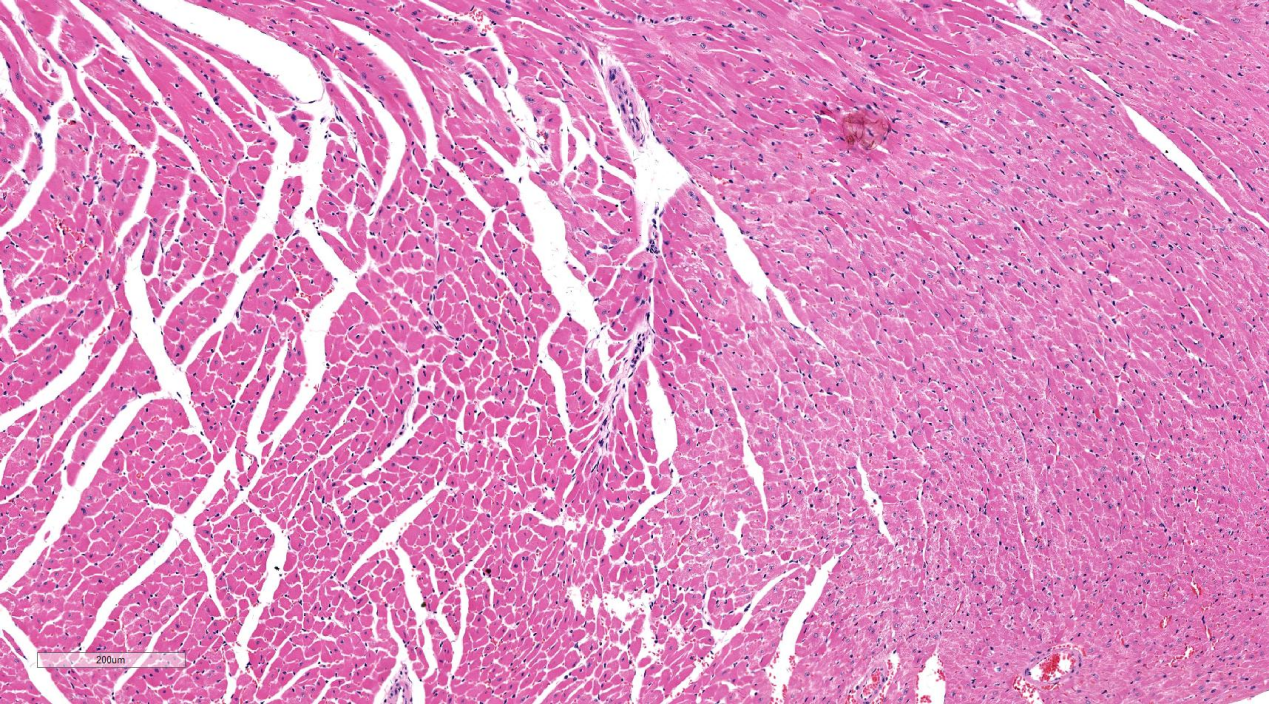


**Figure S34.** The H&E staining result of heart in NY003-only group.


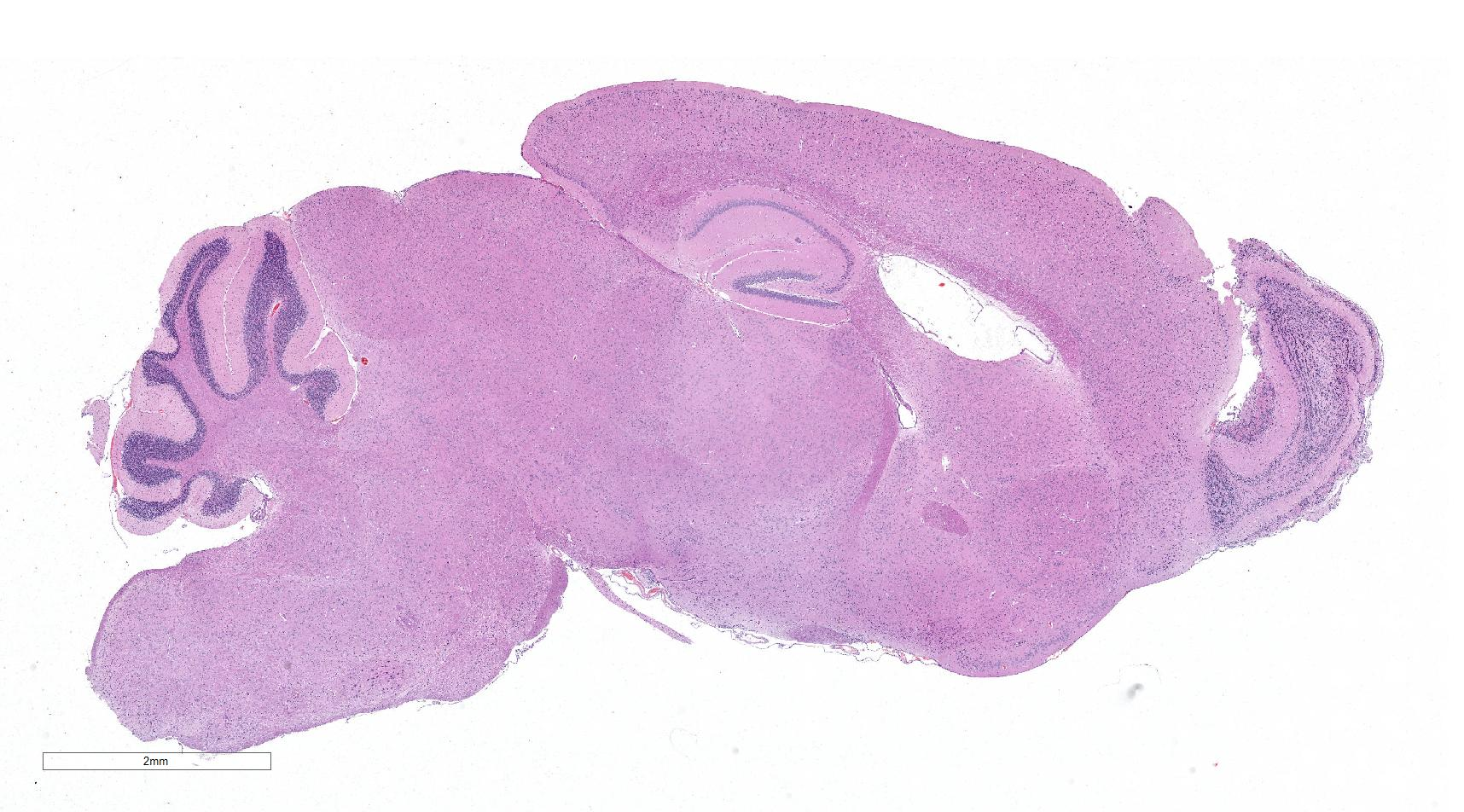


**Figure S35.** The H&E staining result of brain in ^177^Lu-only group.


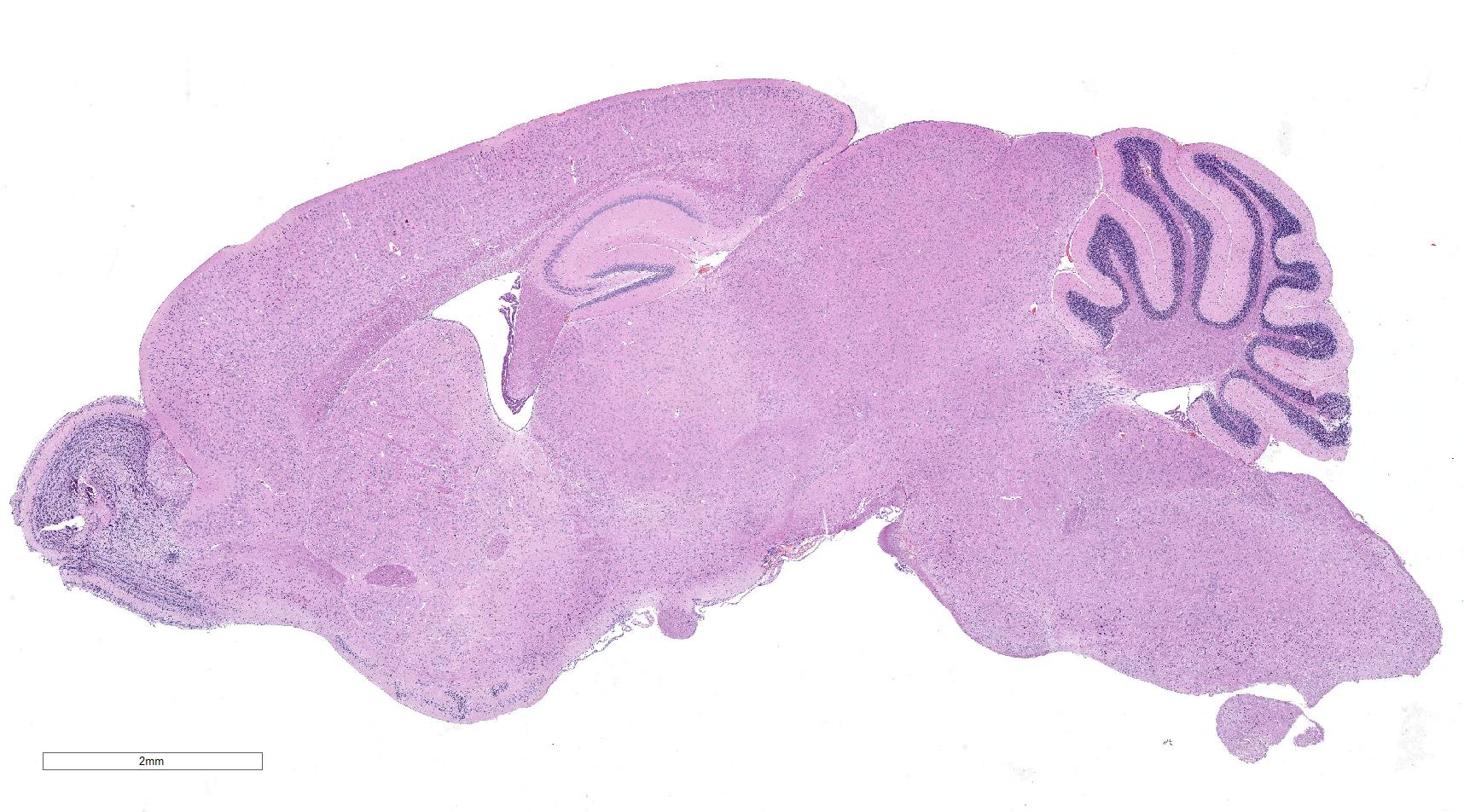


**Figure S36.** The H&E staining result of brain in NY003-only group.


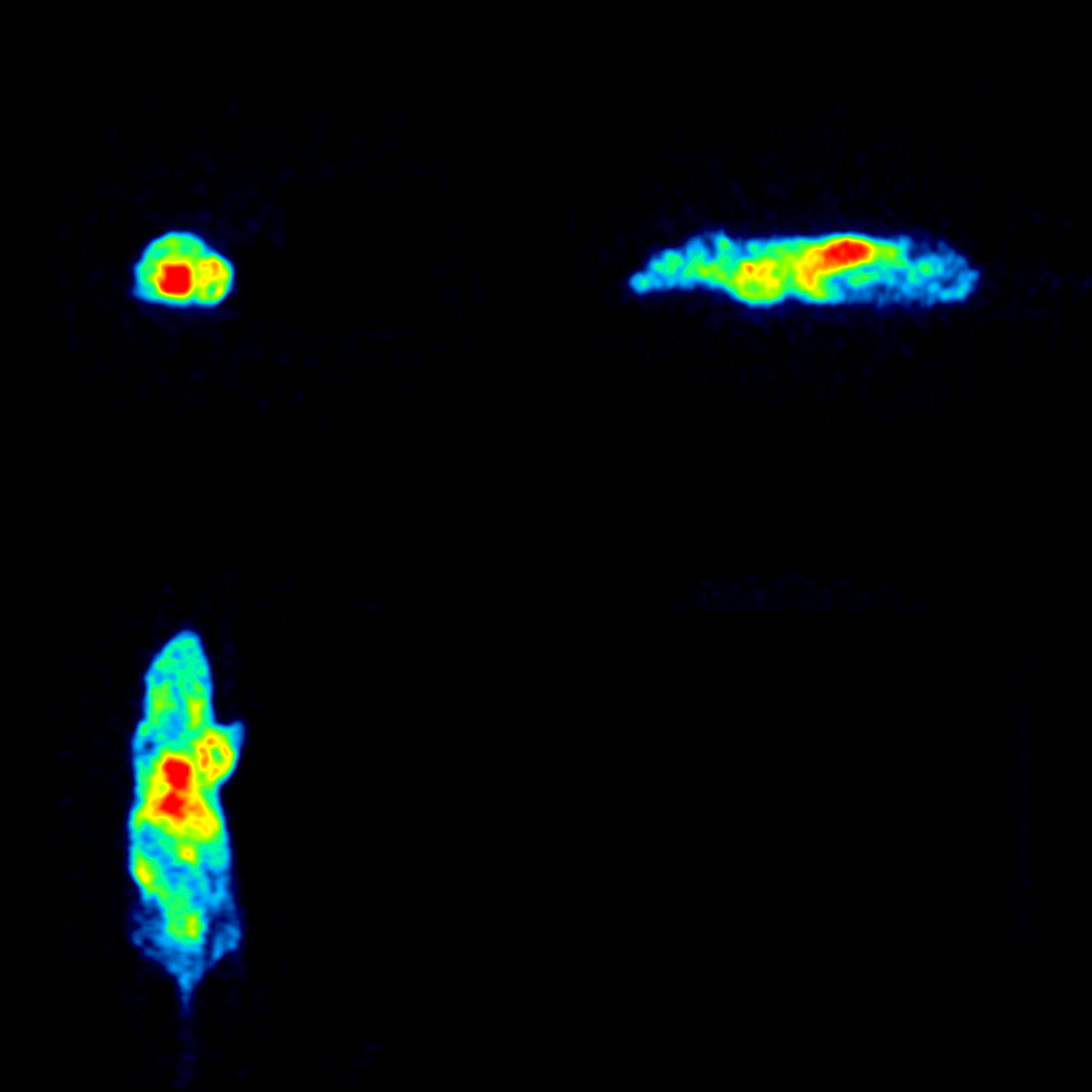


**Figure S37.** The section images of Micro-PET at 7 h p.i..


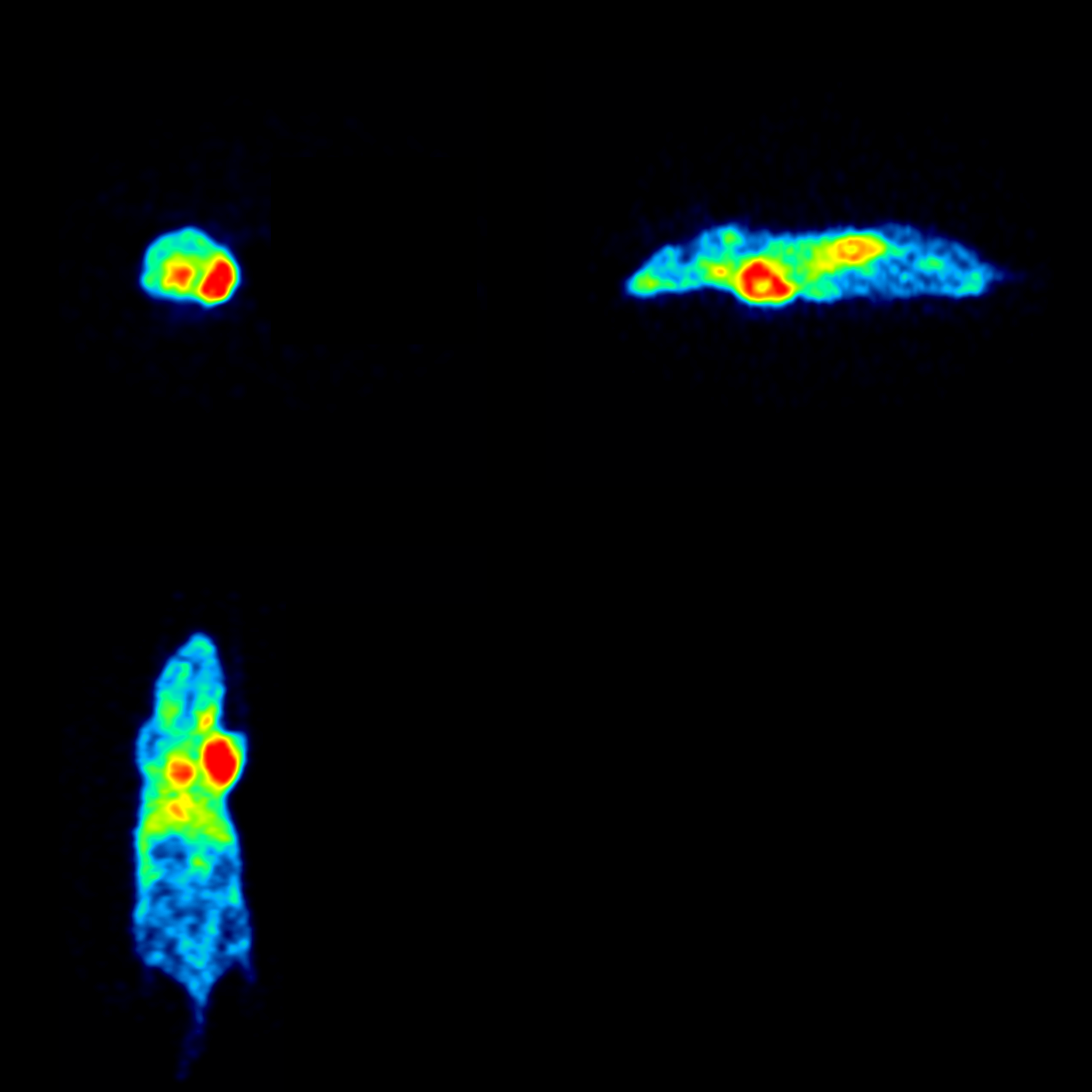


**Figure S38.** The section images of Micro-PET at 24 h p.i..


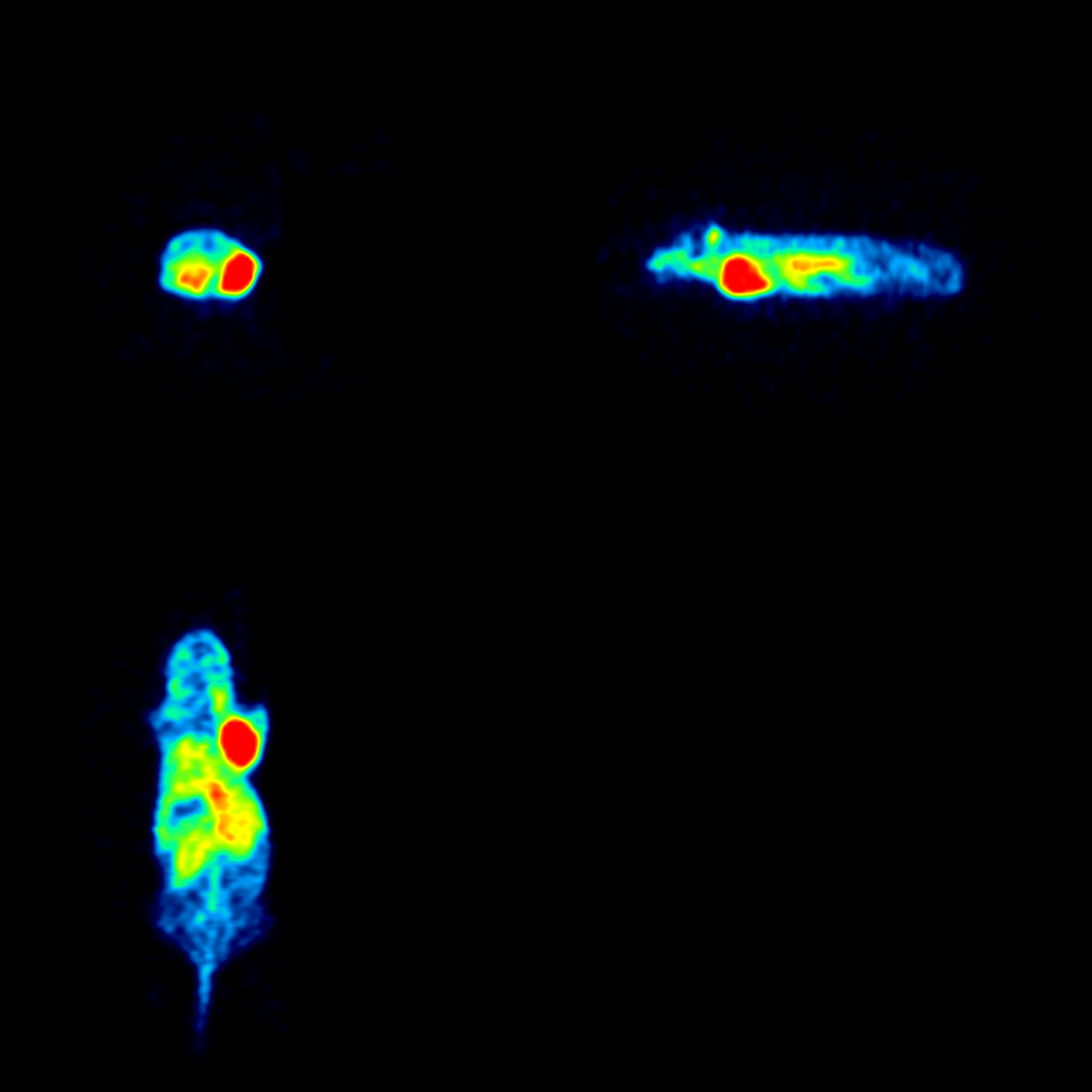


**Figure S39.** The section images of Micro-PET at 48 h p.i..


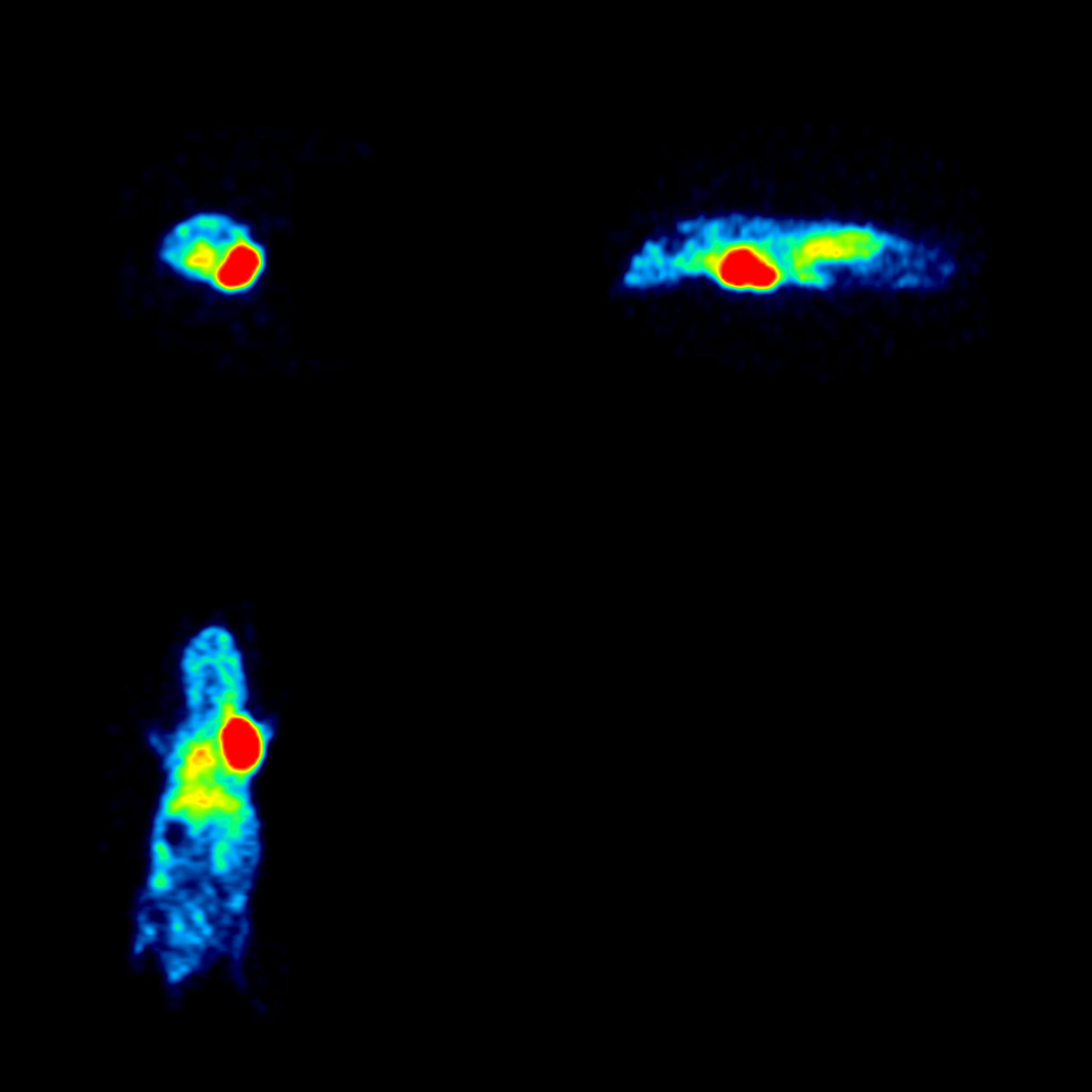


**Figure S40.** The section images of Micro-PET at 72 h p.i..


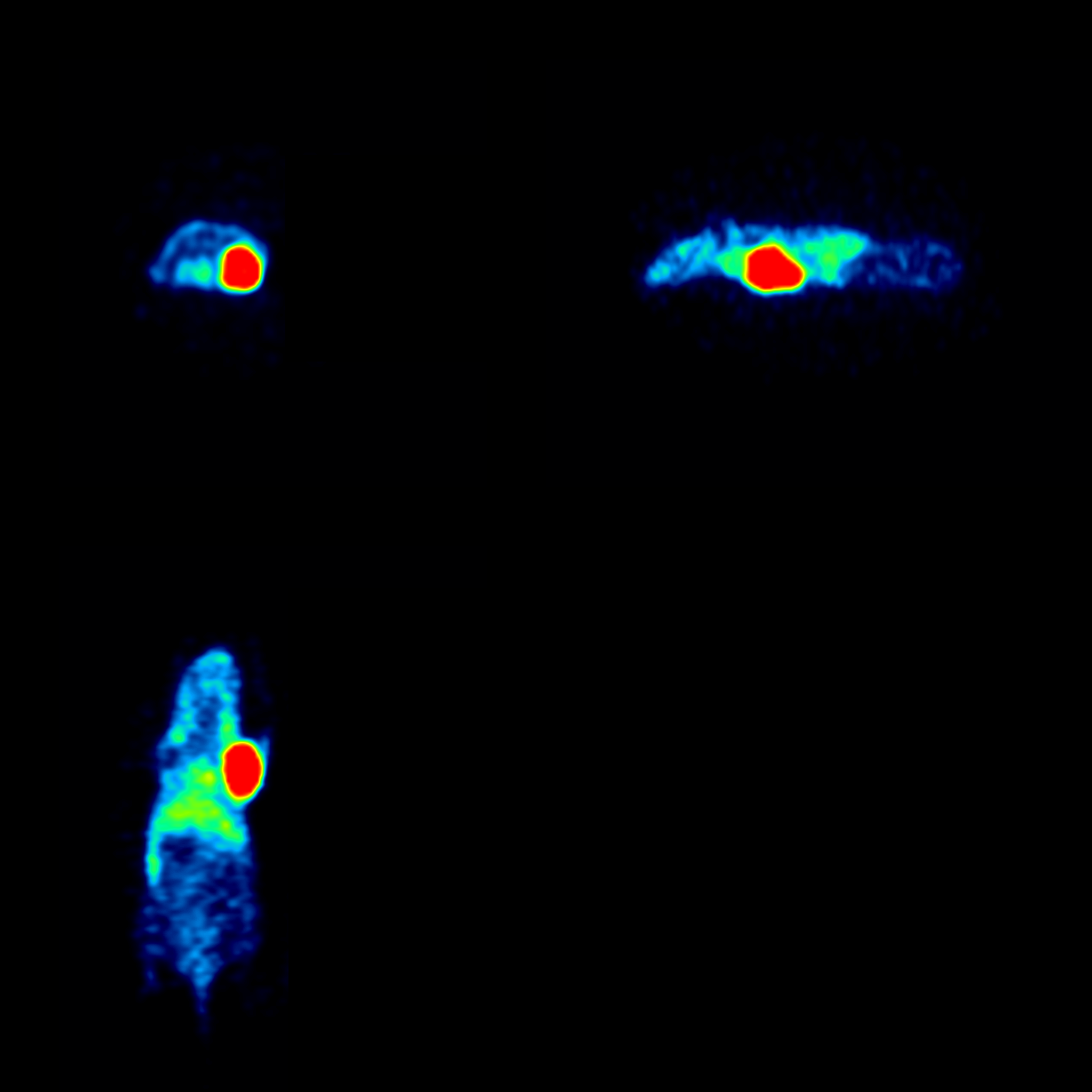


**Figure S41.** The section images of Micro-PET at 96 h p.i..


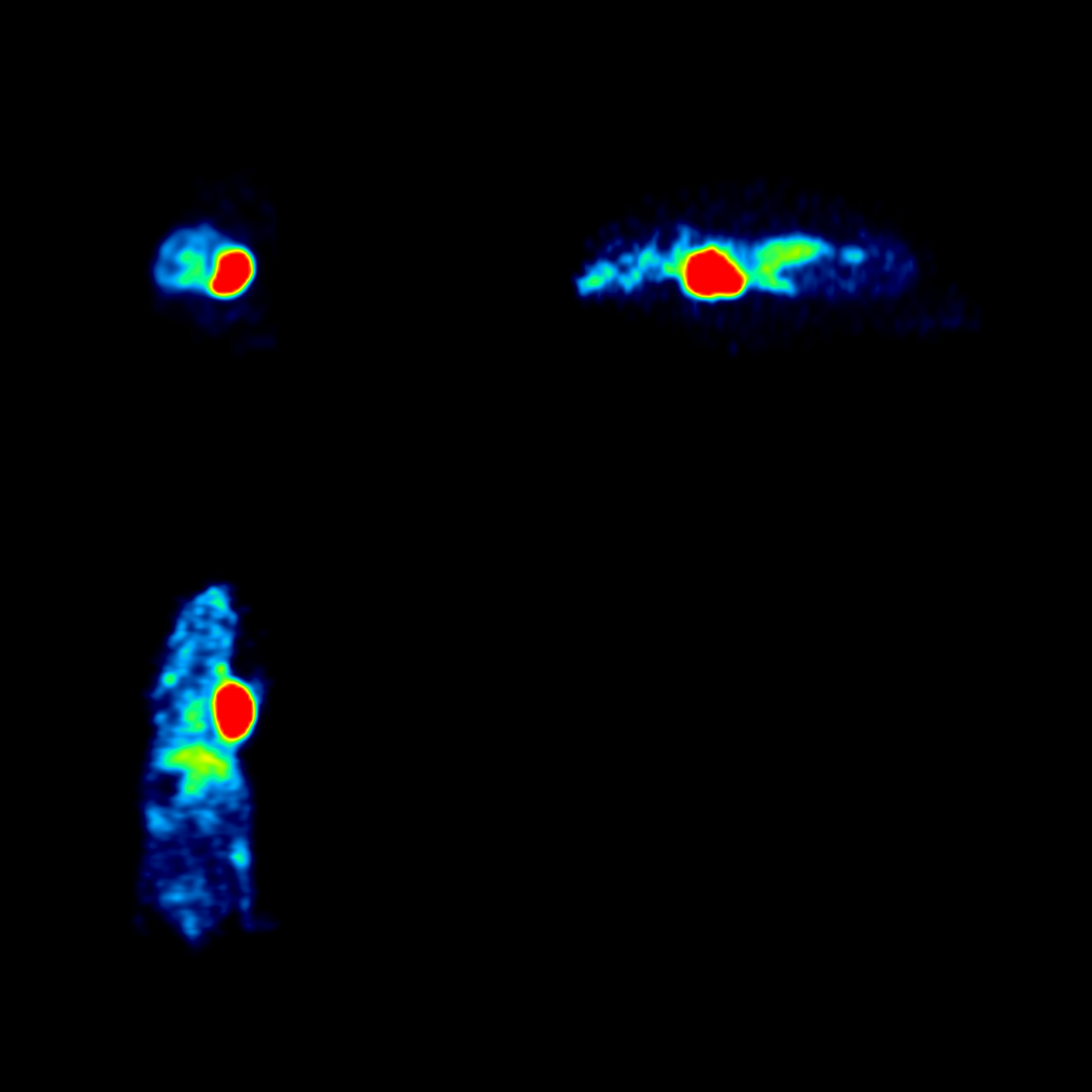


**Figure S42.** The section images of Micro-PET at 144 h p.i..


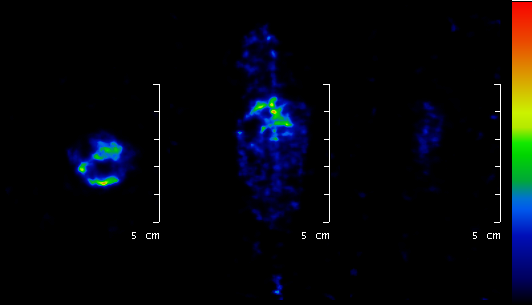


**Figure S43.** The section images of SPECT at 1 h p.i..


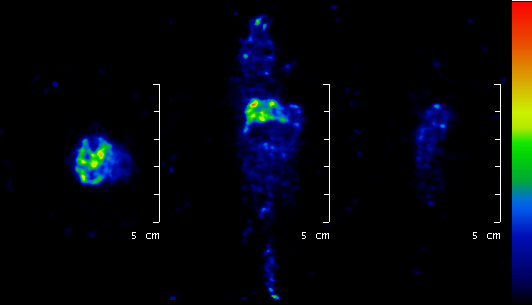


**Figure S44.** The section images of SPECT at 4 h p.i..


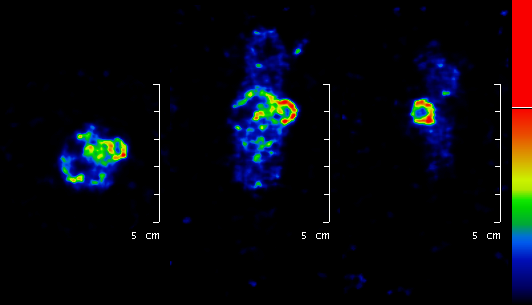


**Figure S45.** The section images of SPECT at 12 h p.i..


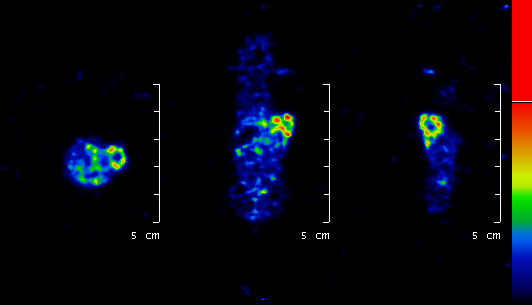


**Figure S46.** The section images of SPECT at 24 h p.i..


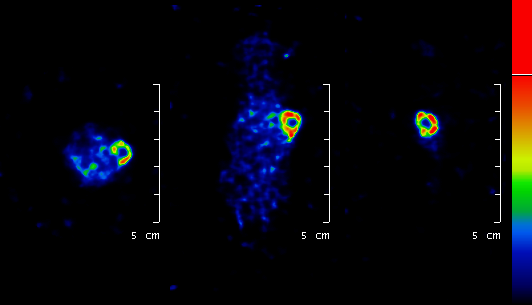


**Figure S47.** The section images of SPECT at 48 h p.i..


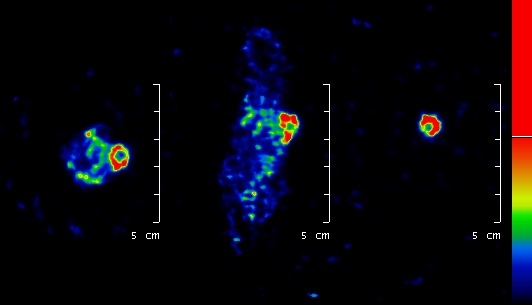


**Figure S48.** The section images of SPECT at 72 h p.i..


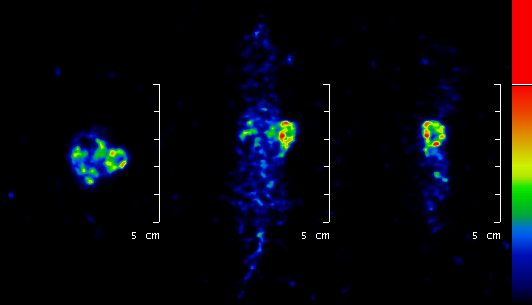


**Figure S49.** The section images of SPECT at 96 h p.i..
